# Draft genome assembly of the invasive cane toad, *Rhinella marina*

**DOI:** 10.1093/gigascience/giy095

**Published:** 2018-08-07

**Authors:** Richard J Edwards, Daniel Enosi Tuipulotu, Timothy G Amos, Denis O'Meally, Mark F Richardson, Tonia L Russell, Marcelo Vallinoto, Miguel Carneiro, Nuno Ferrand, Marc R Wilkins, Fernando Sequeira, Lee A Rollins, Edward C Holmes, Richard Shine, Peter A White

**Affiliations:** 1 School of Biotechnology and Biomolecular Sciences, University of New South Wales, Sydney, NSW, 2052, Australia; 2 Sydney School of Veterinary Science, Faculty of Science, University of Sydney, Camperdown, NSW, 2052, Australia; 3 School of Life and Environmental Sciences, Centre for Integrative Ecology, Deakin University, Geelong, VIC, 3216, Australia; 4 Bioinformatics Core Research Group, Deakin University, Geelong, VIC, 3216, Australia; 5 Ramaciotti Centre for Genomics, University of New South Wales, Sydney, NSW, 2052, Australia; 6 CIBIO/InBIO, Centro de Investigação em Biodiversidade e Recursos Genéticos, Universidade do Porto, Vairão, Portugal; 7 Laboratório de Evolução, Instituto de Estudos Costeiros (IECOS), Universidade Federal do Pará, Bragança, Pará, Brazil; 8 Departamento de Biologia, Faculdade de Ciências, Universidade do Porto, Porto, Portugal; 9 Department of Zoology, Faculty of Sciences, University of Johannesburg, Auckland Park, South Africa; 10 Evolution and Ecology Research Centre, School of Biological Earth and Environmental Sciences, University of New South Wales, Sydney, NSW, 2052, Australia; 11 Marie Bashir Institute for Infectious Diseases and Biosecurity, Charles Perkins Centre, School of Life and Environmental Sciences and Sydney Medical School, University of Sydney, Sydney, NSW, 2006, Australia; 12 School of Life and Environmental Sciences, Faculty of Science, University of Sydney, Camperdown, NSW, 2006, Australia

**Keywords:** cane toad, *Rhinella marina*, sequencing, hybrid assembly, genome, annotation

## Abstract

**Background:**

The cane toad (*Rhinella marina* formerly *Bufo marinus*) is a species native to Central and South America that has spread across many regions of the globe. Cane toads are known for their rapid adaptation and deleterious impacts on native fauna in invaded regions. However, despite an iconic status, there are major gaps in our understanding of cane toad genetics. The availability of a genome would help to close these gaps and accelerate cane toad research.

**Findings:**

We report a draft genome assembly for *R. marina*, the first of its kind for the Bufonidae family. We used a combination of long-read Pacific Biosciences RS II and short-read Illumina HiSeq X sequencing to generate 359.5 Gb of raw sequence data. The final hybrid assembly of 31,392 scaffolds was 2.55 Gb in length with a scaffold N50 of 168 kb. BUSCO analysis revealed that the assembly included full length or partial fragments of 90.6% of tetrapod universal single-copy orthologs (n = 3950), illustrating that the gene-containing regions have been well assembled. Annotation predicted 25,846 protein coding genes with similarity to known proteins in Swiss-Prot. Repeat sequences were estimated to account for 63.9% of the assembly.

**Conclusions:**

The *R. marina* draft genome assembly will be an invaluable resource that can be used to further probe the biology of this invasive species. Future analysis of the genome will provide insights into cane toad evolution and enrich our understanding of their interplay with the ecosystem at large.

## Data Description

### Introduction

**Figure 1:**
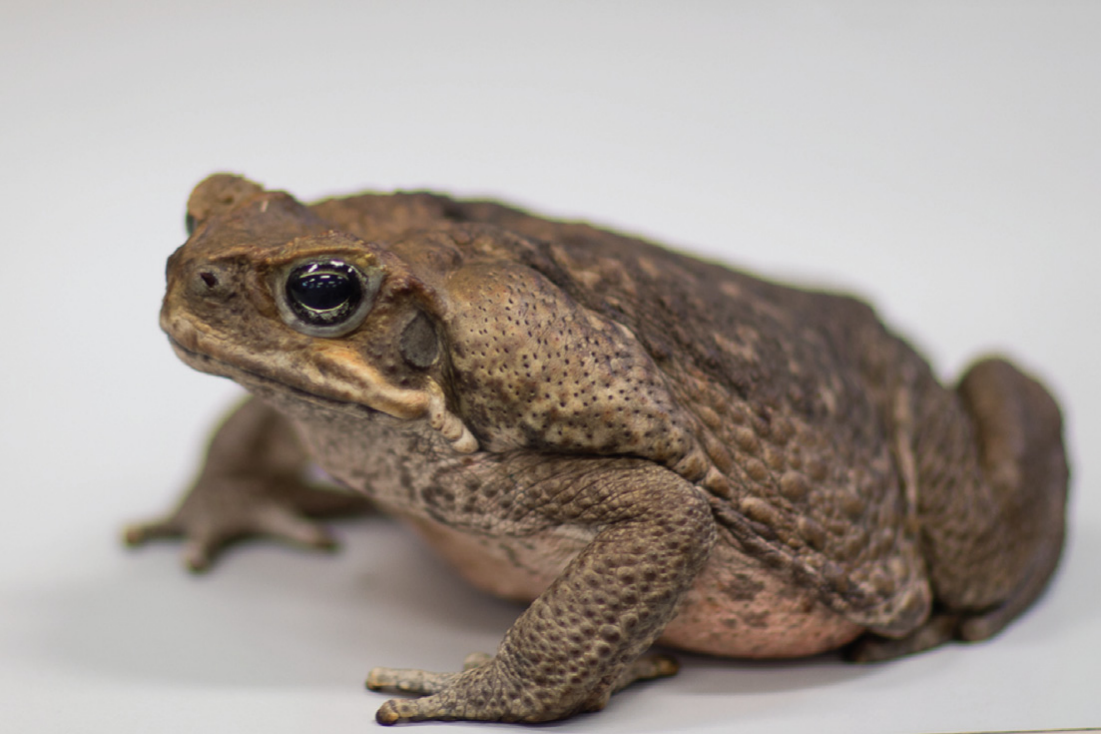
An adult cane toad, *Rhinella marina*.

**Figure 2:**
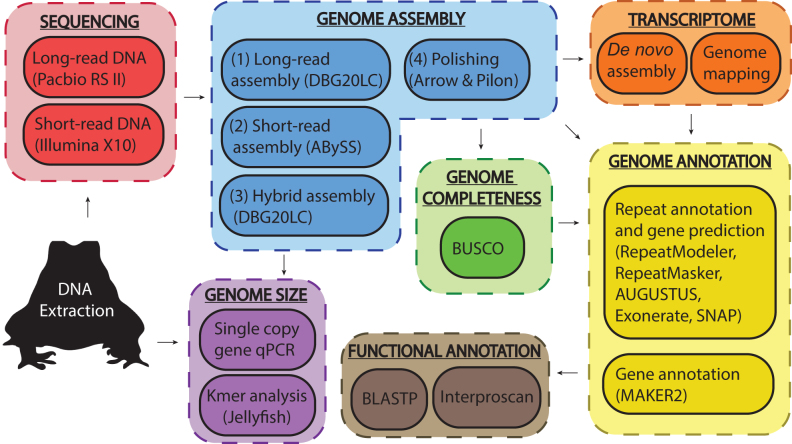
Schematic overview of project workflow. A summary of the experimental methods used for sequencing, assembly, annotation, and size estimation of the cane toad genome. Transcriptome data (orange segment) were obtained from our previous study [18].

The cane toad (*Rhinella marina* formerly *Bufo marinus*) (Fig. 1) is a true toad (Bufonidae) native to Central and South America that has been introduced to many areas across the globe [1]. Since its introduction into Queensland in 1935, the cane toad has spread widely and now occupies more than 1.2 million square kilometers of the Australian continent, fatally poisoning predators such as the northern quoll, freshwater crocodiles, and several species of native lizards and snakes [1-5]. The ability of cane toads to kill predators with toxic secretions has contributed to the success of their invasion [1]. To date, research on cane toads has focused primarily on ecological impacts, rapid evolution of phenotypic traits, and population genetics using neutral markers [6, 7], with limited knowledge of the genetic changes that allow the cane toad to thrive in the Australian environment [8-11]. A reference genome will be useful for studying loci subject to rapid evolution and could provide valuable insights into how invasive species adapt to new environments. Amphibian genomes have a preponderance of repetitive DNA [12, 13], confounding assembly with the limited read lengths of first- and second-generation sequencing technologies. Here, we employ a hybrid assembly of Pacific Biosciences (PacBio) long reads and Illumina short reads (Fig. 2) to overcome assembly challenges presented by the repetitive nature of the cane toad genome. Using this approach, we assembled a draft genome of *R. marina* that is comparable in contiguity and completeness to other published anuran genomes [14-17]. We used our previously published transcriptomic data [18] and other published anuran sequences to annotate the genome. Our draft cane toad assembly will serve as a reference for genetic and evolutionary studies and provides a template for continued refinement with additional sequencing efforts.

### Sample collection, library construction, and sequencing

Adult female cane toads were collected by hand from Forrest River in Oombulgurri, WA (15.1818^o^S, 127.8413^o^E) in June 2015. Toads were placed in individual damp cloth bags and transported by plane to Sydney, NSW, before they were anaesthetized by refrigeration for 4 hours and killed by subsequent freezing. High-molecular-weight genomic DNA (gDNA) was extracted from the liver of a single female using the genomic-tip 100/G kit (Qiagen, Hilden, Germany). This was performed with supplemental RNase (Astral Scientific, Taren Point, Australia) and proteinase K (NEB, Ipswich, MA, USA) treatment, as per the manufacturer's instructions. Isolated gDNA was further purified using AMPure XP beads (Beckman Coulter, Brea, CA, USA) to eliminate sequencing inhibitors. DNA quantity was assessed using the Quanti-iT PicoGreen dsDNA kit (Thermo Fisher Scientific, Waltham, MA, USA). DNA purity was calculated using a Nanodrop spectrophotometer (Thermo Fisher Scientific), and molecular integrity was assessed using pulse-field gel electrophoresis.

For short-read sequencing, a paired-end library was constructed from the gDNA using the TruSeq polymerase chain reaction (PCR)-free library preparation kit (Illumina, San Diego, CA, USA). Insert sizes ranged from 200 to 800 bp. This library was sequenced (2 × 150 bp) on the HiSeq X Ten platform (Illumina) to generate approximately 282.9 Gb of raw data (Table 1). Illumina short sequencing reads were assessed for quality using FastQC v0.10.1 [19]. Low-quality reads were filtered and trimmed using Trimmomatic v0.36 [20] with a Q30 threshold (LEADING:30, TRAILING:30, SLIDINGWINDOW:4:30) and a minimum 100-bp read length, leaving 64.9% of the reads generated, of which 75.2% were in retained read pairs.

**Table 1:** Summary statistics of generated whole-genome shotgun sequencing data

Platform	Library type	Mean insert size (kb)	Mean read length (bp)	Number of reads	Number of bases (Gb)
HiSeqX (raw)	Paired-end	0.35	147.7	1857,762 ,090	282.92
**HiSeqX (filtered)**			**140.6**	**1,205,616,705**	**169.47**
PacBio RS II	SMRTbell	15–50	8,852	2,794,391	24.736
PacBio RS II	SMRTbell	15–50	9,085	595,447	5.409
PacBio RS II	SMRTbell	15–50	10,432	1,867,543	19.482
PacBio RS II	SMRTbell	20–50	10,834	2,487,852	26.952
**PacBio Total**			**9,887**	**7,745** **,233**	**76.58**
**PacBio Unique** ^a^			**10** **987**	**6,167**,**714**	**67.77**

Bold rows indicate data used for assembly.

a Longest read per sequenced molecule (single-molecule real-time zero-mode waveguide- ZMW).

For long-read sequencing, we used the single-molecule real-time (SMRT) sequencing technology (PacBio, Menlo Park, CA, USA). Four SMRTbell libraries were prepared from gDNA using the SMRTBell template preparation kit 1.0 (PacBio). To increase subread length, either 15–50 kb or 20–50 kb BluePippin size selection (Sage Science, Beverly, MA, USA) was performed on each library. Recovered fragments were sequenced using P6C4 sequencing chemistry on the RS II platform (240 min movie time). The four SMRTbell libraries were sequenced on 97 SMRT cells to generate 7,745,233 subreads for 76.6 Gb of raw data. Collectively, short- and long-read sequencing produced around 359.5 Gb of data (Table 1).

### Genome assembly

We employed a hybrid *de novo* whole-genome assembly strategy, combining both short-read and long-read data. Trimmed Q30-filtered short reads were *de novo* assembled with ABySS v1.3.6 [21] using k = 64 and default parameters (contig N50 = 583 bp) (Table 2). Long sequence reads were *de novo* assembled using the program DBG2OLC [22] (k 17 AdaptiveTh 0.0001 KmerCovTh 2 MinOverlap 20 RemoveChimera 1) (contig N50 = 167.04 kbp) (Table 2). Following this, both assemblies were merged together using the hybrid assembler (“sparc”) tool of DBG2OLC with default parameters, combining the contiguity of the long-read data with the improved accuracy of the high-coverage Illumina assembly. This hybrid assembly (v2.0) was twice “polished” to remove errors. In the first round, the Q30-trimmed Illumina reads were mapped to the hybrid assembly with bowtie v2.2.9 [23] and filtered for proper pairs using samtools v1.3.1 [24]. Scaffolds were polished with Pilon v1.21 [25] to generate the second iteration of the assembled genome (v2.1). In the second round, PacBio subreads were mapped to assembly v2.1 for error correction using SMRT analysis software (PacBio). PacBio subreads for each library were converted to BAM format with bax2bam v0.0.08 and aligned to the genome using pbalign v.0.3.0. BAM alignment files were combined using samtools merge v1.3.1, and the scaffolds were polished with Arrow v2.1.0 to generate the final genome assembly (v2.2). Our final draft assembly of the cane toad genome (v2.2) has 31-392 scaffolds with an N50 of 167 kb (Table 2). The GC content (43.23%) is within 1% of the published estimate of 44.17%, determined by flow cytometry [26].

**Table 2:** Summary of genome assemblies For comparison, statistics are provided for two existing neobatrachian genomes, *Nanorana parkeri* (v2) [15] and *Lithobates catesbeianus* (v2.1)[14], and two anuran reference genomes, *Xenopus tropicalis* (v9.1) [16] and *Xenopus laevis* (v9.2) [17]. Lengths are given to 3 significant figures (s.f.). All percentages are given to 1 decimal point (d.p).

Genome assembly	Hybrid (v2.2)	Short read	Long read	*N. parkeri* (v2.0)	*L. catesbeianus* (v2.1)	*X. tropicalis*(v9.1)	*X. laevis* (v9.2)
Total length (Gb)	2.55	3.75	2.69	2.07	6.25	1.44	2.72
No. scaffolds	31,392	19.9 M^a^	31,392^a^	135,808	1.54 M	6,822	108,033
Proportion gap (%N)	0.0	0.1	0.0	3.9	11.6	4.9	11.4
N50	168 kb	583 bp	167 kb	1.06 Mb	39.4 kb	135 Mb	137 Mb
L50	3,373	715 k	3,531	555	31,248	5	9
Longest scaffold	3.53 Mb	72.6 kb	3.64 Mb	8.61 Mb	1.38 Mb	195 Mb	220 Mb
GC (%)	43.2	43.3	42.9	42.6	43.1	40.1	39.0
BUSCO^b^							
Complete single copy (%)	80.9	15.5	2.2	83.4	42.3	87.5	52.9
Complete duplicate (%)	2.2	0.7	0.0	1.6	0.9	1.0	39.8
Fragment (%)	7.5	33.6	2.2	7.2	22.3	6.0	3.2

For comparison, statistics are provided for two existing neobatrachian genomes, Nanorana parkeri (v2) [15] and Lithobates catesbeianus (v2.1)[14], and two anuran reference genomes, Xenopus tropicalis (v9.1) [16] and Xenopus laevis (v9.2) [17]. Lengths are given to 3 significant figures (s.f.). All percentages are given to 1 d.p.

a Statistics for short- and long-read assemblies refer to contigs used for hybrid assembly.

b Benchmarking Universal Single-Copy Orthologs v2.0.1 short summary statistics (n = 3,950).

### Assessment of genome completeness

**Figure 3:**
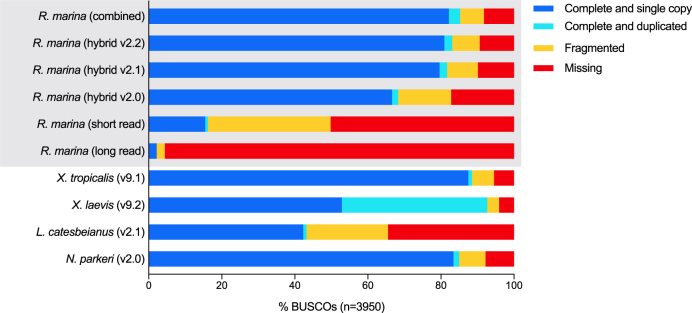
Assessment of genome assembly completeness. BUSCO analysis of *Rhinella marina* genome assembly (v2.0 uncorrected, v2.1 pilon polishing, v2.2 pilon and arrow polishing, combined v2.1, 2.2, and 2.2 ratings), *Lithobates catesbeianus* (v2.1), *Nanorana parkeri* (v2.0), *Xenopus tropicalis* (v9.1), and *Xenopus leavis* (v9.2) genomes using the tetrapoda_odb9 orthologue set (n = 3,950). The *Xenopus leavis* genome duplication is made clear by the large number of paralogs (light blue) with respect to other assemblies.

BUSCO [27] analysis of conserved single-copy orthologues is widely used as a proxy for genome completeness and accuracy. While direct comparisons are only truly valid within an organism, comparing BUSCO scores to genomes from related organisms provides a useful benchmark. We ran BUSCO v2.0.1 (short mode, lineage tetrapoda_odb9, BLAST+ v2.2.31 [28], HMMer v3.1b2 [29], AUGUSTUS v3.2.2 [30], EMBOSS v6.5.7 [31] on each of our assemblies, along with four published anuran genomes (Fig. 3, Table 2). The hybrid assembly combined the completeness of the long-read assembly with the accuracy of the short-read assembly, providing an enormous boost in BUSCO completeness from less than 50% full and partial orthologs to more than 90%. Error correction through pilon and arrow polishing had a positive effect on the BUSCO measurement of genome completeness, with an increase of 7.8% in the number of full and partial orthologs between v2.0 and v2.2. For the polished assembly (v2.2), 3,279 (83.0%) of the 3,950 ultraconserved tetrapod genes were complete, 296 (7.5%) were fragmentary, and 375 (9.5%) were missing. It should be noted that these numbers mask some underlying complexity of BUSCO assessments; aggregate improvements in BUSCO scores with polishing include some losses as well as gains. Taking the best rating for each BUSCO in v2.0, v2.1, or v2.2 reduces the number of missing BUSCO genes to 326 (8.3%) and increases the complete number to 3,366 (85.2%) (Fig. 3, “*R. marina* (combined)”). This is explored further in the “Genome annotation and prediction” section below. Overall, BUSCO metrics indicate that our draft *R. marina* genome is approaching the quality and completeness of the widely used anuran amphibian reference genomes for *X. laevis* (v9.2) [17] and *X. tropicalis* (v.9.1) [16] and compares well to the recently published neobatrachian genomes of *Nanorana parkeri* (v2) [15] and *Lithobates catesbeianus* (v2.1) [14].

### Estimation of *R. marina* genome size

**Figure 4:**
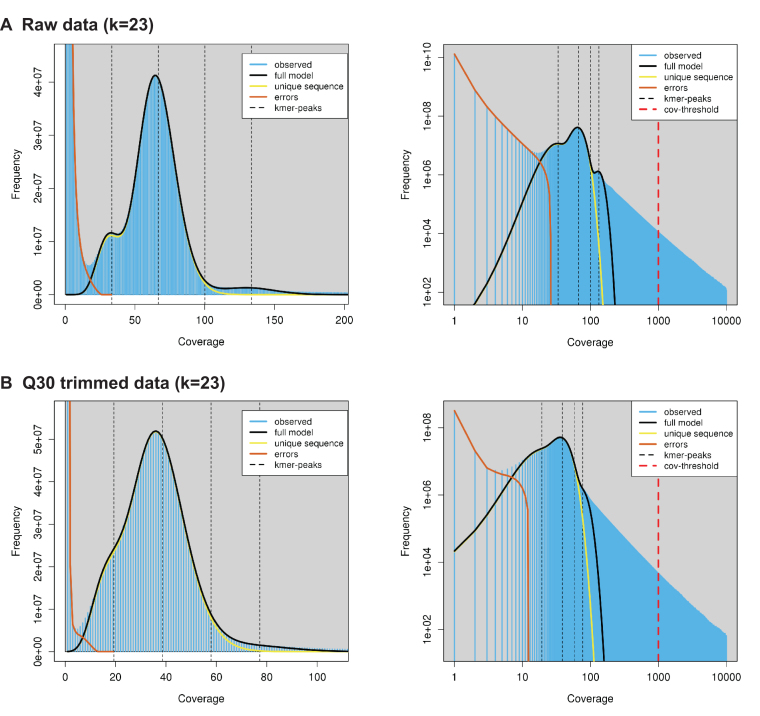
GenomeScope *k*-mer frequency and log-transformed *k*-mer coverage profiles. **(A)** Raw Illumina data (*k* = 23). **(B)** Q30 trimmed Illumina data (*k* = 23). Profiles for *k* = 21 are similar (data not shown).

Previous reports have estimated the size of the cane toad genome at being from 3.98 to 5.65 Gb using either densitometry or flow cytometry analysis of stained nuclei within erythrocytes, hepatocytes, and renal cells [26, 32-38]. We employed two alternative strategies to measure the genome size, using short-read *k*-mer distributions and quantitative PCR (qPCR) of single copy genes. The *k*-mer frequencies were calculated for both raw and trimmed Q30-filtered paired-end short reads (Table 1) with Jellyfish v2.2.3 [39] using *k* = 21 and *k* = 23 and a maximum k-mer count of 10,000. The *k*-mer distributions were analyzed using GenomeScope [40] with mean read lengths of 148 bp (raw) or 141 bp (Q30) and k-mer coverage cutoffs of 1,000 and 10,000 (Table 3, Fig. 4). GenomeScope gave genome size estimates ranging from 1.77 Gb to 2.30 Gb, with the raw reads giving consistently larger estimates (1.85 Gb to 2.30 Gb) than the trimmed and filtered reads (1.77 Gb to 2.10 Gb). Estimates of the unique (single-copy) region of the genome were more consistent, ranging from 1.31 Gb to 1.46 Gb, with *k* = 23 estimates 99 Mb (raw) or 80 Mb (Q30) higher than *k* = 21. Increasing the GenomeScope maximum *k*-mer coverage threshold had the greatest effect on predicted genome size, increasing repeat length estimates by 274 Mb to 385 Mb. GenomeScope explicitly models heterozygous diploid *k-*mer distributions, which should make it robust to the additional challenge of sequencing a wild animal. However, GenomeScope predictions are affected by nonuniform repeat distributions, and this difference could indicate high copy number repeats in the genome that are difficult to model accurately. It is possible that high-frequency repeats with raw sequencing counts exceeding 10,000 are resulting in an underestimate of total repeat length and therefore genome size compared to the previous densitometry and flow cytometry predictions.

**Table 3:** GenomeScope genome size estimates for *Rhinella marina* based on raw trimmed Illumina data using different combinations of *k* and maximum *k*-mer coverage .

		Unique length (Mb)		Repeat length (Mb)		Genome size (Mb)	
Data	Max *k-*mer coverage	Min	Max	Min	Max	Min	Max
Raw (*k* = 21)	1,000	1,365	1,366	489	489	1,853	1,855
Raw (*k* = 21)	10,000	1,365	1,365	874	874	2,239	2,240
Raw (*k* = 23)	1,000	1,453	1,455	470	471	1,924	1,926
Raw (*k* = 23)	10,000	1,454	1,454	842	842	2,296	2,296
Q30 (*k* = 21)	1,000	1,307	1,308	462	462	1,768	1,771
Q30 (*k* = 21)	10,000	1,307	1,308	749	749	2,056	2,057
Q30 (*k* = 23)	1,000	1,389	1,391	438	439	1,828	1,830
Q30 (*k* = 23)	10,000	1,390	1,391	713	713	2,103	2,104

Lengths are in megabases (0 d.p.).

In the second approach, the *zfp292* (zinc finger protein 292) gene was selected from our BUSCO analysis as a single-copy target for genome estimation by qPCR [41]. First, PCR was used to amplify a 326-bp region of *zfp292* (scaffold 6589, position 345 750–346 075) in a 25 µL reaction that contained 50 ng of gDNA, 200 µM dNTP, 0.625 units of Taq polymerase (Invitrogen), 10 × Taq polymerase buffer (Invitrogen), and 0.4 µM of each primer (Supplementary Table S1). The amplicon was cloned into the pGEM-T Easy vector (Promega, Madison, WI, USA), and the resultant plasmid was linearized with NdeI before being serially diluted to generate a qPCR standard (10^1^–10^9^ copies/µL). To amplify a smaller region (120 bp) within *zfp292* (scaffold 6589, position 345 858–345 977), gDNA (10–25 ng) or 1 µL of the diluted standards was used as a template for a 20 µL qPCR reaction containing 2 × iTaq SYBR Green mastermix (BioRad, Hercules, CA, USA) and 0.5 µM of each primer (Supplementary Table S1). Cycle threshold values obtained for each plasmid dilution were used to generate a standard curve and infer the number of *zfp292* amplicons generated from the template gDNA of known quantity. Genome sizes were generated from the formulae outlined by [41], and the average of two estimates (2.81 Gb and 1.94 Gb) was used to obtain a genome size of 2.38 Gb. This genome size provides an estimated combined 151X sequencing coverage (119X Illumina and 32X PacBio) (Table 4).

**Table 4:** Estimation of *Rhinella marina* genome size using various methods and the corresponding level of sequencing coverage (3 s.f.)

Method	Estimated genome size (Gb)	Illumina coverage (X)	PacBio coverage (X)	Reference
Flow cytometry (mean)	4.33	65.3	17.7	[26, 33, 35, 38]
Flow cytometry (min)	3.98	71.1	19.2	[38]
Flow cytometry (max)	4.90	57.7	15.6	[35]
Densitometry (mean)	4.95	57.1	15.5	[32, 34, 36, 37]
Densitometry (min)	4.06^a^	69.7	18.9	[37]
Densitometry (max)	5.65	50.1	13.6	[32]
GenomeScope (raw)	2.08	136	36.8	-
GenomeScope (Q30)	1.94	146	39.4	-
qPCR (*zfp292*)	2.38	119	32.1	-
Assembly (v2.2)	2.55	111	30.0	-

GenomeScope values in this table are mean values from the four setting combinations.

^a^Value adjusted to account for updated size of reference genome used to infer *R. marina* genome size.

Our genome size estimation of 1.98 to 2.38 Gbp is smaller than the 2.55 Gbp assembly size and differs significantly from previously published estimates of 4 Gbp or more for this species. We suggest that this is a result of the repetitive nature of the genome (see below). Given this is the first estimate of the cane toad genome size using either *k*-mer or qPCR analysis, further investigations are required to more clearly understand the discrepancy in our estimates with respect to published genome sizes. Here, we estimate the depth of sequencing coverage using both sequence-based and cytometric genome size measures (Table 4).

### Genome annotation and gene prediction

Annotation of the draft genome was performed using MAKER2 v2.31.6 [42], BLAST+ v2.2.31 [28], AUGUSTUS v3.2.2 [30], Exonerate v2.2.0 [43], RepeatMasker v4.0.6 [44] (DFAM [45], Library Dfam_1.2; RMLibrary v20150807), RepeatModeler v1.0.8 [46], and SNAP v2013-11-29 [47] using all Swiss-Prot protein sequences (downloaded 3 February 2017) [48]. AUGUSTUS was trained using BUSCO v2.0.1 (long mode, lineage tetrapoda_odb9) and a multitissue reference transcriptome we previously generated from tadpoles and six adult cane toads [18] (available from GigaDB [49], GenBank accession PRJNA383966). Whole tadpoles and the brain, liver, spleen, muscle, ovary, and testes of adult toads from Australia and Brazil were used to prepare cDNA libraries for the multitissue transcriptome sequencing. After the initial training run, two additional iterations of MAKER2 were run using hidden Markov models (HMMs) from SNAP training created from the previous run. Functional annotation of protein-coding genes predicted by MAKER2 were generated using Interproscan 5.25–64.0, with the following settings: -dp -t p -pa -goterms -iprlookup -appl TIGRFAM, SFLD, Phobius, SUPERFAMILY, PANTHER, Gene3D, Hamap, ProSiteProfiles, Coils, SMART, CDD, PRINTS, ProSitePatterns, SignalP_EUK, Pfam, ProDom, MobiDBLite, PIRSF, TMHMM. BLAST+ v2.6.0 [28] was used to annotate predicted genes using all Swiss-Prot proteins (release 2017_08, downloaded 2017-09-01) [48] using the following settings: -evalue 0.000001 -seg yes -soft_masking true -lcase_masking -max_hsps 1.

**Figure 5:**
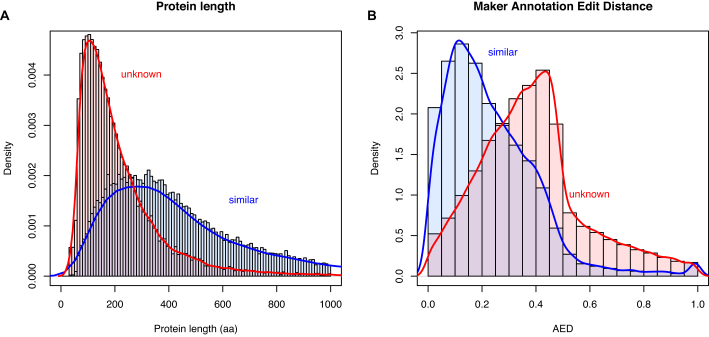
Key protein statistics for predicted genes with and without annotated similarity to known genes. Histograms of **(A)** protein length and **(B)** MAKER2 AED for “similar” (blue) and “unknown” (red) classes of predicted genes.

**Figure 6:**
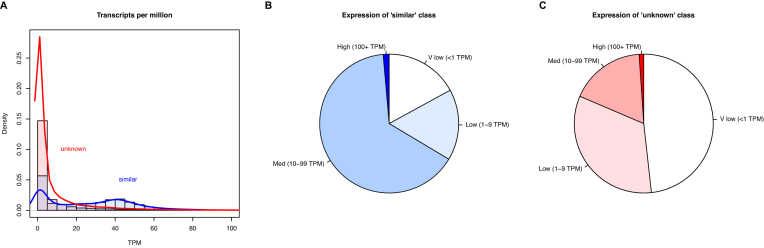
Multitissue gene expression for predicted genes with and without annotated similarity to known genes. **(A)** Histograms of RNA-seq TPM for “similar” (blue) and “unknown” (red) classes of predicted genes, capped at 100 TPM. **(B)** “Similar” and (C) “unknown” gene expression, rated as very low (<1 TPM), low (1–9 TPM), medium (10–99 TPM), or high (100+ TPM).

In total, 58,302 protein-coding genes were predicted by the MAKER pipeline, with an average of 5.3 exons and 4.3 introns per gene (Table 5). Of these, 5,225 are single-exon genes, giving 4.7 introns per multi-exon gene with an average intron length of 4.08 kb. Predicted coding sequences make up 2.38% of the assembly. MAKER predicted considerably more than the approximately 20,000 genes expected for a typical vertebrate genome. There are two likely explanations for this: artifactual duplications in the genome assembly, either through underassembly or legitimate assembly of two heterozygous diploid copies; or the overprediction of proteins during genome annotation, including pseudogenes with high homology to functional genes, proteins from transposable elements or other repeats, and multiple fragments of open reading frames (ORFs) from the same gene (due to fragmentation of the genome) and lncRNA genes that have been incorrectly assigned a coding sequence. Of the 3,279 complete BUSCO genes identified (Table 2), only 85 (2.59%) were duplicated. This suggests that there is not widespread duplication in the assembly. Only 25,846 predicted genes were annotated as similar to known proteins in Swiss-Prot, with the remaining 32,456 predictions “of unknown function.” This is consistent with overprediction being the primary cause of inflated gene numbers. Poor-quality protein predictions are generally shorter (generated from fragmented or random ORFs) and have a larger annotation edit distance (AED) when compared to real proteins. Consistent with this, the predicted proteins of unknown function are shorter in sequence (median length 171 aa) compared to those with Swiss-Prot hits (median length 388 aa) (Fig. 5A) and have a greater AED (median 0.37 vs 0.2) (Fig. 5B). To investigate this further, predicted transcript and protein sequences were searched against the published *de novo* assembled transcriptome [18] using BLAST+ v2.2.31 [28] blastn or tblastn (top 10 hits, e-value <10^−10^) and compiled with GABLAM v2.28.3 [50]. For 56.5% of proteins with functional annotation, 95%+ of the protein length mapped to the top transcript hit (Table 6). Only 27.1% of unknown proteins had 95%+ coverage in the top transcript hit, which is again consistent with overprediction. We also reanalyzed the multitissue RNA sequencing (RNA-seq) data from Richardson et al. [18] by mapping the reads onto the MAKER predicted transcripts. Filtered reads (adaptor sequences and reads with average Phred <30 removed) were mapped with Salmon v0.8.0 [51] (Quasi-mapping default settings, IU libtype parameter). Read counts were converted into transcripts per million (TPM) by normalizing by transcript length, dividing by the sum of the length-normalized read counts, and then multiplying by 1 million. We observed lower expression levels overall in the “unknown” set (Fig. 6). With the caveat that real proteins may have very low expression, this is also consistent with the “unknown” gene set containing false annotations. Further review of the predicted protein descriptions revealed 4,357 with likely origins in transposable elements (including 4,114 long interspersed nuclear element-1 [LINE-1] ORFs) and 215 from viruses. However, many of these may be *bona fide* functional members of the cane toad proteome: 1,447 (33.2%) “transposon” and 151 (70.2%) of “viral” transcripts had support for expression >1 TPM.

**Table 5:** Summary statistics of consensus protein-coding gene predictions and predicted repeat elements (including RNA genes) for the *Rhinella marina* v2.2 draft genome.

Element	Count	No. of scaffolds	Avg. length	Total length	Genome coverage (%)	PacBio depth (X)	Illumina depth (X)
Protein-coding gene	58,302	19,530	18.8 kb	1.10 Gb	42.91	20.32	58.07
Transcript	58,302	19,530	1.24 kb	72.3 Mb	2.83	20.49	65.41
- Similar to known	25,846	11,918	1.90 kb	49.1 Mb	1.92	20.08	56.42
- Unknown	32,456	15,213	714 bp	23.2 Mb	0.91	20.98	68.82
Exon	309,718	19,530	233 bp	72.3 Mb	2.83	20.49	65.41
- Coding	294,535	19,530	207 bp	60.8 Mb	2.38	20.67	66.97
Intron	251,416	18,509	4.08 kb	1.03 Gb	40.09	20.30	57.55
5’ untranslated region	15,855	8,839	208 bp	3.29 Mb	0.13	18.69	53.86
Coding sequence	58,302	19,530	1.04 kb	60.8 Mb	2.38	20.67	66.97
3’ untranslated region	11,965	5,780	682 bp	8.16 Mb	0.32	19.91	58.52
BUSCO SC complete	3,194	2,014	32.6 kb	104 Mb	4.07	19.89	53.01
**Repeats**							
Short interspersed nuclear element	21,620	9,322	338 bp	7.31 Mb	0.29	19.45	58.23
Long interspersed nuclear element	268,569	27,620	513 bp	138 Mb	5.38	21.03	72.29
Long terminal repeat	201,817	24,949	504 bp	102 Mb	3.98	22.62	68.96
DNA	817,405	30,689	600 bp	490 Mb	19.17	21.67	68.37
Helitron	20,319	9,340	826 bp	16.8 Mb	0.66	19.32	56.81
Retroposon	1,042	829	549 bp	570 kb	0.02	18.22	50.87
Other	18	17	209 bp	3.7 kb	0.00%	14.27	24.60
Unknown	1,610,883	30,966	513 bp	826 Mb	32.28	20.12	59.39
Satellite	25,557	10,270	440 bp	11.3 Mb	0.44	18.38	54.21
Simple repeats	968,947	30,620	56.9 bp	55.1 Mb	2.16	18.88	48.51
Low complexity	141,028	24,020	51.8 bp	7.30 Mb	0.29	22.48	64.48
rRNA	5,227	2,923	422 bp	2.20 Mb	0.09	40.88	142.42
tRNA	5,558	4,474	105 bp	583 kb	0.02	29.15	140.06
snRNA	21,788	9,432	546 bp	11.9 Mb	0.47	24.63	89.12
srpRNA	17	11	268 bp	4.55 kb	0.00	22.11	140.44
scRNA	3	3	69.0 bp	207 bp	0.00	15.53	47.29
RNA	418	266	482 bp	202 kb	0.01	32.65	173.99
**Repeat TOTAL^a^**	4,110,222	31,179	406 bp	1.63 Gb	63.9	20.82	63.79

Lengths are given to 3 s.f. Coverage and mean depth statistics for PacBio and Q30-trimmed Illumina reads are given to 2 d.p. LINE: long interspersed nuclear element

a Values for repeat totals account for overlapping repeats.

**Table 6:** Proportions of predicted protein and transcript sequences exceeding 50%, 80%, 95%, or 99% coverage in the top BLAST+ hit from the published transcriptome [18], and combined coverage for the top 10 transcript hits.

Type	Count	Coverage in top transcript hit				Coverage in top 10 transcript hits			
		50%+	80%+	95%+	99%+	50%+	80%+	95%+	99%+
Protein (similar to known)	25,846	93.6	76.7	56.5	40.7	97.5	90.3	72.7	54.2
Transcript (similar to known)	25,846	75.0	50.0	30.8	21.4	82.6	73.1	57.2	40.9
Protein (unknown)	32,456	79.9	49.8	27.1	15.8	85.7	66.3	44.4	29.9
Transcript (unknown)	32,456	43.6	21.5	12.1	8.61	52.6	37.3	25.4	19.1

All percentages given to 3 s.f.

To investigate the role of fragmented ORFs, we downloaded the Quest For Orthologues (QFO) reference proteomes (QFO 04/18) [52] and used BLAST+ v2.2.31 [28] blastp (e-value <10^−7^) to identify the top hit for each predicted protein in all eukaryote reference proteomes and in the *X. tropicalis* reference proteome. BLAST results were converted into global coverage with GABLAM v2.28.3 [50]. As expected, the vast majority (99.6%) of “similar” proteins had a blastp hit the QFO proteomes (data not shown). Perhaps surprisingly, nearly two thirds (66.5%) of “unknown” proteins also had a blastp hit, but these had lower coverage of the reference proteins than did proteins in the “similar” class (data not shown). A “combined coverage” score was calculated for each protein, taking the minimum percentage coverage of either the query protein or its top QFO hit. This metric was related to annotation quality, showing an inverse relationship with AED (data not shown). Excluding proteins with annotation indicating possible viral or transposable element origin, 45.7% of “similar” proteins and 96.8% of “unknown” proteins had the same closest *X. tropicalis* blastp hit as another predicted protein. Consistent with this being related to gene fragmentation, there was a negative relationship between the number of cane toad proteins sharing a given *X. tropicalis* top hit and how much of the *X. tropicalis* hit was covered by each cane toad protein. Nevertheless, it is likely that some of these protein fragments represent allelic variants that have been redundantly assembled.

**Figure 7:**
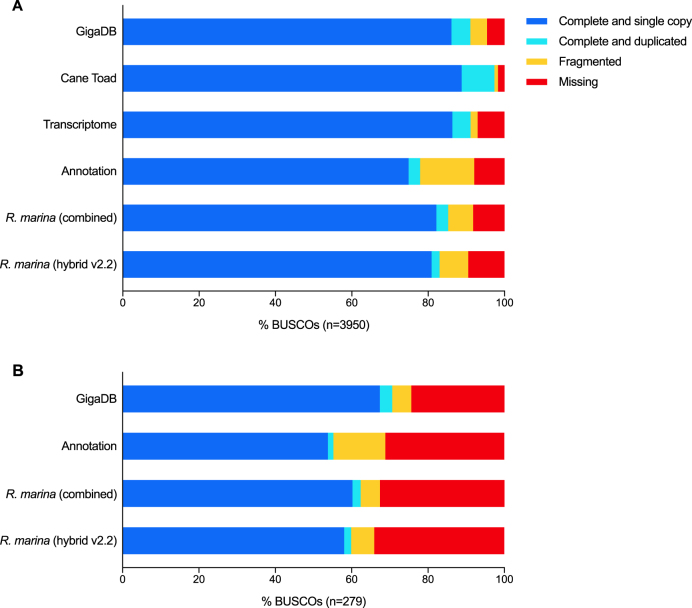
Assessment of assembly annotation completeness. BUSCO analysis for **(A)** all BUSCO tetrapoda genes (n = 3950) and **(B)** the subset of BUSCO genes rated as “Missing” from the Richardson et al. transcriptome [18]. *Rhinella marina* (combined): combined v2.0, v2.1, and v2.2 ratings; Annotation: combined MAKER proteome and transcriptome ratings; GigaDB: combined assembly and annotation ratings; Cane Toad: combined assembly, annotation, and Richardson et al. transcriptome [18].

We ran BUSCO v2.0.1 (short mode, lineage tetrapoda_odb9, BLAST+ v2.2.31 [28], HMMer v3.1b2 [29], AUGUSTUS v3.2.2 [30], EMBOSS v6.5.7 [31]) on the MAKER2 transcriptome and proteome and retained the most complete rating for each gene (Fig. 7A, Supplementary Table S2, “Annotation”). MAKER annotation had fewer missing BUSCO genes than the v2.2 assembly (314 vs 375) but many more fragmented (561 vs 296). Equivalent BUSCO analysis of the Richardson et al. transcriptome [18] was only missing 296 genes. However, as seen with the assembly versions, these values mask hidden complexity. Combined BUSCO analysis of our hybrid assembly (v2.0, v2.1, v2.2) and annotation, revealed only 181 missing genes (Fig. 7A, Supplementary Table S2, “GigaDB”). Furthermore, >50% of the 279 genes “Missing” in the transcriptome are found in the genome and/or its annotation (Fig. 7B, Supplementary Table S2). When the transcriptome and our genome are combined, only 68 BUSCO genes (1.7%) are “Missing” and 3,845 (97.3%) are “Complete” (Fig. 7A, Supplementary Table S2, “CaneToad”). This highlights the usefulness of our assembly and illustrates the complementary nature of genome and transcriptome data. The former is more comprehensive but more difficult to assemble and annotate, whereas the latter is easier to assemble into full-length coding sequences but will miss some tissue-specific and lowly expressed genes. Some of the remaining “Missing” BUSCO genes may be present but too fragmented to reach the score threshold.

Future work is needed to improve the quality of gene annotation. We have included all of the MAKER2 predictions in our annotation as well as a full table of protein statistics and top blastp hits from this analysis for further biological analyses (Supplementary Table S3). Annotation has also been made available via a WebApollo [53] genome browser [54] and an associated search tool [55]. This will facilitate community curation and annotation of genes of interest. For researchers who would like to use cane toad proteins in general evolutionary analyses, we have also created a “high-quality” dataset of 6,580 protein-coding genes with an AED no greater than 0.25 and at least 90% reciprocal coverage of its top QFO blastp hit, excluding possible viral and transposon proteins, available from the *GigaScience* database.

### Phylogenetic analysis of high-quality proteins

**Figure 8:**
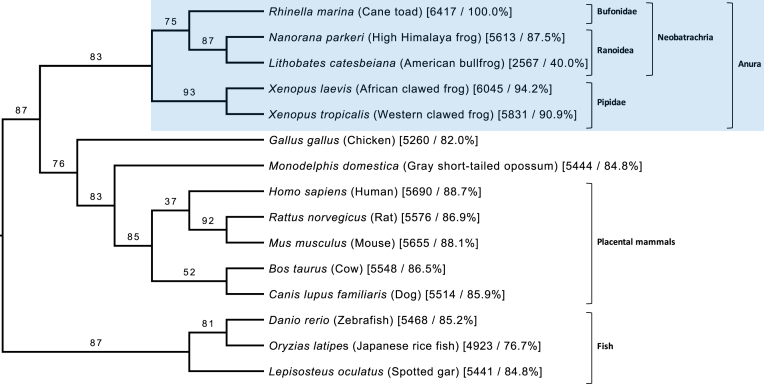
Phylogenetic supertree of 15 selected chordate taxa constructed from phylogenetic trees for 6,417 high-confidence cane toad proteins. Branch labels indicate percentage consistency (see text), rounded down. Numbers following each taxon are the number and percentage of source trees containing that taxon. The tree has been rooted using fish as an outgroup and visualized with FigTree [60]. The full supertree of 52 taxa is available as Supplementary Fig. S1.Pl

To further validate the high-quality protein dataset, GOPHER [56] v3.4.2 was used to predict orthologues for each protein. QFO (04/18) [52] eukaryotic reference proteomes were supplemented with Uniprot Reference proteomes for *Lithobates catesbeiana* (UP000228934) [14] and *Xenopus laevis* (UP000186698) [17] and the annotated protein sequences of *Nanorana parkeri* v2 [15]. GOPHER orthologues were predicted with default settings based on a modified mutual best-hit algorithm that accounts for one-to-many or many-to-many orthologous relationships and retains the closest orthologue from each species. The closest orthologues were aligned with MAFFT [57] v7.310 (default settings) and phylogenetic trees inferred with IQ-TREE [58] v1.6.1 (default settings) for alignments containing at least three sequences. Phylogenetic trees were inferred in this manner for 6,417 of the 6,580 high-quality proteins. A supertree was then constructed from the 6,417 individual protein trees using CLANN [59] v4.2.2 (DFIT Most Similar Supertree Algorithm) (Fig. 8, Supplementary Fig. S1). Branch consistency was calculated for each branch as the proportion of source trees with taxa on either side of the branch that have no conflicts in terms of the placement of those taxa. The supertree supports the known phylogeny for amphibians used in this study, giving additional confidence in the quality and utility of these protein annotations. All alignments and trees are available in Supplementary Data via the *GigaScience* database.

### Repeat identification and analysis

**Figure 9:**
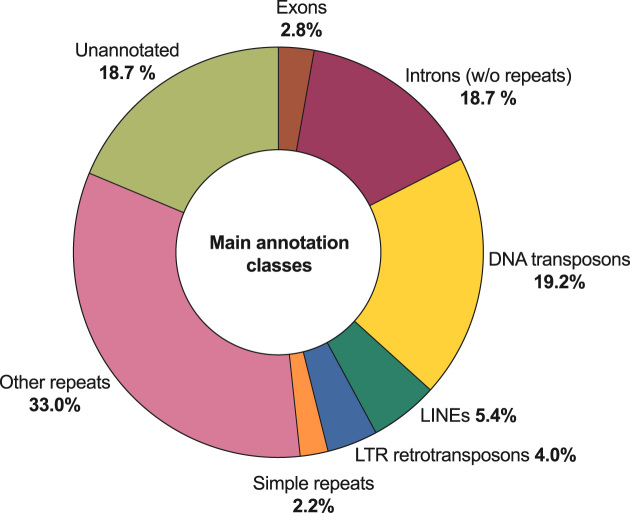
Summary of the main annotation classes for *Rhinella marina* genome assembly. Identified repeat classes exceeding 2% of assembly have been plotted separately (1 d.p.). All other repeats, including “Unknown,” have been grouped as “Other repeats.” The percentage for introns excludes any repeat sequences within those introns.

The cane toad genome has proven very difficult to assemble using short reads alone, which suggests a high frequency of repetitive sequences, as for other amphibians [12, 13]. RepeatMasker annotations from the MAKER pipeline support this interpretation, with more than 4.1 million repeat sequences detected, accounting for 63.9% of the assembly (Table 5). The mean repeat length is 406 bp, which exceeds the Illumina read length used in our study (mean 140.6 bp paired-end). This makes short-read assembly of these regions difficult, as reflected by the poor ABySS contiguity (contig N50 = 583 bp; Table 2), and emphasizes the need for long-read data in this organism. The most abundant class of repeat elements is of unknown type (1.61 million elements covering 32.28% of the assembly), with DNA transposons the most abundant known class of element (817,262 repeats; 19.17% coverage). Of these, the most abundant are of the hAT-Ac (231,332 copies) and TcMar-Tc1 (226,145copies) superfamilies (Supplementary Table S4). Accounting for overlaps between repeat and gene features, 18.7% of the assembly (479,397,014 bp) has no annotation (Fig. 9).

## Conclusion

This draft genome assembly will be an invaluable tool for advancing knowledge of anuran biology, genetics, and the evolution of invasive species. Furthermore, we envisage these data will facilitate the development of biocontrol strategies that reduce the impact of cane toads on native fauna.

## Supplementary Material

GIGA-D-18-00104_Original_submission.pdf

GIGA-D-18-00104_Revision_2.pdf

GIGA-D-18-00104_Revision_3.pdf

Response_to_Reviewer_Comments_Original_Submission.pdf

Response_to_Reviewer_Comments_Revision_2.pdf

Reviewer_1_Report_(Original_Submission) -- Rene L Warren 4/9/2018 Reviewed

Reviewer_1_Report_(Revision_2) -- Rene L Warren 7/3/2018 Reviewed

Reviewer_2_Report_(Original_Submission) -- Taejoon Kwon 4/15/2018 Reviewed

Reviewer_2_Report_(Revision_2) -- Taejoon Kwon 7/9/2018 Reviewed

Reviewer_3_Report_(Original_Submission) -- Masanori Taira 4/24/2018 Reviewed

Reviewer_3_Report_(Revision_2) -- Masanori Taira 7/15/2018 Reviewed

Reviewer_4_Report_(Original_Submission) -- John Malone 4/24/2018 Reviewed

Additional Files

## Availability of supporting data

Raw genomic sequencing data (Illumina and PacBio) and assembled scaffolds have been deposited in the European Nucleotide Archive with the study accession PRJEB24695 and assembly accession GCA_900 303 285. The genome assembly and annotation are also available in the *GigaScience* database and via a WebApollo [53] genome browser and an associated search tool [55]. Data further supporting this work are available in the *GigaScience* database [61].

## Additional files

**Figure S1**. Phylogenetic supertree constructed from phylogenetic trees for 6417 high confidence cane toad proteins.

**Table S1**. Primers used for genome size estimation by single copy gene qPCR.

**Table S2**. Individual and combined full BUSCO gene ratings for cane toad assemblies, annotation, transcriptome.

**Table S3**. Sequence statistics, top BLAST hits, and classification for MAKER2 annotations.

**Table S4**. RepeatMasker statistics broken down by repeat category.

## Abbreviations

AED: annotation edit distance; BLAST: Basic Local Alignment Search Tool; BUSCO: Benchmarking Universal Single-Copy Orthologs; d.p.: decimal point; gDNA: genomic DNA; HMM: hidden Markov model; LINE: long interspersed nuclear element; ORF: open reading frame; PacBio: Pacific Biosciences; PCR: polymerase chain reaction; QFO: Quest For Orthologues; qPCR: quantitative polymerase chain reaction; RNA-seq: RNA sequencing; s.f.: significant figure; SMRT: single-molecule real time; TPM: transcripts per million; zfp292: zinc finger protein 292; ZMW: zero-mode waveguide.
