# Supplementary material for: Draft genome assembly of the invasive cane toad, Rhinella marina
Source: Gigascience. 2018 Aug 7;7(9):giy095. doi: 10.1093/gigascience/giy095 (PMC6145236; doi:10.1093/gigascience/giy095)
Supplement: GIGA-D-18-00104_Original_submission.pdf [file giy095_giga-d-18-00104_original_submission.pdf]

GigaScience

Draft genome assembly of the invasive cane toad, Rhinella marina

--Manuscript Draft--

**Manuscript Number:**	GIGA-D-18-00104
**Full Title:**	Draft genome assembly of the invasive cane toad, Rhinella marina
**Article Type:**	Data Note
**Funding Information:**	Australian Research Council (DP160102991) — Dr. Lee A Rollins, Prof. Richard Shine<br>Australian Research Council (DE150101393) — Dr. Lee A Rollins<br>Australian Research Council (FL170100022) — Prof. Edward C Holmes<br>Australian Research Council (LE150100031) — Prof. Marc R Wilkins<br>Australian Research Council (FL120100074) — Prof. Richard Shine<br>The National Council for Scientific and Technological Development (CNPq) (302892/2016-8) — Assoc. Prof. Marcelo Vallinoto<br>Bioplatforms Australia — Not applicable
**Abstract:**	Background: The cane toad (Rhinella marina) is a species native to Central and South America that has spread across many regions of the globe. Cane toads are known for their rapid adaptation and deleterious impacts on native fauna in invaded regions. However, despite an iconic status, there are major gaps in our understanding of cane toad genetics. The availability of a genome would help to close these gaps and accelerate cane toad research. Findings: We report a draft genome assembly for R. marina, the first of its kind for the Bufonidae family. We used a combination of long read PacBio RS II and short read Illumina HiSeq X sequencing to generate a total of 359.5 Gb of raw sequence data. The final hybrid assembly of 31,392 scaffolds was 2.55 Gb in length with a scaffold N50 of 168 kb. BUSCO analysis revealed that the assembly included full length or partial fragments of 90.6% of tetrapod universal single-copy orthologs (n=3950), illustrating that the gene-containing regions have been well-assembled. Annotation predicted 58,302 protein coding genes, with 25,846 similar to known proteins in SwissProt. Repeat sequences were estimated to account for 63.9% of the assembly. Conclusion: The R. marina draft genome assembly will be an invaluable resource that can be used to further probe the biology of this invasive species. Future analysis of the genome will provide insights into cane toad evolution and enrich our understanding of their interplay with the ecosystem at large.
**Corresponding Author:**	Peter White, Ph.D.<br>University of New South Wales<br>Sydney, NSW AUSTRALIA
**Corresponding Author Secondary Information:**	
**Corresponding Author's Institution:**	University of New South Wales
**Corresponding Author's Secondary Institution:**	
**First Author:**	Richard J Edwards
**First Author Secondary Information:**	
**Order of Authors:**	Richard J Edwards<br>Daniel Enosi Tuipulotu<br>Timothy G Amos<br>Denis O'Meally

Powered by Editorial Manager® and ProduXion Manager® from Aries Systems Corporation

	Mark F Richardson
	Tonia L Russell
	Marcelo Vallinoto
	Miguel Carneiro
	Nuno Ferrand
	Marc R Wilkins
	Fernando Sequeira
	Lee A Rollins
	Edward C Holmes
	Richard Shine
	Peter A White
**Order of Authors Secondary Information:**	
**Additional Information:**	
**Question**	**Response**
Are you submitting this manuscript to a special series or article collection?	No
**Experimental design and statistics**<br><br>Full details of the experimental design and statistical methods used should be given in the Methods section, as detailed in our Minimum Standards Reporting Checklist. Information essential to interpreting the data presented should be made available in the figure legends.<br><br>Have you included all the information requested in your manuscript?	Yes
**Resources**<br><br>A description of all resources used, including antibodies, cell lines, animals and software tools, with enough information to allow them to be uniquely identified, should be included in the Methods section. Authors are strongly encouraged to cite Research Resource Identifiers (RRIDs) for antibodies, model organisms and tools, where possible.<br><br>Have you included the information requested as detailed in our Minimum Standards Reporting Checklist?	Yes
**Availability of data and materials**	Yes

All datasets and code on which the conclusions of the paper rely must be either included in your submission or deposited in publicly available repositories (where available and ethically appropriate), referencing such data using a unique identifier in the references and in the "Availability of Data and Materials" section of your manuscript.<br><br>Have you have met the above requirement as detailed in our Minimum Standards Reporting Checklist?	

Manuscript Click here to download Manuscript Manuscript 22.3.2018.docx

Draft genome assembly of the invasive cane toad, *Rhinella marina*

Richard J Edwards[1], Daniel Enosi Tuipulotu[1†], Timothy G Amos[1†], Denis O'Meally[2], Mark F Richardson[3,4], Tonia L Russell[5], Marcelo Vallinoto[6,7], Miguel Carneiro[6], Nuno Ferrand[6,8,9], Marc R Wilkins[1,5], Fernando Sequeira[6], Lee A Rollins[3,10], Edward C Holmes[11], Richard Shine[12] and Peter A White[1,*]

[†]**These authors contributed equally to the work.**

[1]School of Biotechnology and Biomolecular Sciences, University of New South Wales, Sydney, NSW, Australia

[2]Sydney School of Veterinary Science, Faculty of Science, University of Sydney, Camperdown, New South Wales, Australia

[3]School of Life and Environmental Sciences, Centre for Integrative Ecology, Deakin University, Geelong, VIC, Australia

[4]Bioinformatics Core Research Group, Deakin University, Geelong, VIC, Australia

[5]Ramaciotti Centre for Genomics, University of New South Wales, Sydney, NSW, Australia

[6]CIBIO/*InBIO*, Centro de Investigação em Biodiversidade e Recursos Genéticos, Universidade do Porto, Vairão, Portugal

[7]Laboratório de Evolução, Instituto de Estudos Costeiros (IECOS), Universidade Federal do Pará, Bragança, Pará, Brazil

[8]Departamento de Biologia, Faculdade de Ciências, Universidade do Porto, Porto, Portugal

[9]Department of Zoology, Faculty of Sciences, University of Johannesburg, Auckland Park, South Africa

[10]Evolution and Ecology Research Centre, School of Biological Earth and Environmental Sciences, University of New South Wales, Sydney, NSW, Australia

[11]Marie Bashir Institute for Infectious Diseases and Biosecurity, Charles Perkins Centre, School of Life and Environmental Sciences and Sydney Medical School, University of Sydney, Sydney, NSW, Australia

[12]School of Life and Environmental Sciences, Faculty of Science, University of Sydney, Camperdown, New South Wales, Australia

Emails of all authors: richard.edwards@unsw.edu.au (RJE), d.enosi@unsw.edu.au (DET), t.amos@unsw.edu.au (TGA), omeally@gmail.com (DO), m.richardson@deakin.edu.au (MFR), t.russell@unsw.edu.au (TLR), mvallinoto@cibio.up.pt (MV), miguel.carneiro@cibio.up.pt (MC), nferrand@cibio.up.pt (NF), m.wilkins@unsw.edu.au (MRW), fsequeira@cibio.up.pt (FS), l.rollins@unsw.edu.au (LAR), edward.holmes@sydney.edu.au (ECH), rick.shine@sydney.edu.au (RS), p.white@unsw.edu.au (PAW).

*Corresponding author address: School of Biotechnology and Biomolecular Sciences, University of New South Wales, Sydney, NSW, Australia. Tel: +61-293853780; Email: p.white@unsw.edu.au

Abstract

Background: The cane toad (*Rhinella marina*) is a species native to Central and South America that has spread across many regions of the globe. Cane toads are known for their rapid adaptation and deleterious impacts on native fauna in invaded regions. However, despite an iconic status, there are major gaps in our understanding of cane toad genetics. The availability of a genome would help to close these gaps and accelerate cane toad research. **Findings:** We report a draft genome assembly for *R. marina*, the first of its kind for the Bufonidae family. We used a combination of long read PacBio RS II and short read Illumina HiSeq X sequencing to generate a total of 359.5 Gb of raw sequence data. The final hybrid assembly of 31,392 scaffolds was 2.55 Gb in length with a scaffold N50 of 168 kb. BUSCO analysis revealed that the assembly included full length or partial fragments of 90.6% of tetrapod universal single-copy orthologs (n=3950), illustrating that the gene-containing regions have been well-assembled. Annotation predicted 58,302 protein coding genes, with 25,846 similar to known proteins in SwissProt. Repeat sequences were estimated to account for 63.9% of the assembly. **Conclusion:** The *R. marina* draft genome assembly will be an invaluable resource that can be used to further probe the biology of this invasive species. Future analysis of the genome will provide insights into cane toad evolution and enrich our understanding of their interplay with the ecosystem at large.

Keywords: cane toad; *Rhinella marina;* sequencing; hybrid assembly; genome; annotation

Data Description

Introduction

The cane toad (*Rhinella marina*) (Figure 1) is a true toad (Bufonidae) native to Central and South America that has been introduced to many areas across the globe [1]. Since its introduction into Queensland in 1935, the cane toad has spread widely and now occupies more than 1.2 million square kilometres of the Australian continent, fatally poisoning predators like the northern quoll, freshwater crocodiles, and several species of native lizards and snakes [1-5]. The ability of cane toads to kill predators with toxic secretions has contributed to the success of their invasion [1]. To date, research on cane toads has focused primarily on ecological impacts, rapid evolution of phenotypic traits, and population genetics using neutral markers [6, 7], with limited knowledge of the genetic changes that allow the cane toad to thrive in the Australian environment [8-11]. A reference genome will be useful for studying loci subject to rapid evolution and could provide valuable insights into how invasive species adapt to new environments. Amphibian genomes have a preponderance of repetitive DNA [12, 13], confounding assembly with the limited read lengths of first- and second-generation sequencing technologies. Here, we employ a hybrid assembly of PacBio long reads and Illumina short reads (Figure 2) to overcome assembly challenges presented by the repetitive nature of the cane toad genome. Using this approach, we assembled a draft genome of *R. marina* that is comparable in contiguity and completeness to other published anuran genomes [14-17]. We used our previously published transcriptomic data [18] and other published anuran sequences to annotate the genome. Our draft cane toad assembly will serve as a reference for genetic and evolutionary studies, and provides a template for continued refinement with additional sequencing efforts.

Sample collection, library construction and sequencing

Adult female cane toads were collected by hand from Forrest River in Oombulgurri, WA (15.1818ºS, 127.8413ºE) in June 2015. Toads were placed in individual damp cloth bags and transported by plane to Sydney, NSW before they were anaesthetised by refrigeration for four hours and killed by subsequent freezing. High-molecular weight genomic DNA (gDNA) was extracted from the liver of a single female

using the genomic-tip 100/G kit (Qiagen, Hilden, Germany). This was performed with supplemental RNase (Astral Scientific, Taren Point, Australia) and proteinase K (NEB, Ipswich, MA, USA) treatment, as per the manufacturer's instructions. Isolated genomic DNA was further purified using AMPure XP beads (Beckman Coulter, Brea, CA, USA) to eliminate sequencing inhibitors. DNA quantity was assessed using the Quanti-iT PicoGreen dsDNA kit (Thermo Fisher Scientific, Waltham, MA, USA), DNA purity was calculated using a Nanodrop spectrophotometer (Thermo Fisher Scientific), and molecular integrity assessed by pulse-field gel electrophoresis.

For short read sequencing, a paired-end library was constructed from the gDNA using the TruSeq PCR-free library preparation kit (Illumina, San Diego, CA, USA). Insert sizes ranged between 200-800 bp. This library was sequenced (2 × 150 bp) on the HiSeq X Ten platform (Illumina) to generate approximately 282.9 Gb of raw data (Table 1). Illumina short sequencing reads were assessed for quality using FastQC v0.10.1 [19]. Low quality reads filtered were trimmed using Trimmomatic v0.36 [20] with a Q30 threshold (LEADING:30, TRAILING:30, SLIDINGWINDOW:4:30) and a minimum 100 bp read length, leaving 64.9% of the reads generated, of which 75.2% were in retained read pairs.

For long read sequencing, we utilised the single-molecule real time (SMRT) sequencing technology (Pacific Biosciences, Menlo Park, CA, USA). Four SMRTbell libraries were prepared from gDNA using the SMRTBell template preparation kit 1.0 (Pacific Biosciences). To increase subread length, either 15-50 kb or 20-50 kb BluePippin size selection (Sage Science, Beverly, MA, USA) was performed on each library. Recovered fragments were sequenced using P6C4 sequencing chemistry on the RS II platform (240 min movie time). The four SMRTbell libraries were sequenced on a total of 97 SMRT cells to generate 7,745,233 subreads for a total of 76.6 Gb of raw data. Collectively, short and long read sequencing produced around 359.5 Gb of data (Table 1).

Genome assembly

We employed a hybrid *de novo* whole genome assembly strategy, combining both short read and long read data. Trimmed Q30-filtered short reads were *de novo* assembled with ABySS v1.3.6 [21] using k=64 and default parameters (contig N50 = 583 bp) (Table 2). Long sequence reads were *de novo*

assembled using the program DBG2OLC [22] (k 17 AdaptiveTh 0.0001 KmerCovTh 2 MinOverlap 20 RemoveChimera 1) (contig N50 = 167.04 kbp) (Table 2). Following this, both assemblies were merged together using the hybrid assembler ('sparc') tool of DBG2OLC with default parameters, combining the contiguity of the long read data with the improved accuracy of the high coverage Illumina assembly. This hybrid assembly (v2.0) was twice 'polished' to remove errors. In the first round, the Q30 trimmed Illumina reads were mapped to the hybrid assembly with bowtie v2.2.9 [23] and filtered for proper pairs using samtools v1.3.1 [24]. The contigs were then polished with Pilon v1.21 [25] to generate the second iteration of the assembled genome (v2.1). In the second round, PacBio subreads were mapped to assembly v2.1 for error correction using SMRT analysis software (Pacific Biosciences): PacBio subreads for each library were converted to BAM format with bax2bam v0.0.08 and aligned to the genome using pbalign v.0.3.0. BAM alignment files were combined using samtools merge v1.3.1 and the contigs polished with Arrow v2.1.0 to generate the final genome assembly (v2.2). Our final draft assembly of the cane toad genome (v2.2) has 31,392 scaffolds with an N50 of 167 kb (Table 2). The GC content (43.23%) is within 1% of the published estimate of 44.17%, determined by flow cytometry [26].

Assessment of genome completeness

BUSCO [27] analysis of conserved single copy orthologues is widely used as a proxy for genome completeness and accuracy. While direct comparisons are only truly valid within an organism, comparing BUSCO scores to genomes from related organisms provides a useful benchmark. We ran BUSCO v2.0.1 (short mode, lineage tetrapoda_odb9, BLAST+ v2.2.31 [28], HMMer v3.1b2 [29], AUGUSTUS v3.2.2 [30], EMBOSS v6.5.7 [31]) on each of our assemblies, along with four published anuran genomes (Figure 3, Table 2). The hybrid assembly combined the completeness of the long read assembly with the accuracy of the short read assembly, providing an enormous boost in BUSCO completeness from less than 50% full and partial orthologs to over 90%. Error correction through pilon and arrow polishing had a positive effect on the BUSCO measurement of genome completeness, with an increase of 7.8% in the number of full and partial orthologs between v2.0 and 2.2. For the polished assembly (v2.2), 3279 (83.0%) of the 3950 ultra-conserved tetrapod genes were complete, 296 (7.5%)

were fragmentary and 375 (9.5%) were missing. By these metrics, our draft *R. marina* genome is approaching the quality and completeness of the widely used anuran amphibian reference genomes for *X. laevis* (v9.2) [17] and *X. tropicalis* (v.9.1) [16] and compares well to the recently published neobatrachian genomes of *Nanorana parkeri* (v2) [15] and *Lithobates catesbeianus* (v2.1) [14].

Estimation of *R. marina* genome size

Previous reports have estimated the size of the cane toad genome from 3.98-5.65 Gb using either densitometry or flow cytometry analysis of stained nuclei within erythrocytes, hepatocytes and renal cells [26, 32-38]. We employed two alternative strategies to measure the genome size, using short read k-mer distributions and qPCR of single copy genes. K-mer frequencies were calculated for both raw and trimmed Q30-filtered paired-end short reads (Table 1) with Jellyfish v2.2.3 [39] using k=21 and k=23, and a maximum k-mer count of 10,000. K-mer distributions were analysed using GenomeScope [40] with mean read lengths of 148 bp (raw) or 141 bp (Q30) and k-mer coverage cut-offs of 1000 and 10,000 (Table 3, Figure 4). GenomeScope gave genome size estimates ranging from 1.77 Gb to 2.30 Gb with the raw reads giving consistently larger estimates (1.85 Gb to 2.30 Gb) than the trimmed and filtered reads (1.77 Gb to 2.10 Gb). Estimates of the unique (single copy) region of the genome were more consistent, ranging from 1.31 Gb to 1.46 Gb, with k=23 estimates 99 Mb (raw) or 80 Mb (Q30) higher than k=21. Increasing the GenomeScope maximum k-mer coverage threshold had the greatest effect on predicted genome size, increasing repeat length estimates by 274 Mb to 385 Mb. GenomeScope predictions are affected by non-uniform repeat distributions and this difference could indicate high copy number repeats in the genome that are difficult to model accurately. It is possible that high frequency repeats with raw sequencing counts exceeding 10,000 are resulting in an underestimate of total repeat length and therefore genome size, compared to the previous densitometry and flow cytometry predictions.

In the second approach, the *zfp292* (zinc finger protein 292) gene was selected from our BUSCO analysis as a single-copy target for genome estimation by qPCR [41]. First, PCR was used to amplify a 326 bp region of *zfp292* (contig 6589, position 345,750-346,075) in a 25 µL reaction that contained 50 ng of gDNA, 200 µM dNTP, 0.625 units of Taq polymerase (Invitrogen), 10 × Taq

polymerase buffer (Invitrogen) and 0.4 µM of each primer (Table S1). The PCR conditions were as follows: 95ºC for 5 min, 35 cycles of 95ºC for 30 s, 60ºC for 30 s and 68ºC for 30 s followed by a final extension at 68ºC for 5 min. The amplicon was cloned into the pGEM-T Easy vector (Promega, Madison, WI, USA) and the resultant plasmid was linearised with NdeI before being serially diluted to generate a qPCR standard (10^1-10^9 copies/µL). To amplify a smaller region (120 bp) within *zfp292* (contig 6589, position 345,858-345,977) gDNA (10-25 ng) or 1 µL of the diluted standards were used as a template for a 20 µL qPCR reaction containing 2 × iTaq SYBR Green mastermix (BioRad, Hercules, CA, USA) and 0.5 µM of each primer (Table S1). The qPCR conditions were as follows: 95ºC for 10 min, 40 cycles of 95ºC for 20 s, 60ºC for 20 s and 72ºC for 20 s. Cycle threshold values obtained for each plasmid dilution were used to generate a standard curve and infer the number of *zfp292* amplicons generated from the template gDNA of known quantity. Genome sizes were generated from the formulae outlined by [41] and the average of two estimates were used to obtain a haploid genome size of 2.38 Gb. This genome size provides an estimated combined 151X sequencing coverage (119X Illumina and 32X PacBio) (Table 4).

Our genome size estimation of 1.98 to 2.38 Gbp is smaller than the 2.55 Gbp assembly size, and differs significantly from previously published estimates of 4 Gbp or more for this species. We suggest this is a result of the repetitive nature of the genome (see below). Given this is the first estimate of genome size using either k-mer or qPCR analysis, further investigations are required to more clearly understand the discrepancy in our estimates with respect to published genome sizes in anurans. Here we estimate the depth of sequencing coverage using both sequence-based and cytometric genome size measures (Table 4).

Genome annotation and gene prediction

Annotation of the draft genome was performed using MAKER2 v2.31.6 [42], BLAST+ v2.2.31 [28], AUGUSTUS v3.2.2 [30], Exonerate v2.2.0 [43], RepeatMasker v4.0.6 [44] (DFAM [45], Library Dfam_1.2; RMLibrary v20150807), RepeatModeler v1.0.8 [46] and SNAP v2013-11-29 [47] using all SwissProt protein sequences (downloaded 2017-02-23)[48] . AUGUSTUS was trained using BUSCO

v2.0.1 (long mode, lineage tetrapoda_odb9) and a multi-tissue reference transcriptome we previously generated from tadpoles and six adult cane toad tissues [18] (available from GigaDB [49], Genbank accession PRJNA383966). After the initial training run, two further iterations of MAKER2 were run using HMMs from SNAP training created from the previous run. Functional annotation of protein-coding genes predicted by MAKER2 were generated using Interproscan 5.25-64.0, with the following settings: -dp -t p -pa -goterms -iprlookup -appl TIGRFAM, SFLD, Phobius, SUPERFAMILY, PANTHER, Gene3D, Hamap, ProSiteProfiles, Coils, SMART, CDD, PRINTS, ProSitePatterns, SignalP_EUK, Pfam, ProDom, MobiDBLite, PIRSF, TMHMM. BLAST+ v2.6.0 [28] was used to annotate predicted genes using all Swissprot proteins (release 2017_08, downloaded 2017-09-01) [48] using the following settings: -evalue 0.000001 -seg yes -soft_masking true -lcase_masking -max_hsps 1.

In total, 58,302 protein-coding genes were predicted by the MAKER pipeline with an average of 5.3 exons and 4.3 introns per gene (Table 5). Of these, 5,225 are single exon genes, giving 4.7 introns per multi-exon gene with an average intron length of 4.08 kb. Predicted coding sequences make up 2.38% of the assembly. MAKER predicted considerably more than the approimately twenty thousand genes expected for a typical vertebrate genome. There are two likely explanations for this: (1) artefactual duplications in the genome assembly, either through under-assembly or legitimate assembly of two heterozygous diploid copies; (2) over-prediction of proteins during genome annotation, including pseudogenes with high homology to functional genes. Of the 3,279 complete BUSCO genes identified (Table 2), only 85 (2.59%) were duplicated. This suggests that there is not widespread duplication in the assembly. Only 25,846 predicted genes were similar to known proteins in SwissProt, with the remaining 32,456 predictions "of unknown function". This is consistent with over-prediction being the primary cause of inflated gene numbers. The predicted proteins of unknown function have a very different size distribution (median length 171 aa) to those with Swissprot hits (median length 388 aa). To investigate this further, predicted transcript and protein sequences were searched against the published *de novo* assembled transcriptome [18] using BLAST+ v2.2.31 [28] blastn or tblastn (top 10 hits, e-value < 10^{-10}) and compiled with GABLAM v2.28.3 [50]. For 56.5% of proteins with functional

annotation, 95%+ of the protein length mapped to the top transcript hit (Table 6). Only 27.1% of unknown proteins had 95%+ coverage in the top transcript hit, which is again consistent with over-prediction. It should also be noted that some of the predicted genes may represent lncRNA genes that have been incorrectly assigned a coding sequence.

Repeat identification and analysis

The cane toad genome has proven very difficult to assemble using short reads alone, which suggests a high frequency of repetitive sequences, as for other amphibians [12, 13]. RepeatMasker annotations from the MAKER pipeline support this interpretation, with over 4.1 million repeat sequences detected, accounting for 63.9% of the assembly (Table 5). Critically, the average length of most of these repeat classes exceed the Illumina read length, rendering accurate assembly with short reads impossible. The most abundant class of repeat elements are of unknown type (1.61 million elements covering 32.28% of the assembly), with DNA transposons the most abundant known class of element (817,262 repeats; 19.17% coverage). Of these, the most abundant are of the hAT-Ac (231,332 copies) and TcMar-Tc1 (226,145 copies) superfamilies (Table S2). Accounting for overlaps between repeat and gene features, 18.7% of the assembly (479,397,014 bp) has no annotation (Figure 5).

Conclusion

This draft genome assembly sets a milestone in the field of anuran genetics and will be an invaluable tool for advancing knowledge of anuran biology, genetics and the evolution of invasive species. Furthermore, we envisage these data will facilitate the development of biocontrol strategies that reduce the impact of cane toads on native fauna.

Availability of supporting data

Raw genomic sequencing data (Illumina and PacBio) and assembled scaffolds have been deposited in the ENA with the study accession PRJEB24695 and assembly accession GCA_900303285. The genome assembly and annotation are also available in the *GigaScience* database.

List of abbreviations

BUSCO: Benchmarking Universal Single-Copy Orthologs; qPCR: quantitative polymerase chain reaction, CDS: coding sequence; bp: base pair; gDNA: genomic DNA; SMRT: single-molecule real time; SINE: short interspersed nuclear element; LINE: long interspersed nuclear element, LTR: long terminal repeat; UTR: untranslated region

Additional files

Table S1. Primers used for genome size estimation by single copy gene qPCR.

Table S2. RepeatMasker statistics broken down by repeat category.

Ethics approval and consent to participate

All experimentation was performed under the approval of the University of Sydney Animal Ethics Committee.

Consent for publication

Not applicable

Competing interests

The authors declare that they have no competing interests.

Funding

This work was supported by the Cane Toad Genome Consortium and Bioplatforms Australia. Funding was provided by the Australian Research Council (DP160102991). L.A.R. is funded by ARC DECRA Fellowship (DE150101393). R.S. is funded by ARC Australian Laureate Fellowship (FL120100074). E.C.H. is funded by ARC Australian Laureate Fellowship (FL170100022). M.R.W. and the Ramaciotti Centre for Genomics acknowledge infrastructure funding from the Australian Research Council (LE150100031), the Australian Government NCRIS scheme administered by Bioplatforms Australia and the New South Wales Government RAAP scheme. M.V. is supported by a CNPq fellowship

(302892/2016-8). D.E.T. was supported through an Australian Government Research Training Program (RTP) scholarship.

Author's contributions

P.A.W coordinated the project. P.A.W, R.S, E.C.H, L.A.R, R.J.E, M.W. designed the study. P.A.W, R.S, E.C.H, L.A.R, R.J.E and F.S funded the project. R.S provided the cane toad samples. D.E.T performed the genomic DNA extraction, PCR experiments and data analysis. T.L.R performed the sequencing. R.J.E and T.G.A performed the genome assemblies and primary data analysis. D.O and T.G.A. performed the genome annotation. R.J.E, D.E.T, T.G.A and P.A.W and wrote the manuscript. All authors edited and approved the final manuscript.

Acknowledgements

We thank the Ramaciotti Centre for Genomics, UNSW and the Kinghorn Centre for Clinical Genomics at the Garvan Institute of Medical Research who performed the sequencing in this study. This project was undertaken with the assistance of resources and services from the National Computational Infrastructure (NCI), which is supported by the Australian Government. This project used computational resources from Intersect, which were in part supported by the Australian Research Council. The authors acknowledge UNSW Science's high performance computing cluster (Katana), the UNSW School of Biotechnology and Biomolecular Sciences high memory node, the Sydney Informatics Hub and the University of Sydney's high performance computing cluster (Artemis) for providing the high performance computing resources that have contributed to the research results reported within this paper. We thank the members of the UNSW Science Faculty Computing unit, particularly Duncan Smith, for all his help getting software installed and running on the katana computer cluster. We thank Dr Andrew Kelly for early discussions on cane toad genome size and technical assistance with gDNA extractions.

References

1. Shine R. The ecological impact of invasive cane toads (Bufo marinus) in Australia. The Quarterly Review of Biology. 2010;85 3:253-91.
2. Phillips BL, Brown GP, Greenlees M, Webb JK and Shine R. Rapid expansion of the cane toad (Bufo marinus) invasion front in tropical Australia. Austral Ecology. 2007;32 2:169-76.
3. Phillips BL, Brown GP and Shine R. Assessing the potential impact of cane toads on Australian snakes. Conservation Biology. 2003;17 6:1738-47.
4. Smith JG and Phillips BL. Toxic tucker: the potential impact of cane toads on Australian reptiles. Pacific Conservation Biology. 2006;12 1:40-9.
5. Urban MC, Phillips BL, Skelly DK and Shine R. The cane toad's (Chaunus [Bufo] marinus) increasing ability to invade Australia is revealed by a dynamically updated range model. Proceedings of the Royal Society of London B: Biological Sciences. 2007;274 1616:1413-9.
6. Slade R and Moritz C. Phylogeography of Bufo marinus from its natural and introduced ranges. Proceedings of the Royal Society of London B: Biological Sciences. 1998;265 1398:769-77.
7. Sequeira F, Sodré D, Ferrand N, Bernardi JA, Sampaio I, Schneider H, et al. Hybridization and massive mtDNA unidirectional introgression between the closely related Neotropical toads Rhinella marina and R. schneideri inferred from mtDNA and nuclear markers. BMC evolutionary biology. 2011;11 1:264.
8. Rollins LA, Richardson MF and Shine R. A genetic perspective on rapid evolution in cane toads (Rhinella marina). Molecular Ecology. 2015;24 9:2264-76.
9. Estoup A, Baird SJ, Ray N, Currat M, CORNUET J, Santos F, et al. Combining genetic, historical and geographical data to reconstruct the dynamics of bioinvasions: application to the cane toad Bufo marinus. Molecular ecology resources. 2010;10 5:886-901.
10. Trumbo DR, Epstein B, Hohenlohe PA, Alford RA, Schwarzkopf L and Storfer A. Mixed population genomics support for the central marginal hypothesis across the invasive range of the cane toad (Rhinella marina) in Australia. Molecular ecology. 2016;25 17:4161-76.
11. Leblois R, Rousset F, Tikel D, Moritz C and Estoup A. Absence of evidence for isolation by distance in an expanding cane toad (Bufo marinus) population: an individual-based analysis of microsatellite genotypes. Molecular Ecology. 2000;9 11:1905-9.
12. Bozzoni I and Beccari E. Clustered and interspersed repetitive DNA sequences in four amphibian species with different genome size. Biochimica et Biophysica Acta (BBA)-Nucleic Acids and Protein Synthesis. 1978;520 2:245-52.
13. Olmo E. Genome variations in the transition from amphibians to reptiles. Journal of molecular evolution. 1991;33 1:68-75.
14. Hammond SA, Warren RL, Vandervalk BP, Kucuk E, Khan H, Gibb EA, et al. The North American bullfrog draft genome provides insight into hormonal regulation of long noncoding RNA. Nature Communications. 2017;8 1:1433.
15. Sun Y-B, Xiong Z-J, Xiang X-Y, Liu S-P, Zhou W-W, Tu X-L, et al. Whole-genome sequence of the Tibetan frog Nanorana parkeri and the comparative evolution of tetrapod genomes. Proceedings of the National Academy of Sciences. 2015;112 11:E1257-E62.
16. Hellsten U, Harland RM, Gilchrist MJ, Hendrix D, Jurka J, Kapitonov V, et al. The genome of the Western clawed frog Xenopus tropicalis. Science. 2010;328 5978:633-6.
17. Session AM, Uno Y, Kwon T, Chapman JA, Toyoda A, Takahashi S, et al. Genome evolution in the allotetraploid frog Xenopus laevis. Nature. 2016;538 7625:336.
18. Richardson MF, Sequeira F, Selechnik D, Carneiro M, Vallinoto M, Reid JG, et al. Improving amphibian genomic resources: a multi-tissue reference transcriptome of an iconic invader. GigaScience. 2017;7 1:1-7.
19. Andrews S. FastQC: a quality control tool for high throughput sequence data. 2010.
20. Bolger AM, Lohse M and Usadel B. Trimmomatic: a flexible trimmer for Illumina sequence data. Bioinformatics. 2014;30 15:2114-20.
21. Simpson JT, Wong K, Jackman SD, Schein JE, Jones SJ and Birol I. ABySS: a parallel assembler for short read sequence data. Genome research. 2009;19 6:1117-23.

22. Ye C, Hill CM, Wu S, Ruan J and Ma ZS. DBG2OLC: efficient assembly of large genomes using long erroneous reads of the third generation sequencing technologies. Scientific reports. 2016;6:31900.
23. Langmead B and Salzberg SL. Fast gapped-read alignment with Bowtie 2. Nature methods. 2012;9 4:357-9.
24. Li H, Handsaker B, Wysoker A, Fennell T, Ruan J, Homer N, et al. The sequence alignment/map format and SAMtools. Bioinformatics. 2009;25 16:2078-9.
25. Walker BJ, Abeel T, Shea T, Priest M, Abouelliel A, Sakthikumar S, et al. Pilon: an integrated tool for comprehensive microbial variant detection and genome assembly improvement. PloS one. 2014;9 11:e112963.
26. Vinogradov AE. Genome size and GC-percent in vertebrates as determined by flow cytometry: The triangular relationship. Cytometry Part A. 1998;31 2:100-9.
27. Simão FA, Waterhouse RM, Ioannidis P, Kriventseva EV and Zdobnov EM. BUSCO: assessing genome assembly and annotation completeness with single-copy orthologs. Bioinformatics. 2015;31 19:3210-2.
28. Camacho C, Coulouris G, Avagyan V, Ma N, Papadopoulos J, Bealer K, et al. BLAST+: architecture and applications. BMC bioinformatics. 2009;10 1:421.
29. Mistry J, Finn RD, Eddy SR, Bateman A and Punta M. Challenges in homology search: HMMER3 and convergent evolution of coiled-coil regions. Nucleic acids research. 2013;41 12:e121-e.
30. Keller O, Kollmar M, Stanke M and Waack S. A novel hybrid gene prediction method employing protein multiple sequence alignments. Bioinformatics. 2011;27 6:757-63.
31. Rice P, Longden I and Bleasby A. EMBOSS: the European molecular biology open software suite. Elsevier Current Trends, 2000.
32. Bachmann K. Specific nuclear DNA amounts in toads of the genus Bufo. Chromosoma. 1970;29 3:365-74.
33. Camper J, Ruedas L, Bickham J and Dixon J. The relationship of genome size with developmental rates and reproductive strategies in five families of neotropical bufonoid frogs. Life Sci Adv. 1993;12:79-87.
34. Bachmann K. Nuclear DNA and developmental rate in frogs. Quarterly Journal of the Florida Academy of Sciences. 1972;35 4:225-31.
35. Chipman AD, Khaner O, Haas A and Tchernov E. The evolution of genome size: what can be learned from anuran development? Journal of Experimental Zoology Part A: Ecological Genetics and Physiology. 2001;291 4:365-74.
36. Griffin C, Scott D and Papworth D. The influence of DNA content and nuclear volume on the frequency of radiation-induced chromosome aberrations in Bufo species. Chromosoma. 1970;30 2:228-49.
37. Goin OB, Goin CJ and Bachmann K. DNA and amphibian life history. Copeia. 1968:532-40.
38. MacCulloch RD, Upton DE and Murphy RW. Trends in nuclear DNA content among amphibians and reptiles. Comparative Biochemistry and Physiology Part B: Biochemistry and Molecular Biology. 1996;113 3:601-5.
39. Marçais G and Kingsford C. A fast, lock-free approach for efficient parallel counting of occurrences of k-mers. Bioinformatics. 2011;27 6:764-70.
40. Vurture GW, Sedlazeck FJ, Nattestad M, Underwood CJ, Fang H, Gurtowski J, et al. GenomeScope: fast reference-free genome profiling from short reads. Bioinformatics. 2017;33 14:2202-4.
41. Wilhelm J, Pingoud A and Hahn M. Real-time PCR-based method for the estimation of genome sizes. Nucleic Acids Research. 2003;31 10:e56-e.
42. Holt C and Yandell M. MAKER2: an annotation pipeline and genome-database management tool for second-generation genome projects. BMC bioinformatics. 2011;12 1:491.
43. Slater GSC and Birney E. Automated generation of heuristics for biological sequence comparison. BMC bioinformatics. 2005;6 1:31.
44. Smit AFA, Hubley R and Green P. 2013–2015. RepeatMasker Open-4.0. 2013. http://www.repeatmasker.org/.

45. Hubley R, Finn RD, Clements J, Eddy SR, Jones TA, Bao W, et al. The Dfam database of repetitive DNA families. Nucleic acids research. 2015;44 D1:D81-D9.
46. Smit AFA and Hubley R. 2008–2015. RepeatModeler Open-1.0. 2008. http://www.repeatmasker.org/.
47. Korf I. Gene finding in novel genomes. BMC bioinformatics. 2004;5 1:59.
48. Apweiler R, Bairoch A, Wu CH, Barker WC, Boeckmann B, Ferro S, et al. UniProt: the Universal Protein knowledgebase. Nucleic Acids Research. 2004;32 suppl_1:D115-D9. doi:10.1093/nar/gkh131.
49. Richardson MF, Sequeira F, Selechnik D, Carneiro M, Vallinoto M, Reid JG, et al. Supporting data for “Improving amphibian genomic resources: a multitissue reference transcriptome of an iconic invader.” Giga-Science Database 2017. http://dx.doi.org/10.5524/100374.
50. Davey NE, Shields DC and Edwards RJ. SLiMDisc: short, linear motif discovery, correcting for common evolutionary descent. Nucleic acids research. 2006;34 12:3546-54.
51. Hedges SB, Dudley J and Kumar S. TimeTree: a public knowledge-base of divergence times among organisms. Bioinformatics. 2006;22 23:2971-2.
52. Kumar S, Stecher G and Tamura K. MEGA7: molecular evolutionary genetics analysis version 7.0 for bigger datasets. Molecular biology and evolution. 2016;33 7:1870-4.

Tables

Table 1. Summary statistics of generated whole genome shotgun sequencing data. Bold rows indicate data used for assembly.

Platform	Library Type	Mean insert size (kb)	Mean read length (bp)	Number of reads	Number of bases (Gb)
HiSeqX (raw)	Paired-end	0.35	147.7	1,857,762,090	282.92
**HiSeqX (filtered)**			**140.6**	**1,205,616,705**	**169.47**
PacBio RS II	SMRTbell	15-50	8,852	2,794,391	24.736
PacBio RS II	SMRTbell	15-50	9,085	595,447	5.409
PacBio RS II	SMRTbell	15-50	10,432	1,867,543	19.482
PacBio RS II	SMRTbell	20-50	10,834	2,487,852	26.952
**PacBio Total**			**9,887**	**7,745,233**	**76.58**
**PacBio Unique[1]**			**10,987**	**6,167,714**	**67.77**

1. Longest read per sequenced molecule (SMRT ZMW).

Table 2. Summary of genome assemblies. For comparison, statistics are provided for two existing neobatrachian genomes, *Nanorana parkeri* (v2) [15] and *Lithobates catesbeianus* (v2.1)[14], and two anuran reference genomes, *Xenopus tropicalis* (v9.1) [16] and *X. laevis* (v9.2) [17]. Lengths are given to 3 s.f.

**Genome Assembly**	**Hybrid (v2.2)**	**Short read**	**Long read**	***N. parkeri* (v2.0)**	***L. catesbeianus* (v2.1)**	***X. tropicalis* (v9.1)**	***X. laevis* (v9.2)**
Total Length (Gb)	2.55	3.75	2.69	2.07	6.25	1.44	2.72
No. scaffolds	31,392	19.9 M*	31,392*	135,808	1.54 M	6,822	108,033
Proportion gap (%N)	0.00%	0.14%	0.00%	3.86%	11.58%	4.90%	11.39%
N50	168 kb	583 bp	167 kb	1.06 Mb	39.4 kb	135 Mb	137 Mb
L50	3,373	715 k	3,531	555	31,248	5	9
Longest scaffold	3.53 Mb	72.6 kb	3.64 Mb	8.61 Mb	1.38 Mb	195 Mb	220 Mb
GC	43.23%	43.25%	42.88%	42.58%	43.14%	40.07%	38.98%
**BUSCO[1]**							
Complete Single copy	80.9%	15.5%	2.2%	83.4%	42.3%	87.5%	52.9%
Complete Duplicate	2.2%	0.7%	0.0%	1.6%	0.9%	1.0%	39.8%
Fragment	7.5%	33.6%	2.2%	7.2%	22.3%	6.0%	3.2%

1. BUSCO v2.0.1 short summary statistics (n=3950).

* Statistics for short and long read assemblies refer to contigs used for hybrid assembly.

Table 3. GenomeScope genome size estimates for Rhinella marina based on raw trimmed Illumina data using different combinations of k and maximum k-mer coverage. Lengths are in megabases (0 d.p.).

Data	Max kmer coverage	Unique Length (Mb)		Repeat Length (Mb)		Haploid Genome Size (Mb)	
		Min	Max	Min	Max	Min	Max
Raw (k=21)	1000	1,365	1,366	489	489	1,853	1,855
Raw (k=21)	10000	1,365	1,365	874	874	2,239	2,240
Raw (k=23)	1000	1,453	1,455	470	471	1,924	1,926
Raw (k=23)	10000	1,454	1,454	842	842	2,296	2,296
Q30 (k=21)	1000	1,307	1,308	462	462	1,768	1,771
Q30 (k=21)	10000	1,307	1,308	749	749	2,056	2,057
Q30 (k=23)	1000	1,389	1,391	438	439	1,828	1,830
Q30 (k=23)	10000	1,390	1,391	713	713	2,103	2,104

Table 4. Estimation of Rhinella marina genome size using various methods and the corresponding level of sequencing coverage (3 s.f.). GenomeScope values in this table are mean values from the four setting combinations.

Method	Estimated Genome Size (Gb)	Illumina coverage (X)	PacBio coverage (X)	Reference
Flow cytometry (mean)	4.33	65.3	17.7	[26, 33, 35, 38]
Flow cytometry (min)	3.98	71.1	19.2	[38]
Flow cytometry (max)	4.90	57.7	15.6	[35]
Densitometry (mean)	4.95	57.1	15.5	[32, 34, 36, 37]
Densitometry (min)	4.06#	69.7	18.9	[37]
Densitometry (max)	5.65	50.1	13.6	[32]
GenomeScope (raw)	2.08	136	36.8	-
GenomeScope (Q30)	1.94	146	39.4	-
qPCR (zfp292)	2.38	119	32.1	-
Assembly (v2.2)	2.55	111	30.0	-

value adjusted to account for updated size of reference genome used to infer R. marina genome size.

Table 5. Summary statistics of consensus protein-coding gene predictions and predicted repeat elements (including RNA genes) for the *Rhinella marina* v2.2 draft genome. Lengths are given to 3 s.f. Coverage and mean depth statistics for PacBio and Q30-trimmed Illumina reads are given to 2 d.p.

Element	Count	No. scaffolds	Avg. length	Total length	Genome coverage	PacBio depth (X)	Illumina depth (X)
Protein-coding gene	58,302	19,530	18.8 kb	1.10 Gb	42.91%	20.32	58.07
Transcript	58,302	19,530	1.24 kb	72.3 Mb	2.83%	20.49	65.41
- Similar to known	25,846	11,918	1.90 kb	49.1 Mb	1.92%	20.08	56.42
- Unknown	32,456	15,213	714 bp	23.2 Mb	0.91%	20.98	68.82
Exon	309,718	19,530	233 bp	72.3 Mb	2.83%	20.49	65.41
- Coding	294,535	19,530	207 bp	60.8 Mb	2.38%	20.67	66.97
Intron	251,416	18,509	4.08 kb	1.03 Gb	40.09%	20.30	57.55
5’ UTR	15,855	8,839	208 bp	3.29 Mb	0.13%	18.69	53.86
CDS	58,302	19,530	1.04 kb	60.8 Mb	2.38%	20.67	66.97
3’ UTR	11,965	5,780	682 bp	8.16 Mb	0.32%	19.91	58.52
BUSCO SC Complete	3,194	2,014	32.6 kb	104 Mb	4.07%	19.89	53.01
**Repeats**							
SINE	21,620	9,322	338 bp	7.31 Mb	0.29%	19.45	58.23
LINE	268,569	27,620	513 bp	138 Mb	5.38%	21.03	72.29
LTR	201,817	24,949	504 bp	102 Mb	3.98%	22.62	68.96
DNA	817,405	30,689	600 bp	490 Mb	19.17%	21.67	68.37
Helitron	20,319	9,340	826 bp	16.8 Mb	0.66%	19.32	56.81
Retroposon	1,042	829	549 bp	570 kb	0.02%	18.22	50.87
Other	18	17	209 bp	3.7 kb	0.00%	14.27	24.60
Unknown	1,610,883	30,966	513 bp	826 Mb	32.28%	20.12	59.39
Satellite	25,557	10,270	440 bp	11.3 Mb	0.44%	18.38	54.21
Simple repeats	968,947	30,620	56.9 bp	55.1 Mb	2.16%	18.88	48.51
Low complexity	141,028	24,020	51.8 bp	7.30 Mb	0.29%	22.48	64.48
rRNA	5,227	2,923	422 bp	2.20 Mb	0.09%	40.88	142.42
tRNA	5,558	4,474	105 bp	583 kb	0.02%	29.15	140.06
snRNA	21,788	9,432	546 bp	11.9 Mb	0.47%	24.63	89.12

srpRNA	17	11	268 bp	4.55 kb	0.00%	22.11	140.44
scRNA	3	3	69.0 bp	207 bp	0.00%	15.53	47.29
RNA	418	266	482 bp	202 kb	0.01%	32.65	173.99
**Repeat TOTAL**[1]	4,110,222	31,179	406 bp	1.63 Gb	63.9%	20.82	63.79

1. Values for repeat totals account for overlapping repeats.

Table 6. Proportions of predicted protein and transcript sequences exceeding 50%, 80%, 95% or 99% coverage in the top BLAST+ hit from the published transcriptome [18], and combined coverage for the top ten transcript hits. All percentages given to 3 s.f.

Type	Count	Coverage in top transcript hit				Coverage in top 10 transcript hits			
		50%+	80%+	95%+	99%+	50%+	80%+	95%+	99%+
Protein (similar to known)	25,846	93.6	76.7	56.5	40.7	97.5	90.3	72.7	54.2
Transcript (similar to known)	25,846	75.0	50.0	30.8	21.4	82.6	73.1	57.2	40.9
Protein (unknown)	32,456	79.9	49.8	27.1	15.8	85.7	66.3	44.4	29.9
Transcript (unknown)	32,456	43.6	21.5	12.1	8.61	52.6	37.3	25.4	19.1

Figure legends

Figure 1. *Rhinella marina.* (A) An adult cane toad. (B) Phylogenetic tree of the five frog and toad species used in this study, plus human as a reference. Taxonomic relationships and estimated divergence times are from TimeTree [51] and visualised with MEGA7 [52]. Branch lengths indicate approximate divergence times in millions of years (0 d.p.).

Figure 2. Schematic overview of project workflow. A summary of the experimental methods used for sequencing, assembly, annotation and size estimation of the cane toad genome. Transcriptome data (orange segment) was obtained from our previous study [18].

Figure 3. Assessment of genome assembly completeness. BUSCO analysis of *Rhinella marina* genome assembly (v2.0 uncorrected, v2.1 pilon polishing, v2.2 pilon and arrow polishing), *Lithobates catesbeianus* (v2.1), *Nanorana parkeri* (v2.0), *Xenopus tropicalis* (v9.1) and *X. leavis* (v9.2) genomes using the tetrapoda_odb9 orthologue set (n=3950). The *X. leavis* genome duplication is made clear by the large number of paralogs (light blue) with respect to other assemblies.

Figure 4. GenomeScope k-mer frequency and log-transformed k-mer coverage profiles. (A) raw Illumina data (k=23), (B) Q30 trimmed Illumina data (k=23). Profiles for k=21 are similar (data not shown).

Figure 5. Summary of the main annotation classes for *Rhinella marina* genome assembly. Identified repeat classes exceeding 2% of assembly have been plotted separately (1 d.p.). All other repeats, including “Unknown”, have been grouped as “Other repeats”. The percentage for introns excludes any repeat sequences within those introns.

Figure 1

Click here to download Figure FIgure 1.eps

A. B.

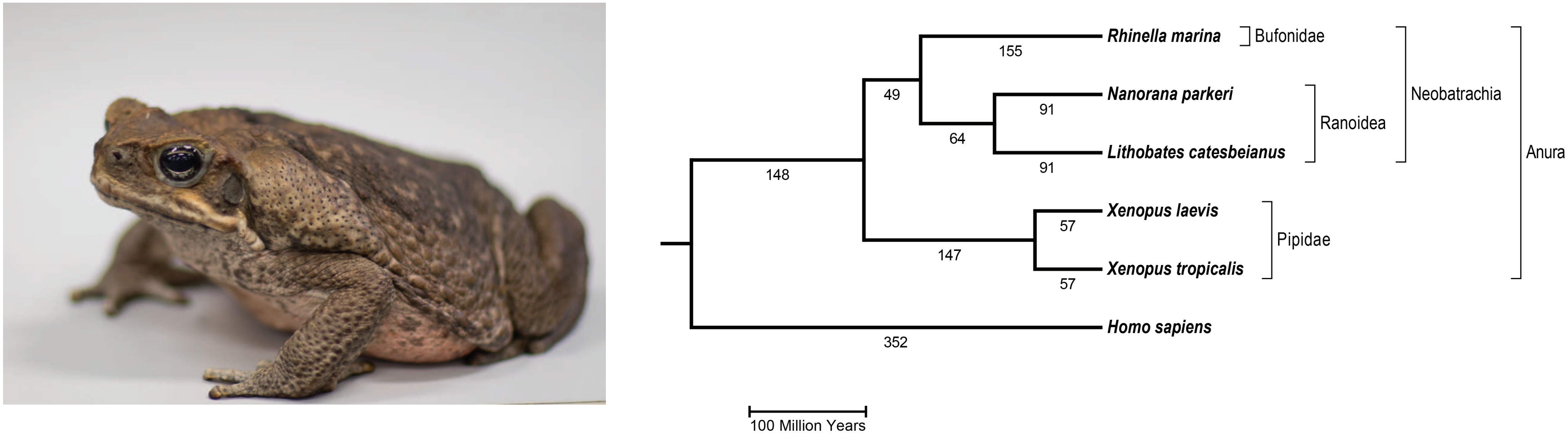

Figure 2

Click here to download Figure Figure 2.eps

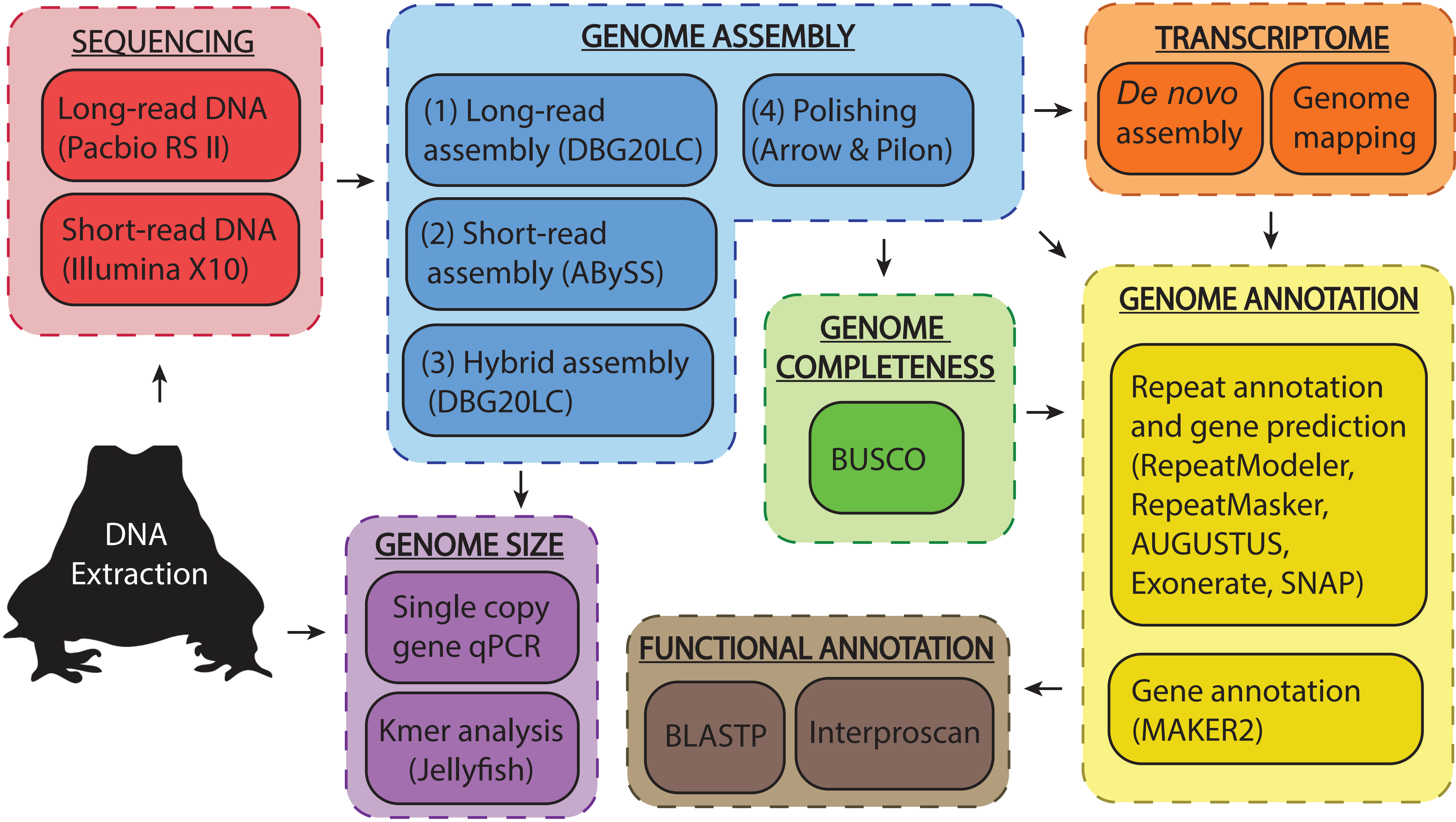

Figure 3

Click here to download Figure Figure 3.pdf

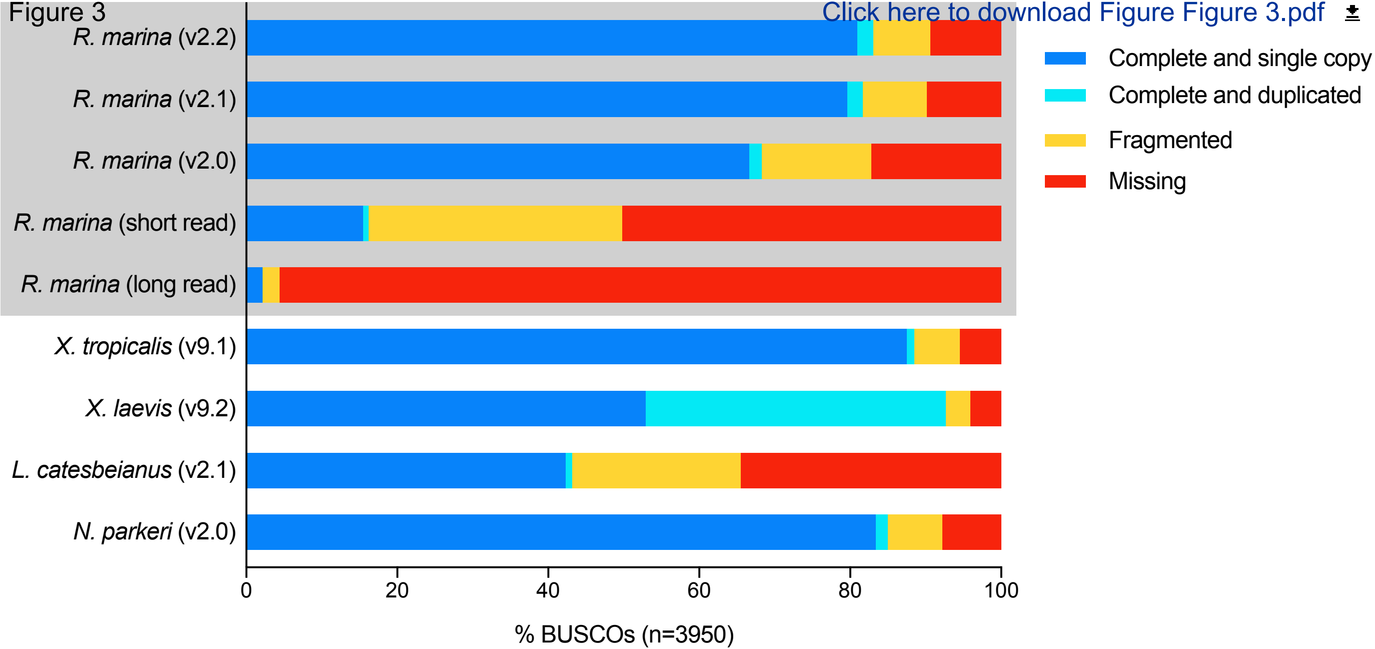

Figure 4

Click here to download Figure Figure 4.eps

A. Raw data (k=23)

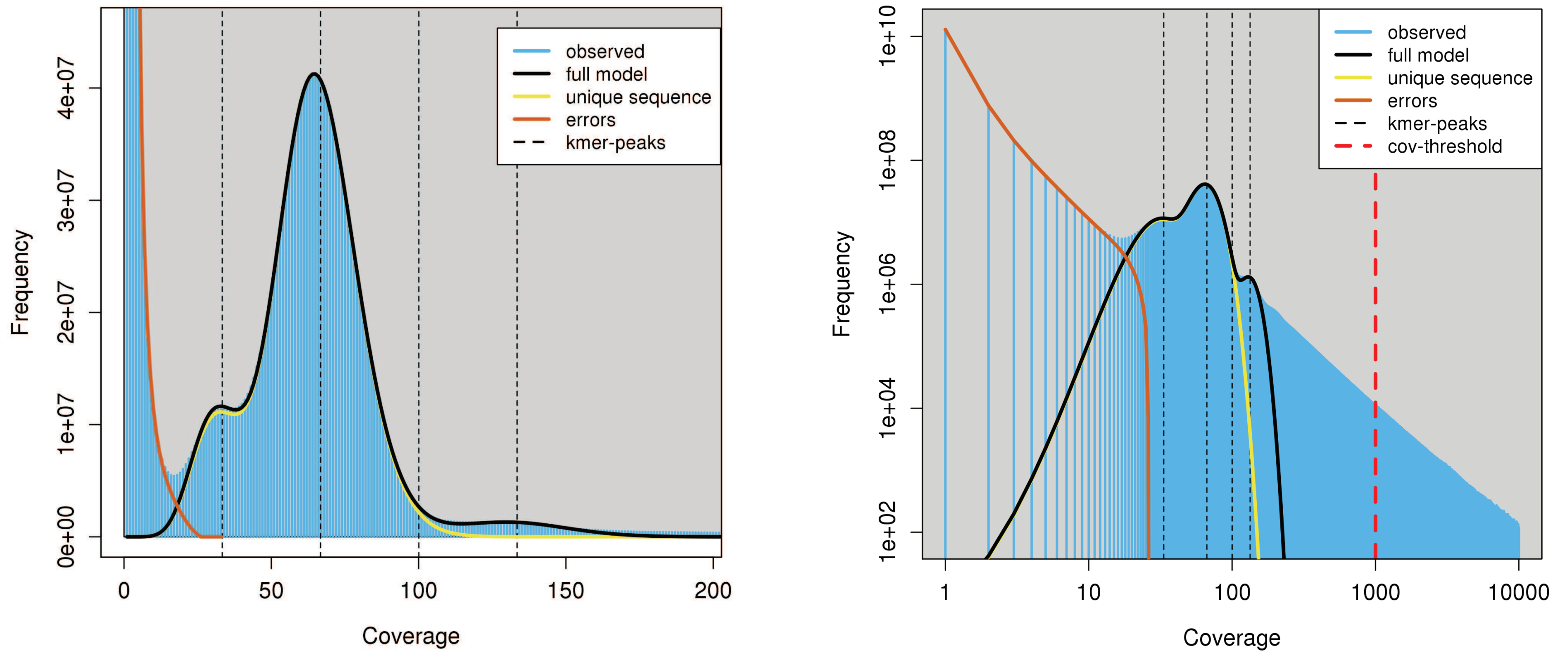

B. Q30 trimmed data (k=23)

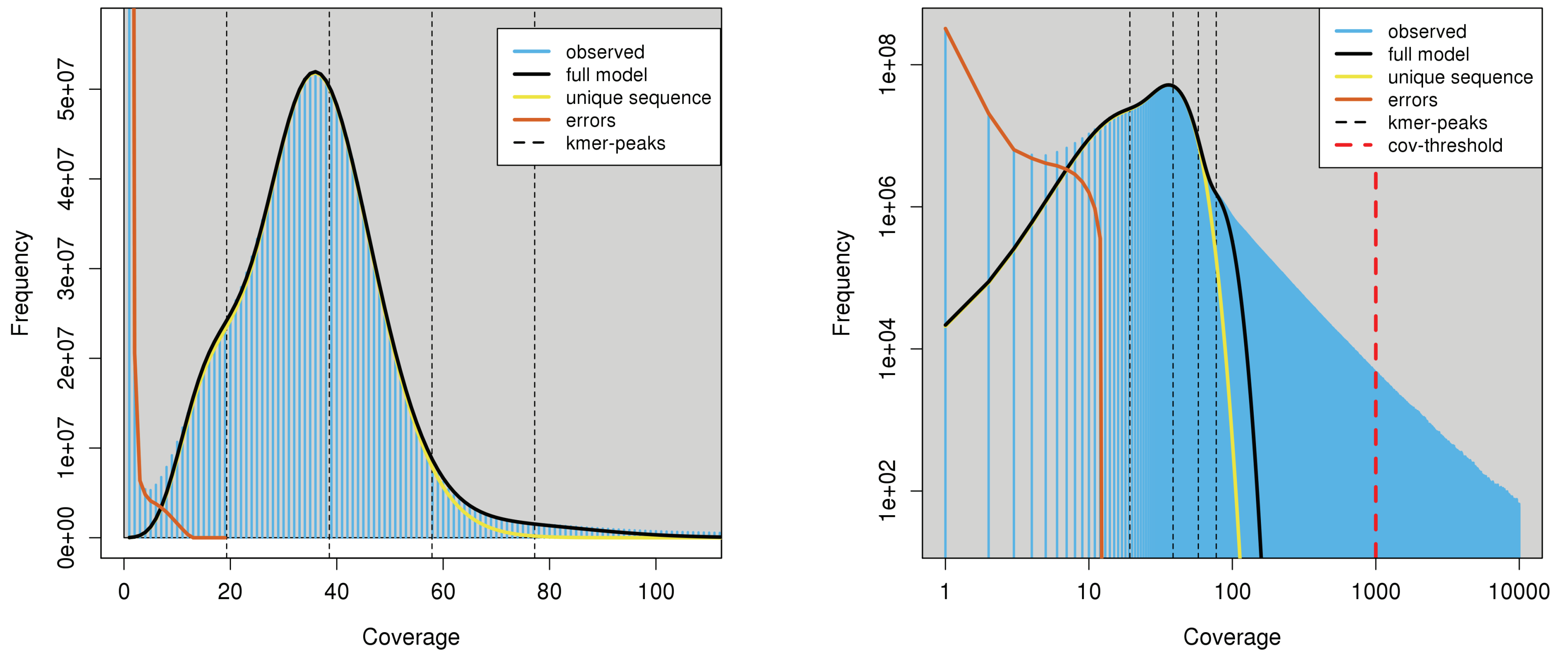

Figure 5

Click here to download Figure Figure 5.pdf

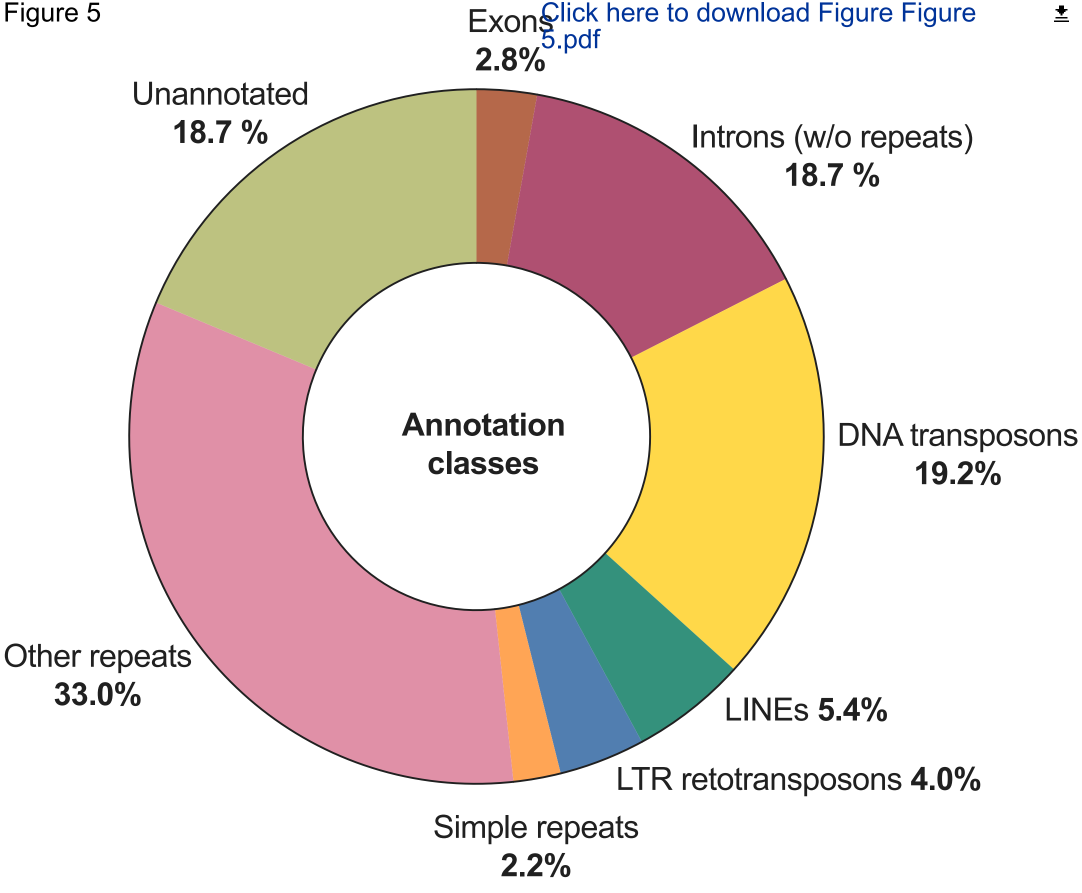

Supplementary Table 1

Click here to access/download
Supplementary Material
Table S1.xlsx

Supplementary Table 2

Click here to access/download
Supplementary Material
Table S2.xlsx
