# Supplementary material for: Draft genome assembly of the invasive cane toad, Rhinella marina
Source: Gigascience. 2018 Aug 7;7(9):giy095. doi: 10.1093/gigascience/giy095 (PMC6145236; doi:10.1093/gigascience/giy095)
Supplement: GIGA-D-18-00104_Revision_2.pdf [file giy095_giga-d-18-00104_revision_2.pdf]

GigaScience

Draft genome assembly of the invasive cane toad, Rhinella marina

--Manuscript Draft--

**Manuscript Number:**	GIGA-D-18-00104R2
**Full Title:**	Draft genome assembly of the invasive cane toad, Rhinella marina
**Article Type:**	Data Note
**Funding Information:**	Australian Research Council (DP160102991) — Dr. Lee A Rollins; Prof. Richard Shine<br>Australian Research Council (DE150101393) — Dr. Lee A Rollins<br>Australian Research Council (FL170100022) — Prof. Edward C Holmes<br>Australian Research Council (LE150100031) — Prof. Marc R Wilkins<br>Australian Research Council (FL120100074) — Prof. Richard Shine<br>The National Council for Scientific and Technological Development (CNPq) (302892/2016-8) — Assoc. Prof. Marcelo Vallinoto<br>Bioplatforms Australia — Not applicable
**Abstract:**	Background: The cane toad (Rhinella marina formerly Bufo marinus) is a species native to Central and South America that has spread across many regions of the globe. Cane toads are known for their rapid adaptation and deleterious impacts on native fauna in invaded regions. However, despite an iconic status, there are major gaps in our understanding of cane toad genetics. The availability of a genome would help to close these gaps and accelerate cane toad research. Findings: We report a draft genome assembly for R. marina, the first of its kind for the Bufonidae family. We used a combination of long read PacBio RS II and short read Illumina HiSeq X sequencing to generate a total of 359.5 Gb of raw sequence data. The final hybrid assembly of 31,392 scaffolds was 2.55 Gb in length with a scaffold N50 of 168 kb. BUSCO analysis revealed that the assembly included full length or partial fragments of 90.6% of tetrapod universal single-copy orthologs (n=3950), illustrating that the gene-containing regions have been well-assembled. Annotation predicted 58,302 protein coding genes, with 25,846 similar to known proteins in SwissProt. Repeat sequences were estimated to account for 63.9% of the assembly. Conclusion: The R. marina draft genome assembly will be an invaluable resource that can be used to further probe the biology of this invasive species. Future analysis of the genome will provide insights into cane toad evolution and enrich our understanding of their interplay with the ecosystem at large.
**Corresponding Author:**	Peter White, Ph.D.<br>University of New South Wales<br>Sydney, NSW AUSTRALIA
**Corresponding Author Secondary Information:**	
**Corresponding Author's Institution:**	University of New South Wales
**Corresponding Author's Secondary Institution:**	
**First Author:**	Richard J Edwards
**First Author Secondary Information:**	
**Order of Authors:**	Richard J Edwards
	Daniel Enosi Tuipulotu
	Timothy G Amos

Powered by Editorial Manager® and ProduXion Manager® from Aries Systems Corporation

	Denis O'Meally
	Mark F Richardson
	Tonia L Russell
	Marcelo Vallinoto
	Miguel Carneiro
	Nuno Ferrand
	Marc R Wilkins
	Fernando Sequeira
	Lee A Rollins
	Edward C Holmes
	Richard Shine
	Peter A White
**Order of Authors Secondary Information:**	
**Response to Reviewers:**	Please note that text within quotation marks is new or amended text taken directly from the revised manuscript.<br><br>1.Where I think the authors fall short is on not reporting any insights about (or derived from) the genome (aside from repeat content), despite having the first-hand look at it. It is a data note and therefore no requirements for biological analyses, but surely something can be said about the genes you have predicted? E.g. any gene families stand out? Are there genes in your genome draft that could explain, at least partially, the enormous success this species has in non-native environments? For instance, what is known about the gene(s) involved in the production of the toxic secretions you mentioned? Are there any clues from the resources you are sharing with the community?<br><br>We have had an incredible amount of interest from researchers with requests for access to this genome assembly for further biological studies. Thus, the main driving factor for submission was to make our data publicly available for more detailed analysis by the scientific community. Given that this is a data note, we believe that comprehensive biological analyses are best suited for follow-up publications. Further analysis will delay publication and prevent sharing of this highly petitioned dataset. However, we have released the genome in a WebApollo genome browser and included additional analysis of the predicted proteins, which will hope will encourage community annotation and curation of the genome which will aid such biological analyses (lines 286-291):<br><br>"Future work will be needed to improve the quality of gene annotation. We have included all of the MAKER2 predictions in our annotation and a full table of protein statistics and top blastp hits from this analysis for further biological analyses (Table S3). Annotation has also been made available via a WebApollo [53] genome browser (http://edwapollo.babs.unsw.edu.au/) and an associated search tool (http://www.slimsuite.unsw.edu.au/servers/apollo.php). This will facilitate community curation and annotation of genes of interest."<br><br>2.I look at the supporting data available on the FTP server and everything checks out. I do have a recommendation for an additional file (see below).<br><br>We have added a high-confidence gene set as recommended (see point 4, below).<br><br>3.The authors claim that the draft genome "sets a milestone in the field of anuran genetics". I would like the authors to describe why it is so, in their conclusion.

Powered by Editorial Manager® and ProduXion Manager® from Aries Systems Corporation

Both reviewer 1 and 3 have made a comment regarding the use of the word “milestone”. This has been omitted from the sentence.

4.Typically, for genome papers, a high-confidence gene set is also reported/provided (in addition to what is presented, often based on AED and other criteria). A high confidence set would be a very useful resource to have, reduce the gene space in the process and present a more focused, gold standard list of better-annotated genes. This set stands a higher chance of yielding valuable and meaningful insights for your and future studies, set that would hold against scientific scrutiny (In the process weeding out the many, potentially spurious, gene predictions reported herein).

We have created a high confidence gene set as recommended by the reviewer. This is based on MAKER2 Annotation Edit Distance (AED) and reciprocal high coverage BLAST hits to reference proteomes. We have also generated a table (Supplementary Table S4) with additional supporting data for each predicted gene to make it easier for users to identify subsets that meet confidence criteria appropriate to their own goals. The high confidence gene set has been uploaded to GigaDB (lines 291-294):

"For researchers who would like to use cane toad proteins in general evolutionary analyses, we have also created a "high quality" dataset of 6,580 protein-coding genes with an AED no greater than 0.25 and at least 90% reciprocal coverage of its top QFO blastp hit, excluding possible viral and transposon proteins, available from the GigaScience database."

As an additional resource, we have generated predicted orthologue multiple sequence alignments and maximum likelihood trees for this high quality proteins, which have also been uploaded to GigaDB. A phylogenetic supertree (Figure 8 & Figure S1) has been constructed from these trees and replaced the tree in Fig 1B, which had been made from published data. This is described in a new results section, “Phylogenetic analysis of high quality proteins” (lines 295-310):

"To further validate the high-quality protein data set, GOPHER [54] v3.4.2 was used to predict orthologues for each protein. QFO (04/18) [52] eukaryotic reference proteomes were supplemented with Uniprot Reference proteomes for Lithobates catesbeiana (UP000228934) [14] and Xenopus laevis (UP000186698) [17] and the annotated protein sequences of Nanorana parkeri v2 [15]. GOPHER orthologues were predicted with default settings based on a modified mutual best hit algorithm that accounts for one-to-many or many-to-many orthologous relationships and retains the closest orthologue from each species. The closest orthologues were aligned with MAFFT [55] v7.310 (default settings) and phylogenetic trees inferred with IQ-TREE [56] v1.6.1 (default settings) for alignments containing at least three sequences. Phylogenetic trees were inferred in this manner for 6,417 of the 6,580 high quality proteins. A supertree was then constructed from the 6,417 individual protein trees using CLANN [57] v4.2.2 (DFIT Most Similar Supertree Algorithm) (Figure 8, Figure S1). Branch consistency was calculated for each branch as the proportion of source trees with taxa either side of the branch that have no conflicts in terms of the placement of those taxa. The supertree supports the known phylogeny for amphibians used in this study, giving additional confidence in the quality and utility of these protein annotations. All alignments and trees are available in supplementary data via the GigaScience database."

5.The sentence on line 240 starting with "Critically.." is not accurate and needs to be re-worked. FYI some short read assemblers are able to assemble through repeats larger than read length with the help of paired-end information. You could rephrase to something like "The average length (XX +/- Std. dev.) of most (XX%) of these repeat classes exceeds that of the Illumina reads used in our study (Paired-end 150bp), making the short read assembly difficult in these regions. This is reflected by the low assembly contiguity (contig N50 length = 583bp)."

We agree with the reviewer that this sentence over-states the problems presented to short read assembly and have rephrased that sentence (now lines 316-319):

Powered by Editorial Manager® and ProduXion Manager® from Aries Systems Corporation

"The mean repeat length is 406 bp, which exceeds the Illumina read length used in our study (mean 140.6 bp paired-end). This makes short-read assembly of these regions difficult, as reflected by the poor ABySS contiguity (contig N50 = 583 bp, Table 2), and emphasises the need for long read data in this organism."

6.Though I must say that such a low contiguity figure is very untypical for an ABySS assembly, even for a highly repeated genome. Especially since your library captures sizes as long as 800bp. I am concerned about gDNA content/representation, as you seem to only have constructed a single paired-end library. Building multiple libraries from the same tissue source, preferably from 2 or more samples, prevent possible sampling/lab manipulation biases and ensures you have captured the entire genomic content. I also recommend building libraries of various insert sizes: 500, 2kbp, 5kbp whenever possible, especially when it is your only source of long-range information for assembly. This helps short read assemblers resolve repeats and increase the contiguity of the resulting assembly. Since you mainly used the ABySS short-read assembly for improving the accuracy of the DBG2OLC long read one, it might be ok in this case (especially since you recover many complete BUSCOs), but it also explains why a hybrid assembly approach does not improve the N50 length metric of the long-read DBG2OLC assembly - where I think it should.

We agree with the reviewer that the ABySS assembly is not as good as one might expect given its performance in other species. This was reflected by comparatively poor performance by other short read assembly attempts. We have had much better success using the same library preparation, PE strategy and ABySS assembly in other species. We think that the difficulties we've experienced whilst attempting to assemble the genome from short read data is most probably related to its high repeat content (see point 5, above). We acknowledge that it could also be influenced by gDNA representation, although the high BUSCO coverage gives us confidence of good coverage.

We agree that multiple insert sizes have improved the short-read assembly. However, we decided that generating more long read data was more useful. The reviewer is correct that we “mainly used the ABySS short-read assembly for improving the accuracy of the DBG2OLC long read one”. We acknowledge that this is not a final, complete cane toad genome, and trust that future sequencing efforts will be able to improve upon our assembly. Despite this, the draft genome in its current state will be enormously useful to the community.

7.The cane toad reference transcriptome was published by the Authors and used as direct evidence for MAKER gene prediction. The Authors briefly mentioned it as a "multi-tissue" from tadpoles and adults. It would be good to provide more information (2-3 sentences) on this evidence in the present study (so readers readily know what went in the gene prediction tools), especially if that information could be used to gain insights on cane toad genetics.

The following sentence has been added to the MS (lines 211-213).

"Whole-tadpoles and the brain, liver, spleen, muscle, ovary and testes of adult toads from Australia and Brazil were used to prepare cDNA libraries for the multi-tissue transcriptome sequencing."

8.line 219, typo, should read "Approximately"

This has been fixed in the manuscript.

9.Make sure you report to single digit (or double) consistently, throughout.

Table 2 has been fixed to give all percentage values to 1 d.p. Elsewhere, we have tried to consistently use the number of significant figures or decimal places that we consider to be appropriate for given values.

Powered by Editorial Manager® and ProduXion Manager® from Aries Systems Corporation

Reviewer #2

1.Although BUCSO analysis can be used for genome completeness, it is based on protein coding genes, so I think 'Assessment of genome completeness' would be better to be merged with 'Genome annotation and gene prediction' section.

We respectfully disagree with the reviewer on this point. BUSCO is a set of software and data for “assessing genome assembly and annotation completeness with single-copy orthologs”. An explicit objective of the tool is assessing genome completeness. It is also an important part of our manuscript’s narrative that our draft genome is capturing the majority of protein-coding regions well, despite being quite fragmented when contig statistics alone are considered. However, we acknowledge that BUSCO can also be used to assess annotation completeness and have added the BUSCO short score for the MAKER2 gene set to the 'Genome annotation and gene prediction’ section, along with more discussion of observed differences (see point 2, below).

2.In previous publication with R. marina transcriptome (Richardson, et al., GigaScience, 2018; doi: 10.1093/gigascience/gix114; Ref #18 on current manuscript), it was reported that 1.7% of BUCSO genes were fragmented, and 7.4% of them were missing on their 62,202 CDS transcripts. These numbers look better than genome-based result described in this manuscript (7.5% of fragmented, and 9.5% of missing). Authors may need to discuss the difference among these two annotations.

These differences are consistent with results from the BUSCO manuscript, in which it states: “Nevertheless, the fact that some genome assemblies appear less complete than their corresponding gene sets (e.g. H. sapiens Table 1) reveals limitations of the BUSCO gene prediction step.” … “Thus, it should be noted that while BUSCO assessments aim to robustly estimate completeness of the datasets, technical limitations (particularly gene prediction) may inflate proportions of ‘fragmented’ and ‘missing’ BUSCOs, especially for large genomes.” Deeper analysis of our BUSCO results to confirm this have now been included in the discussion of BUSCO results.

Lines 153-158: "It should be noted that these numbers mask some underlying complexity of BUSCO assessments; aggregate improvements in BUSCO scores with polishing include some losses as well as gains. Taking the best rating for each BUSCO in v2.0, v2.1 or v2.2 reduces the number of missing BUSCO genes to 326 (8.3%) and increases the complete number to 3366 (85.2%) (Figure 3, “R. marina (combined)”). This is explored further in the “Genome annotation and prediction” section, below."

Lines 270-285: "We ran BUSCO v2.0.1 (short mode, lineage tetrapoda_odb9, BLAST+ v2.2.31 [28], HMMer v3.1b2 [29], AUGUSTUS v3.2.2 [30], EMBOSS v6.5.7 [31]) on the MAKER2 transcriptome and proteome and retained the most complete rating for each gene (Figure 7A, Table S2, "Annotation"). MAKER annotation had fewer missing BUSCO genes than the v2.2 assembly (314 vs 375) but many more fragmented (561 vs 296). Equivalent BUSCO analysis of the Richardson et al. transcriptome [18] was only missing 296 genes. However, as seen with the assembly versions, these values mask hidden complexity. Combined BUSCO analysis of our hybrid assembly (v2.0, v2.1, v2.2) and annotation, revealed only 181 missing genes (Figure 7A, Table S2, “GigaDB”). Furthermore, >50% of the 279 genes “Missing” in the transcriptome are found in the genome and/or its annotation (Figure 7B, Table S2). When the transcriptome and our genome are combined, only 68 BUSCO genes (1.7%) are “Missing” and 3845 (97.3%) are “Complete” (Figure 7B, Table S2, “CaneToad”). This highlights the usefulness of our assembly, and illustrates the complementary nature of genome and transcriptome data: the former is more comprehensive but more difficult to assemble and annotate, whereas the latter is easier to assemble into full-length coding sequences but will miss some tissue-specific and lowly expressed genes. Some of the remaining "Missing" BUSCO genes may be present but too fragmented to reach the score threshold."

Powered by Editorial Manager® and ProduXion Manager® from Aries Systems Corporation

3.The analysis of 'unknown function' genes with published de novo transcriptome (p.9 line 229-) seems to have a circularity. Authors used all RNA-seq data already on their annotation, which are also used for de novo transcriptome construction (p.9 line 206). So instead of analyzing their matched length, I recommend to analyze their expression level from RNA-seq data. If 'unknown function' genes were mis-annotated genes as authors thought, it should have lower level of evidence for expression, compared to 'known function' genes.

We disagree with the reviewer that there is circularity in our argument. The same RNA-Seq data was used for prediction of both annotated and “unknown function” genes, so there is no reason for any difference in how well different subsets of predicted genes map to the transcriptome. (The transcriptome data was not pre-filtered at any step based on annotation.) Nevertheless, we agree that it is useful to look at expression levels. This has been incorporated into our extended analysis of the predicted genes (lines 247-254):

"We also reanalysed the multi-tissue RNA-Seq data from Richardson et al. [18] by mapping the reads onto the MAKER predicted transcripts. Filtered reads (adaptor sequences and reads with avg. Phred < 30 removed) were mapped with Salmon v0.8.0 [51] (Quasi-mapping default settings, IU libtype parameter). Read counts were converted into transcripts per million (TPM) by normalising by transcript length, dividing by the sum of the length-normalised read counts, and then multiplying by one million. We observed lower expression levels overall in the “unknown” set (Figure 6). With the caveat that real proteins may have very low expression, this is also consistent with the “unknown” gene set containing false annotations."

4.'3 s.f' (significant figure) notation on table headers make the reader confused. It is obvious to recognize by looking at numbers on table, so it would be better to remove it.

Reviewer 3 disagrees with this reviewer and has asked to place the shorthand ‘s.f.’ in the abbreviation list. We have kept ‘s.f.’ in the manuscript.

5.In Table 4, qPCR value is also the average of two experiments (p.8, line 190-191), so it would be fair to present min/max values for that.

These have now been included in the main text (line 193-195):

"Genome sizes were generated from the formulae outlined by [41] and the average of two estimates (2.81 Gb and 1.94 Gb) were used to obtain a genome size of 2.38 Gb."

Reviewer #3

1.p. 11; line 226: The authors identified 32,456 genes with unknown function in addition to the 25,846 predicted genes. The number of these unknown genes seem to much more than expected, but their explanation for it is insufficient. They mentioned that the median length is 171 aa, but what is the cut-off length of amino acids, and what is their range (the minimum and maximum)? In which regions in the genome sequence are those genes located? That is, are those genes scattered in the unique sequence in the genome or localized in the regions with repetitive sequences, transposable elements, or some other specific sequences? If the authors use the same strategy of pipelines for gene annotation with the X. laevis and X. tropicalis genome sequences, how many genes with unknown function could be identified and what percentage of them could be orthologous to those of R. marina?

We have expanded our analysis of the predicted genes, including analysis on the number of genes with of unknown function which have homologues in the Xenopus tropicalis reference proteome. (See also responses to Reviewer 1 (point 4), and Reviewer 2 (points 2 and 3).

Powered by Editorial Manager® and ProduXion Manager® from Aries Systems Corporation

Lines 235-242: "Further review of the predicted protein descriptions revealed 4,357 with likely origins in transposable elements (including 4,114 LINE-1 ORFs) and 215 from viruses, however many of these may be bona fide functional members of the cane toad proteome.

Poor quality protein predictions are generally shorter (generated from fragmented or random ORFs) and have a larger Annotation Edit Distance (AED) when compared to real proteins. Consistent with this, the predicted proteins of unknown function are shorter in sequence (median length 171 aa) to those with Swissprot hits (median length 388 aa) (Figure 5A) and have a greater AED (median 0.37 versus 0.2) (Figure 5B)."

Lines 255-269: "To investigate the role of fragmented ORFs, we downloaded the Quest For Orthologues (QFO) reference proteomes (QFO 04/18) [52] and used BLAST+ v2.2.31 [28] blastp (e-value < 10-7) to identify the top hit for each predicted protein in (a) all eukaryote reference proteomes, and (b) the Xenopus tropicalis reference proteome. BLAST results were converted into global coverage with GABLAM v2.28.3 [50]. As expected, the vast majority (99.6%) of “similar” proteins had a blastp hit the QFO proteomes (data not shown). Perhaps surprisingly, nearly two thirds (66.5%) of “unknown” proteins also had a blastp hit, but these had lower coverage of the reference proteins than did proteins in the “similar” class (data not shown). A “combined coverage” score was calculated for each protein, taking the minimum percentage coverage of either the query protein or its top QFO hit. This metric was related to annotation quality, showing an inverse relationship with AED (data not shown). Excluding proteins with annotation indicating possible viral or transposable element origin, 45.7% of "similar" proteins and 96.8% of “unknown” proteins had the same closest X. tropicalis blastp hit as another predicted protein. Consistent with this being related to gene fragmentation, there was a negative relationship between the number of cane toad proteins sharing a given X. tropicalis top hit, and how much of the X. tropicalis hit was covered by each cane toad protein."

Re-annotation of the Xenopus genomes would be a major undertaking and is beyond the scope of this paper.

2.Figure 5: The authors need to compare the data in Figure 5 with those of other amphibian species.

We agree with the reviewer that such a comparison would be interesting, but disagree that it is necessary. We are currently unable to generate the required data with sufficient rigor to be confident of a fair comparison and this is not the direct focus of the Data Note.

3.Is Rhinella marina the same as Rhinella marinus and Bufo marinus? The authors need to describe this in the abstract and introduction for clarification.

They are the same organism. Bufo marinus is an old scientific descriptor and has been replaced with Rhinella marina. This has been clarified in the abstract (line 52) and the introduction (line 76).

4.The genome size usually means the size of haploid DNA, but, in the text and table, the authors mentioned "a haploid genome size." When the authors simply use "the genome size," does this mean "a haploid genome size?" If so, better not to use "a haploid genome size."

“haploid genome size” has been changed to “genome size” throughout the manuscript.

5.p. 10, line 179: If PCR conditions are nothing special, those could be written in the legend of Tables or Figures, or deposited to "protocol.io."

Powered by Editorial Manager® and ProduXion Manager® from Aries Systems Corporation

PCR conditions have been moved to the legend of Table S1.

6.The authors should include s.f. and other abbreviations, if any, that are not listed, in the list of abbreviations.

AED, BLAST, HMM, lncRNA, ORF, QFO, TE, TPM, and s.f. have been added to the abbreviations list.

Reviewer #4

1.The authors take a hybrid assembly approach and mix a single sized 350 bp fragment Illumina library with larger fragment PacBio libraries. They extracted DNA from liver from an adult female. Liver is known to endoreduplicate, which can create rearrangements and problems for de novo assembly projects. However, BUSCO analysis indicates that many of the single genes have been identified in the assembly and their results are comparable to X. tropicalis, arguably the most well assembled and annotated amphibian genome available.

We agree with the reviewer that the BUSCO analysis is sound and that our results compare well with X. tropicalis and we hope to improve our assembly in the near future by sequencing of variety of tissue types. See also response to reviewer 1, point 6.

2.They used ABySS to assemble the genome but given that this genome note format is highly technical, it might be useful to report comparisons with other assemblers that they no doubt tried and/or provide more explanation for using ABySS relative to other assemblers.

We believe such comparisons are more appropriate for a technical note than a data note. The reviewer is correct that multiple assemblies and options were tried. However, we do not feel confident that we can use these data to provide robust technical insight.

3.Regarding their metrics in Table 2. I was confused by the %N reporting for their assembly and long read libraries. The authors report 0.0% of the assembly is in gaps, which is surprising given how repetitive amphibian genomes are, how poorly assembled the toad genome is (though comparably poor to other amphibians which have Ns) and nearly all vertebrate genome assemblies (including the human genome) have some bases unresolved and/or in gaps marked by a series of Ns. The proportion in gaps is an important metric of assembly quality. If the genome really does not have any Ns, it might be useful to highlight this unique attribute somewhere in the text and provide some explanation for how they were able to eliminate gaps.

The hybrid assembly produced is primarily error-corrected long read contigs, not scaffolds. As such, the lack of Ns represents an inability of the hybrid assembler to scaffold the contigs using the ABySS assembly (see reviewer 1, point 6), rather than gap elimination.

4.Their k-mer genome size estimation analysis shows the effect of kmer size and quality trimming but remains far from the estimated genome size based on flow cytometry and other experiments. The authors follow this up with a nice qPCR experiment and provide explanation for how far they are off. Given that the genome assembly size deviates substantially from the reported size, I would worry about using this assembly to analyze repeat content (as the authors state in the manuscript).

We agree with the reviewer's reservations. We report the repeat content of the assembly, not the genome. We explicitly do not claim that this assembly is a final and completely accurate representation of the true genome sequence. We draw attention to the difficulty that the repeats present for accurate assembly.

Powered by Editorial Manager® and ProduXion Manager® from Aries Systems Corporation

	5.As an additional confirmatory experiment to help build confidence in their results, I wonder if a synteny analysis with Xenopus tropicalis would be useful. Such a comparison might help reveal more about overall synteny and/or continuity and further strengthen their assembly results.<br><br>This is a great idea but beyond the scope of this paper.<br><br><br>6.Line 193-199: Here there is discussion about first estimate of genome size using either k-mer or qPCR analysis. This is not the first genome size estimate based on kmer distributions. Perhaps the authors want to state that this is the first amphibian genome estimated in this way? Maybe downplaying this sentence, or more clearly defining what they want to say here would be useful.<br><br>This sentence has been modified as per the reviewer’s suggestion for better clarity (lines 199-200):<br><br>"Given this is the first estimate of the cane toad genome size using either k-mer or qPCR analysis, …"<br><br><br>7.There are a number of sentences in the text that oversell the results a bit and these should be corrected (for example: line 54-55---consider eliminating the line about iconic status and major gaps in understanding cane toad genetics…..this is the case for nearly all organisms; line 248---the fragmented draft assembly, early stage protein-coding annotation results, and estimates that deviate from expectation is contributing to additional fragmented amphibian assemblies; a milestone should go further than what is reported in the manuscript).<br><br>Line 54-55: in our opinion this is not over-selling. No results have been presented and only facts are stated.<br><br>Line 248 (now line 326): we agree with the reviewer and the sentence has been modified to remove ‘milestone’.<br><br><br>8.The authors use MAKER2 for their gene annotation pipeline combined with their reference transcriptome. Given their abundant RNA-Seq, I was surprised that they did not use BRAKER1, which typically provides superior annotations compared to MAKER2. This might explain why it appears they have highly over-predicted the number of genes in the toad genome, though it could also stem from poor assembly. MAKER is widely used but their abundant RNA-Seq data is perfect for using BRAKER1 and they may obtain superior annotations using this tool.<br><br>We did consider BRAKER1 during the annotation phase. It is our understanding that later releases of Maker perform just as well as BRAKER1, with the additional benefit of repeat masking and protein alignments which BRAKER does not generate. We hope that making the data freely available, others will be able to improve on the annotations in time. To aid with this endeavour, we have also released the genome in a WebApollo genome browser, as pointed out in the response to point 1 by reviewer 1.<br><br><br>9.In some locations of the text, genus and species are italicized, in other locations they are not. Fix according to journal format requirements.<br><br>This has been fixed throughout the manuscript.
**Additional Information:**	
**Question**	**Response**
Are you submitting this manuscript to a special series or article collection?	No

Powered by Editorial Manager® and ProduXion Manager® from Aries Systems Corporation

**Experimental design and statistics**<br><br>Full details of the experimental design and statistical methods used should be given in the Methods section, as detailed in our Minimum Standards Reporting Checklist. Information essential to interpreting the data presented should be made available in the figure legends.<br><br>Have you included all the information requested in your manuscript?	Yes
**Resources**<br><br>A description of all resources used, including antibodies, cell lines, animals and software tools, with enough information to allow them to be uniquely identified, should be included in the Methods section. Authors are strongly encouraged to cite Research Resource Identifiers (RRIDs) for antibodies, model organisms and tools, where possible.<br><br>Have you included the information requested as detailed in our Minimum Standards Reporting Checklist?	Yes
**Availability of data and materials**<br><br>All datasets and code on which the conclusions of the paper rely must be either included in your submission or deposited in publicly available repositories (where available and ethically appropriate), referencing such data using a unique identifier in the references and in the “Availability of Data and Materials” section of your manuscript.<br><br>Have you have met the above requirement as detailed in our Minimum Standards Reporting Checklist?	Yes

Manuscript

Click here to access/download;Manuscript;CaneToadGenomePaper.2018-

Click here to view linked References

Draft genome assembly of the invasive cane toad, *Rhinella marina*

Richard J Edwards[1], Daniel Enosi Tuipulotu[1†], Timothy G Amos[1†], Denis O'Meally[2], Mark F Richardson[3,4], Tonia L Russell[5], Marcelo Vallinoto[6,7], Miguel Carneiro[6], Nuno Ferrand[6,8,9], Marc R Wilkins[1,5], Fernando Sequeira[6], Lee A Rollins[3,10], Edward C Holmes[11], Richard Shine[12] and Peter A White[1,*]

[†]These authors contributed equally to the work.

[1]School of Biotechnology and Biomolecular Sciences, University of New South Wales, Sydney, NSW, Australia

[2]Sydney School of Veterinary Science, Faculty of Science, University of Sydney, Camperdown, New South Wales, Australia

[3]School of Life and Environmental Sciences, Centre for Integrative Ecology, Deakin University, Geelong, VIC, Australia

[4]Bioinformatics Core Research Group, Deakin University, Geelong, VIC, Australia

[5]Ramaciotti Centre for Genomics, University of New South Wales, Sydney, NSW, Australia

[6]CIBIO/*InBIO*, Centro de Investigação em Biodiversidade e Recursos Genéticos, Universidade do Porto, Vairão, Portugal

[7]Laboratório de Evolução, Instituto de Estudos Costeiros (IECOS), Universidade Federal do Pará, Bragança, Pará, Brazil

[8]Departamento de Biologia, Faculdade de Ciências, Universidade do Porto, Porto, Portugal

[9]Department of Zoology, Faculty of Sciences, University of Johannesburg, Auckland Park, South Africa

[10]Evolution and Ecology Research Centre, School of Biological Earth and Environmental Sciences, University of New South Wales, Sydney, NSW, Australia

[11]Marie Bashir Institute for Infectious Diseases and Biosecurity, Charles Perkins Centre, School of Life and Environmental Sciences and Sydney Medical School, University of Sydney, Sydney, NSW, Australia

[12]School of Life and Environmental Sciences, Faculty of Science, University of Sydney, Camperdown, New South Wales, Australia

Emails of all authors: richard.edwards@unsw.edu.au (RJE), d.enosi@unsw.edu.au (DET), t.amos@unsw.edu.au (TGA), omeally@gmail.com (DO), m.richardson@deakin.edu.au (MFR), t.russell@unsw.edu.au (TLR), mvallinoto@cibio.up.pt (MV), miguel.carneiro@cibio.up.pt (MC), nferrand@cibio.up.pt (NF), m.wilkins@unsw.edu.au (MRW), fsequeira@cibio.up.pt (FS), l.rollins@unsw.edu.au (LAR), edward.holmes@sydney.edu.au (ECH), rick.shine@sydney.edu.au (RS), p.white@unsw.edu.au (PAW).

*Corresponding author address: School of Biotechnology and Biomolecular Sciences, University of New South Wales, Sydney, NSW, Australia. Tel: +61-293853780; Email: p.white@unsw.edu.au

Abstract

Background: The cane toad (*Rhinella marina* formerly *Bufo marinus*) is a species native to Central and South America that has spread across many regions of the globe. Cane toads are known for their rapid adaptation and deleterious impacts on native fauna in invaded regions. However, despite an iconic status, there are major gaps in our understanding of cane toad genetics. The availability of a genome would help to close these gaps and accelerate cane toad research. **Findings:** We report a draft genome assembly for *R. marina*, the first of its kind for the Bufonidae family. We used a combination of long read PacBio RS II and short read Illumina HiSeq X sequencing to generate a total of 359.5 Gb of raw sequence data. The final hybrid assembly of 31,392 scaffolds was 2.55 Gb in length with a scaffold N50 of 168 kb. BUSCO analysis revealed that the assembly included full length or partial fragments of 90.6% of tetrapod universal single-copy orthologs (n=3950), illustrating that the gene-containing regions have been well-assembled. Annotation predicted 58,302 protein coding genes, with 25,846 similar to known proteins in SwissProt. Repeat sequences were estimated to account for 63.9% of the assembly. **Conclusion:** The *R. marina* draft genome assembly will be an invaluable resource that can be used to further probe the biology of this invasive species. Future analysis of the genome will provide insights into cane toad evolution and enrich our understanding of their interplay with the ecosystem at large.

Keywords: cane toad; *Rhinella marina;* sequencing; hybrid assembly; genome; annotation

Data Description

Introduction

The cane toad (*Rhinella marina* formerly *Bufo marinus*) (Figure 1) is a true toad (Bufonidae) native to Central and South America that has been introduced to many areas across the globe [1]. Since its introduction into Queensland in 1935, the cane toad has spread widely and now occupies more than 1.2 million square kilometres of the Australian continent, fatally poisoning predators like the northern quoll, freshwater crocodiles, and several species of native lizards and snakes [1-5]. The ability of cane toads to kill predators with toxic secretions has contributed to the success of their invasion [1]. To date, research on cane toads has focused primarily on ecological impacts, rapid evolution of phenotypic traits, and population genetics using neutral markers [6, 7], with limited knowledge of the genetic changes that allow the cane toad to thrive in the Australian environment [8-11]. A reference genome will be useful for studying loci subject to rapid evolution and could provide valuable insights into how invasive species adapt to new environments. Amphibian genomes have a preponderance of repetitive DNA [12, 13], confounding assembly with the limited read lengths of first- and second-generation sequencing technologies. Here, we employ a hybrid assembly of PacBio long reads and Illumina short reads (Figure 2) to overcome assembly challenges presented by the repetitive nature of the cane toad genome. Using this approach, we assembled a draft genome of *R. marina* that is comparable in contiguity and completeness to other published anuran genomes [14-17]. We used our previously published transcriptomic data [18] and other published anuran sequences to annotate the genome. Our draft cane toad assembly will serve as a reference for genetic and evolutionary studies, and provides a template for continued refinement with additional sequencing efforts.

Sample collection, library construction and sequencing

Adult female cane toads were collected by hand from Forrest River in Oombulgurri, WA (15.1818°S, 127.8413°E) in June 2015. Toads were placed in individual damp cloth bags and transported by plane to Sydney, NSW before they were anaesthetised by refrigeration for four hours and killed by subsequent freezing. High-molecular weight genomic DNA (gDNA) was extracted from the liver of a single female

using the genomic-tip 100/G kit (Qiagen, Hilden, Germany). This was performed with supplemental RNase (Astral Scientific, Taren Point, Australia) and proteinase K (NEB, Ipswich, MA, USA) treatment, as per the manufacturer's instructions. Isolated genomic DNA was further purified using AMPure XP beads (Beckman Coulter, Brea, CA, USA) to eliminate sequencing inhibitors. DNA quantity was assessed using the Quanti-iT PicoGreen dsDNA kit (Thermo Fisher Scientific, Waltham, MA, USA), DNA purity was calculated using a Nanodrop spectrophotometer (Thermo Fisher Scientific), and molecular integrity assessed by pulse-field gel electrophoresis.

For short read sequencing, a paired-end library was constructed from the gDNA using the TruSeq PCR-free library preparation kit (Illumina, San Diego, CA, USA). Insert sizes ranged between 200-800 bp. This library was sequenced (2 × 150 bp) on the HiSeq X Ten platform (Illumina) to generate approximately 282.9 Gb of raw data (Table 1). Illumina short sequencing reads were assessed for quality using FastQC v0.10.1 [19]. Low quality reads filtered were trimmed using Trimmomatic v0.36 [20] with a Q30 threshold (LEADING:30, TRAILING:30, SLIDINGWINDOW:4:30) and a minimum 100 bp read length, leaving 64.9% of the reads generated, of which 75.2% were in retained read pairs.

For long read sequencing, we utilised the single-molecule real time (SMRT) sequencing technology (Pacific Biosciences, Menlo Park, CA, USA). Four SMRTbell libraries were prepared from gDNA using the SMRTBell template preparation kit 1.0 (Pacific Biosciences). To increase subread length, either 15-50 kb or 20-50 kb BluePippin size selection (Sage Science, Beverly, MA, USA) was performed on each library. Recovered fragments were sequenced using P6C4 sequencing chemistry on the RS II platform (240 min movie time). The four SMRTbell libraries were sequenced on a total of 97 SMRT cells to generate 7,745,233 subreads for a total of 76.6 Gb of raw data. Collectively, short and long read sequencing produced around 359.5 Gb of data (Table 1).

Genome assembly

We employed a hybrid *de novo* whole genome assembly strategy, combining both short read and long read data. Trimmed Q30-filtered short reads were *de novo* assembled with ABySS v1.3.6 [21] using k=64 and default parameters (contig N50 = 583 bp) (Table 2). Long sequence reads were *de novo*

assembled using the program DBG2OLC [22] (k 17 AdaptiveTh 0.0001 KmerCovTh 2 MinOverlap 20 RemoveChimera 1) (contig N50 = 167.04 kbp) (Table 2). Following this, both assemblies were merged together using the hybrid assembler (‘sparc’) tool of DBG2OLC with default parameters, combining the contiguity of the long read data with the improved accuracy of the high coverage Illumina assembly. This hybrid assembly (v2.0) was twice ‘polished’ to remove errors. In the first round, the Q30 trimmed Illumina reads were mapped to the hybrid assembly with bowtie v2.2.9 [23] and filtered for proper pairs using samtools v1.3.1 [24]. Scaffolds were polished with Pilon v1.21 [25] to generate the second iteration of the assembled genome (v2.1). In the second round, PacBio subreads were mapped to assembly v2.1 for error correction using SMRT analysis software (Pacific Biosciences): PacBio subreads for each library were converted to BAM format with bax2bam v0.0.08 and aligned to the genome using pbalign v.0.3.0. BAM alignment files were combined using samtools merge v1.3.1 and the scaffolds polished with Arrow v2.1.0 to generate the final genome assembly (v2.2). Our final draft assembly of the cane toad genome (v2.2) has 31,392 scaffolds with an N50 of 167 kb (Table 2). The GC content (43.23%) is within 1% of the published estimate of 44.17%, determined by flow cytometry [26].

Assessment of genome completeness

BUSCO [27] analysis of conserved single copy orthologues is widely used as a proxy for genome completeness and accuracy. While direct comparisons are only truly valid within an organism, comparing BUSCO scores to genomes from related organisms provides a useful benchmark. We ran BUSCO v2.0.1 (short mode, lineage tetrapoda_odb9, BLAST+ v2.2.31 [28], HMMer v3.1b2 [29], AUGUSTUS v3.2.2 [30], EMBOSS v6.5.7 [31]) on each of our assemblies, along with four published anuran genomes (Figure 3, Table 2). The hybrid assembly combined the completeness of the long read assembly with the accuracy of the short read assembly, providing an enormous boost in BUSCO completeness from less than 50% full and partial orthologs to over 90%. Error correction through pilon and arrow polishing had a positive effect on the BUSCO measurement of genome completeness, with an increase of 7.8% in the number of full and partial orthologs between v2.0 and 2.2. For the polished assembly (v2.2), 3279 (83.0%) of the 3950 ultra-conserved tetrapod genes were complete, 296 (7.5%)

were fragmentary and 375 (9.5%) were missing. It should be noted that these numbers mask some underlying complexity of BUSCO assessments; aggregate improvements in BUSCO scores with polishing include some losses as well as gains. Taking the best rating for each BUSCO in v2.0, v2.1 or v2.2 reduces the number of missing BUSCO genes to 326 (8.3%) and increases the complete number to 3366 (85.2%) (Figure 3, "*R. marina* (combined)"). This is explored further in the "Genome annotation and prediction" section, below. Overall, BUSCO metrics indicate that our draft *R. marina* genome is approaching the quality and completeness of the widely used anuran amphibian reference genomes for *X. laevis* (v9.2) [17] and *X. tropicalis* (v.9.1) [16] and compares well to the recently published neobatrachian genomes of *Nanorana parkeri* (v2) [15] and *Lithobates catesbeianus* (v2.1) [14].

Estimation of *R. marina* genome size

Previous reports have estimated the size of the cane toad genome from 3.98-5.65 Gb using either densitometry or flow cytometry analysis of stained nuclei within erythrocytes, hepatocytes and renal cells [26, 32-38]. We employed two alternative strategies to measure the genome size, using short read k-mer distributions and qPCR of single copy genes. K-mer frequencies were calculated for both raw and trimmed Q30-filtered paired-end short reads (Table 1) with Jellyfish v2.2.3 [39] using k=21 and k=23, and a maximum k-mer count of 10,000. K-mer distributions were analysed using GenomeScope [40] with mean read lengths of 148 bp (raw) or 141 bp (Q30) and k-mer coverage cut-offs of 1000 and 10,000 (Table 3, Figure 4). GenomeScope gave genome size estimates ranging from 1.77 Gb to 2.30 Gb with the raw reads giving consistently larger estimates (1.85 Gb to 2.30 Gb) than the trimmed and filtered reads (1.77 Gb to 2.10 Gb). Estimates of the unique (single copy) region of the genome were more consistent, ranging from 1.31 Gb to 1.46 Gb, with k=23 estimates 99 Mb (raw) or 80 Mb (Q30) higher than k=21. Increasing the GenomeScope maximum k-mer coverage threshold had the greatest effect on predicted genome size, increasing repeat length estimates by 274 Mb to 385 Mb. GenomeScope predictions are affected by non-uniform repeat distributions and this difference could indicate high copy number repeats in the genome that are difficult to model accurately. It is possible that high frequency repeats with raw sequencing counts exceeding 10,000 are resulting in an

underestimate of total repeat length and therefore genome size, compared to the previous densitometry and flow cytometry predictions.

In the second approach, the *zfp292* (zinc finger protein 292) gene was selected from our BUSCO analysis as a single-copy target for genome estimation by qPCR [41]. First, PCR was used to amplify a 326 bp region of *zfp292* (scaffold 6589, position 345,750-346,075) in a 25 µL reaction that contained 50 ng of gDNA, 200 µM dNTP, 0.625 units of Taq polymerase (Invitrogen), 10 × Taq polymerase buffer (Invitrogen) and 0.4 µM of each primer (Table S1). The amplicon was cloned into the pGEM-T Easy vector (Promega, Madison, WI, USA) and the resultant plasmid was linearised with NdeI before being serially diluted to generate a qPCR standard (10^1-10^9 copies/µL). To amplify a smaller region (120 bp) within *zfp292* (scaffold 6589, position 345,858-345,977) gDNA (10-25 ng) or 1 µL of the diluted standards were used as a template for a 20 µL qPCR reaction containing 2 × iTaq SYBR Green mastermix (BioRad, Hercules, CA, USA) and 0.5 µM of each primer (Table S1). Cycle threshold values obtained for each plasmid dilution were used to generate a standard curve and infer the number of *zfp292* amplicons generated from the template gDNA of known quantity. Genome sizes were generated from the formulae outlined by [41] and the average of two estimates (2.81 Gb and 1.94 Gb) were used to obtain a genome size of 2.38 Gb. This genome size provides an estimated combined 151X sequencing coverage (119X Illumina and 32X PacBio) (Table 4).

Our genome size estimation of 1.98 to 2.38 Gbp is smaller than the 2.55 Gbp assembly size, and differs significantly from previously published estimates of 4 Gbp or more for this species. We suggest this is a result of the repetitive nature of the genome (see below). Given this is the first estimate of the cane toad genome size using either k-mer or qPCR analysis, further investigations are required to more clearly understand the discrepancy in our estimates with respect to published genome sizes. Here we estimate the depth of sequencing coverage using both sequence-based and cytometric genome size measures (Table 4).

Genome annotation and gene prediction

Annotation of the draft genome was performed using MAKER2 v2.31.6 [42], BLAST+ v2.2.31 [28], AUGUSTUS v3.2.2 [30], Exonerate v2.2.0 [43], RepeatMasker v4.0.6 [44] (DFAM [45], Library Dfam_1.2; RMLibrary v20150807), RepeatModeler v1.0.8 [46] and SNAP v2013-11-29 [47] using all SwissProt protein sequences (downloaded 2017-02-23)[48] . AUGUSTUS was trained using BUSCO v2.0.1 (long mode, lineage tetrapoda_odb9) and a multi-tissue reference transcriptome we previously generated from tadpoles and six adult cane toad tissues [18] (available from GigaDB [49], Genbank accession PRJNA383966). Whole-tadpoles and the brain, liver, spleen, muscle, ovary and testes of adult toads from Australia and Brazil were used to prepare cDNA libraries for the multi-tissue transcriptome sequencing. After the initial training run, two further iterations of MAKER2 were run using HMMs from SNAP training created from the previous run. Functional annotation of protein-coding genes predicted by MAKER2 were generated using Interproscan 5.25-64.0, with the following settings: -dp -t p -pa -goterms -iprlookup -appl TIGRFAM, SFLD, Phobius, SUPERFAMILY, PANTHER, Gene3D, Hamap, ProSiteProfiles, Coils, SMART, CDD, PRINTS, ProSitePatterns, SignalP_EUK, Pfam, ProDom, MobiDBLite, PIRSF, TMHMM. BLAST+ v2.6.0 [28] was used to annotate predicted genes using all Swissprot proteins (release 2017_08, downloaded 2017-09-01) [48] using the following settings: -evalue 0.000001 -seg yes -soft_masking true -lcase_masking -max_hsps 1.

In total, 58,302 protein-coding genes were predicted by the MAKER pipeline with an average of 5.3 exons and 4.3 introns per gene (Table 5). Of these, 5,225 are single exon genes, giving 4.7 introns per multi-exon gene with an average intron length of 4.08 kb. Predicted coding sequences make up 2.38% of the assembly. MAKER predicted considerably more than the approximately twenty thousand genes expected for a typical vertebrate genome. There are two likely explanations for this: (1) artefactual duplications in the genome assembly, either through under-assembly or legitimate assembly of two heterozygous diploid copies; (2) over-prediction of proteins during genome annotation, including pseudogenes with high homology to functional genes, proteins from transposable elements or other repeats, and multiple fragments of open reading frames (ORFs) from the same gene (due to fragmentation of the genome) and lncRNA genes that have been incorrectly assigned a coding sequence.

Of the 3,279 complete BUSCO genes identified (Table 2), only 85 (2.59%) were duplicated. This suggests that there is not widespread duplication in the assembly. Only 25,846 predicted genes were annotated as similar to known proteins in SwissProt, with the remaining 32,456 predictions "of unknown function". This is consistent with over-prediction being the primary cause of inflated gene numbers. Further review of the predicted protein descriptions revealed 4,357 with likely origins in transposable elements (including 4,114 LINE-1 ORFs) and 215 from viruses, however many of these may be bona fide functional members of the cane toad proteome.

Poor quality protein predictions are generally shorter (generated from fragmented or random ORFs) and have a larger Annotation Edit Distance (AED) when compared to real proteins. Consistent with this, the predicted proteins of unknown function are shorter in sequence (median length 171 aa) to those with Swissprot hits (median length 388 aa) (Figure 5A) and have a greater AED (median 0.37 versus 0.2) (Figure 5B). To investigate this further, predicted transcript and protein sequences were searched against the published *de novo* assembled transcriptome [18] using BLAST+ v2.2.31 [28] blastn or tblastn (top 10 hits, e-value < 10^{-10}) and compiled with GABLAM v2.28.3 [50]. For 56.5% of proteins with functional annotation, 95%+ of the protein length mapped to the top transcript hit (Table 6). Only 27.1% of unknown proteins had 95%+ coverage in the top transcript hit, which is again consistent with over-prediction. We also reanalysed the multi-tissue RNA-Seq data from Richardson *et al.* [18] by mapping the reads onto the MAKER predicted transcripts. Filtered reads (adaptor sequences and reads with avg. Phred < 30 removed) were mapped with Salmon v0.8.0 [51] (Quasi-mapping default settings, IU libtype parameter). Read counts were converted into transcripts per million (TPM) by normalising by transcript length, dividing by the sum of the length-normalised read counts, and then multiplying by one million. We observed lower expression levels overall in the "unknown" set (Figure 6). With the caveat that real proteins may have very low expression, this is also consistent with the "unknown" gene set containing false annotations.

To investigate the role of fragmented ORFs, we downloaded the Quest For Orthologues (QFO) reference proteomes (QFO 04/18) [52] and used BLAST+ v2.2.31 [28] blastp (e-value < 10^{-7}) to identify the top hit for each predicted protein in (a) all eukaryote reference proteomes, and (b) the *Xenopus*

tropicalis reference proteome. BLAST results were converted into global coverage with GABLAM v2.28.3 [50]. As expected, the vast majority (99.6%) of "similar" proteins had a blastp hit the QFO proteomes (data not shown). Perhaps surprisingly, nearly two thirds (66.5%) of "unknown" proteins also had a blastp hit, but these had lower coverage of the reference proteins than did proteins in the "similar" class (data not shown). A "combined coverage" score was calculated for each protein, taking the minimum percentage coverage of either the query protein or its top QFO hit. This metric was related to annotation quality, showing an inverse relationship with AED (data not shown). Excluding proteins with annotation indicating possible viral or transposable element origin, 45.7% of "similar" proteins and 96.8% of "unknown" proteins had the same closest *X. tropicalis* blastp hit as another predicted protein. Consistent with this being related to gene fragmentation, there was a negative relationship between the number of cane toad proteins sharing a given *X. tropicalis* top hit, and how much of the *X. tropicalis* hit was covered by each cane toad protein.

We ran BUSCO v2.0.1 (short mode, lineage tetrapoda_odb9, BLAST+ v2.2.31 [28], HMMer v3.1b2 [29], AUGUSTUS v3.2.2 [30], EMBOSS v6.5.7 [31]) on the MAKER2 transcriptome and proteome and retained the most complete rating for each gene (Figure 7A, Table S2, "Annotation"). MAKER annotation had fewer missing BUSCO genes than the v2.2 assembly (314 vs 375) but many more fragmented (561 vs 296). Equivalent BUSCO analysis of the Richardson *et al.* transcriptome [18] was only missing 296 genes. However, as seen with the assembly versions, these values mask hidden complexity. Combined BUSCO analysis of our hybrid assembly (v2.0, v2.1, v2.2) and annotation, revealed only 181 missing genes (Figure 7A, Table S2, "GigaDB"). Furthermore, >50% of the 279 genes "Missing" in the transcriptome are found in the genome and/or its annotation (Figure 7B, Table S2). When the transcriptome and our genome are combined, only 68 BUSCO genes (1.7%) are "Missing" and 3845 (97.3%) are "Complete" (Figure 7B, Table S2, "CaneToad"). This highlights the usefulness of our assembly, and illustrates the complementary nature of genome and transcriptome data: the former is more comprehensive but more difficult to assemble and annotate, whereas the latter is easier to assemble into full-length coding sequences but will miss some tissue-specific and lowly

expressed genes. Some of the remaining "Missing" BUSCO genes may be present but too fragmented to reach the score threshold.

Future work will be needed to improve the quality of gene annotation. We have included all of the MAKER2 predictions in our annotation and a full table of protein statistics and top blastp hits from this analysis for further biological analyses (Table S3). Annotation has also been made available via a WebApollo [53] genome browser (http://edwapollo.babs.unsw.edu.au/) and an associated search tool (http://www.slimsuite.unsw.edu.au/servers/apollo.php). This will facilitate community curation and annotation of genes of interest. For researchers who would like to use cane toad proteins in general evolutionary analyses, we have also created a "high quality" dataset of 6,580 protein-coding genes with an AED no greater than 0.25 and at least 90% reciprocal coverage of its top QFO blastp hit, excluding possible viral and transposon proteins, available from the *GigaScience* database.

Phylogenetic analysis of high quality proteins

To further validate the high-quality protein data set, GOPHER [54] v3.4.2 was used to predict orthologues for each protein. QFO (04/18) [52] eukaryotic reference proteomes were supplemented with Uniprot Reference proteomes for *Lithobates catesbeiana* (UP000228934) [14] and *Xenopus laevis* (UP000186698) [17] and the annotated protein sequences of *Nanorana parkeri* v2 [15]. GOPHER orthologues were predicted with default settings based on a modified mutual best hit algorithm that accounts for one-to-many or many-to-many orthologous relationships and retains the closest orthologue from each species. The closest orthologues were aligned with MAFFT [55] v7.310 (default settings) and phylogenetic trees inferred with IQ-TREE [56] v1.6.1 (default settings) for alignments containing at least three sequences. Phylogenetic trees were inferred in this manner for 6,417 of the 6,580 high quality proteins. A supertree was then constructed from the 6,417 individual protein trees using CLANN [57] v4.2.2 (DFIT Most Similar Supertree Algorithm) (Figure 8, Figure S1). Branch consistency was calculated for each branch as the proportion of source trees with taxa either side of the branch that have no conflicts in terms of the placement of those taxa. The supertree supports the known phylogeny for amphibians used in this study, giving additional confidence in the quality and utility of these protein annotations. All alignments and trees are available in supplementary data via the *GigaScience* database.

Repeat identification and analysis

The cane toad genome has proven very difficult to assemble using short reads alone, which suggests a high frequency of repetitive sequences, as for other amphibians [12, 13]. RepeatMasker annotations from the MAKER pipeline support this interpretation, with over 4.1 million repeat sequences detected, accounting for 63.9% of the assembly (Table 5). The mean repeat length is 406 bp, which exceeds the Illumina read length used in our study (mean 140.6 bp paired-end). This makes short-read assembly of these regions difficult, as reflected by the poor ABySS contiguity (contig N50 = 583 bp, Table 2), and emphasises the need for long read data in this organism. The most abundant class of repeat elements are of unknown type (1.61 million elements covering 32.28% of the assembly), with DNA transposons the most abundant known class of element (817,262 repeats; 19.17% coverage). Of these, the most abundant are of the hAT-Ac (231,332 copies) and TcMar-Tc1 (226,145 copies) superfamilies (Table S4). Accounting for overlaps between repeat and gene features, 18.7% of the assembly (479,397,014 bp) has no annotation (Figure 9).

Conclusion

This draft genome assembly will be an invaluable tool for advancing knowledge of anuran biology, genetics and the evolution of invasive species. Furthermore, we envisage these data will facilitate the development of biocontrol strategies that reduce the impact of cane toads on native fauna.

Availability of supporting data

Raw genomic sequencing data (Illumina and PacBio) and assembled scaffolds have been deposited in the ENA with the study accession PRJEB24695 and assembly accession GCA_900303285. The genome assembly and annotation are also available in the *GigaScience* database, and via a WebApollo [53] genome browser and an associated search tool (http://www.slimsuite.unsw.edu.au/servers/apollo.php).

List of abbreviations

AED: annotation edit distance; BUSCO: Benchmarking Universal Single-Copy Orthologs; BLAST: Basic Local Alignment Search Tool, qPCR: quantitative polymerase chain reaction, HMM: hidden Markov model, CDS: coding sequence; bp: base pair; gDNA: genomic DNA; ORF: open reading frame; QFO: Quest For Orthologues; SMRT: single-molecule real time; SINE: short interspersed nuclear element; LINE: long interspersed nuclear element, LTR: long terminal repeat; TE: transposable elements; TPM: transcripts per million; UTR: untranslated region, s.f.: significant figure

Additional files

Figure S1. Phylogenetic supertree constructed from phylogenetic trees for 6,417 high confidence cane toad proteins.

Table S1. Primers used for genome size estimation by single copy gene qPCR.

Table S2. Individual and combined full BUSCO gene ratings for cane toad assemblies, annotation, transcriptome.

Table S3. Sequence statistics, top BLAST hits, and classification for MAKER2 annotations.

Table S4. RepeatMasker statistics broken down by repeat category.

Ethics approval and consent to participate

All experimentation was performed under the approval of the University of Sydney Animal Ethics Committee.

Consent for publication

Not applicable

Competing interests

The authors declare that they have no competing interests.

Funding

This work was supported by the Cane Toad Genome Consortium and Bioplatforms Australia. Funding was provided by the Australian Research Council (DP160102991). L.A.R. is funded by ARC DECRA Fellowship (DE150101393). R.S. is funded by ARC Australian Laureate Fellowship (FL120100074). E.C.H. is funded by ARC Australian Laureate Fellowship (FL170100022). M.R.W. and the Ramaciotti Centre for Genomics acknowledge infrastructure funding from the Australian Research Council (LE150100031), the Australian Government NCRIS scheme administered by Bioplatforms Australia and the New South Wales Government RAAP scheme. M.V. is supported by a CNPq fellowship (302892/2016-8). M.C. is supported by a FCT investigator grant (IF/00283/2014/CP1256/CT0012). F.S. is supported by FEDER (COMPETE, POCI-01-0145-FEDER-006821) and FCT (UID/BIA/50027/2013) funds. D.E.T. was supported through an Australian Government Research Training Program (RTP) scholarship.

Author's contributions

P.A.W coordinated the project. P.A.W, R.S, E.C.H, L.A.R, R.J.E, M.W. designed the study. P.A.W, R.S, E.C.H, L.A.R, R.J.E and F.S funded the project. R.S provided the cane toad samples. D.E.T performed the genomic DNA extraction, PCR experiments and data analysis. T.L.R performed the sequencing. R.J.E and T.G.A performed the genome assemblies and primary data analysis. D.O and T.G.A. performed the genome annotation. R.J.E, D.E.T, T.G.A and P.A.W and wrote the manuscript. All authors edited and approved the final manuscript.

Acknowledgements

We thank the Ramaciotti Centre for Genomics, UNSW and the Kinghorn Centre for Clinical Genomics at the Garvan Institute of Medical Research who performed the sequencing in this study. This project was undertaken with the assistance of resources and services from the National Computational Infrastructure (NCI), which is supported by the Australian Government. This project used computational resources from Intersect, which were in part supported by the Australian Research Council. The authors acknowledge UNSW Science's high performance computing cluster (Katana), the

UNSW School of Biotechnology and Biomolecular Sciences high memory node, the Sydney Informatics Hub and the University of Sydney's high performance computing cluster (Artemis) for providing the high performance computing resources that have contributed to the research results reported within this paper. We thank the members of the UNSW Science Faculty Computing unit, particularly Duncan Smith, for all his help getting software installed and running on the katana computer cluster. We thank Dr Andrew Kelly for early discussions on cane toad genome size and technical assistance with gDNA extractions.

References

1. Shine R. The ecological impact of invasive cane toads (Bufo marinus) in Australia. The Quarterly Review of Biology. 2010;85 3:253-91.
2. Phillips BL, Brown GP, Greenlees M, Webb JK and Shine R. Rapid expansion of the cane toad (Bufo marinus) invasion front in tropical Australia. Austral Ecology. 2007;32 2:169-76.
3. Phillips BL, Brown GP and Shine R. Assessing the potential impact of cane toads on Australian snakes. Conservation Biology. 2003;17 6:1738-47.
4. Smith JG and Phillips BL. Toxic tucker: the potential impact of cane toads on Australian reptiles. Pacific Conservation Biology. 2006;12 1:40-9.
5. Urban MC, Phillips BL, Skelly DK and Shine R. The cane toad's (Chaunus [Bufo] marinus) increasing ability to invade Australia is revealed by a dynamically updated range model. Proceedings of the Royal Society of London B: Biological Sciences. 2007;274 1616:1413-9.
6. Slade R and Moritz C. Phylogeography of Bufo marinus from its natural and introduced ranges. Proceedings of the Royal Society of London B: Biological Sciences. 1998;265 1398:769-77.
7. Sequeira F, Sodré D, Ferrand N, Bernardi JA, Sampaio I, Schneider H, et al. Hybridization and massive mtDNA unidirectional introgression between the closely related Neotropical toads Rhinella marina and R. schneideri inferred from mtDNA and nuclear markers. BMC evolutionary biology. 2011;11 1:264.
8. Rollins LA, Richardson MF and Shine R. A genetic perspective on rapid evolution in cane toads (Rhinella marina). Molecular Ecology. 2015;24 9:2264-76.
9. Estoup A, Baird SJ, Ray N, Currat M, CORNUET J, Santos F, et al. Combining genetic, historical and geographical data to reconstruct the dynamics of bioinvasions: application to the cane toad Bufo marinus. Molecular ecology resources. 2010;10 5:886-901.
10. Trumbo DR, Epstein B, Hohenlohe PA, Alford RA, Schwarzkopf L and Storfer A. Mixed population genomics support for the central marginal hypothesis across the invasive range of the cane toad (Rhinella marina) in Australia. Molecular ecology. 2016;25 17:4161-76.
11. Leblois R, Rousset F, Tikel D, Moritz C and Estoup A. Absence of evidence for isolation by distance in an expanding cane toad (Bufo marinus) population: an individual-based analysis of microsatellite genotypes. Molecular Ecology. 2000;9 11:1905-9.
12. Bozzoni I and Beccari E. Clustered and interspersed repetitive DNA sequences in four amphibian species with different genome size. Biochimica et Biophysica Acta (BBA)-Nucleic Acids and Protein Synthesis. 1978;520 2:245-52.
13. Olmo E. Genome variations in the transition from amphibians to reptiles. Journal of molecular evolution. 1991;33 1:68-75.
14. Hammond SA, Warren RL, Vandervalk BP, Kucuk E, Khan H, Gibb EA, et al. The North American bullfrog draft genome provides insight into hormonal regulation of long noncoding RNA. Nature Communications. 2017;8 1:1433.

15. Sun Y-B, Xiong Z-J, Xiang X-Y, Liu S-P, Zhou W-W, Tu X-L, et al. Whole-genome sequence of the Tibetan frog Nanorana parkeri and the comparative evolution of tetrapod genomes. Proceedings of the National Academy of Sciences. 2015;112 11:E1257-E62.
16. Hellsten U, Harland RM, Gilchrist MJ, Hendrix D, Jurka J, Kapitonov V, et al. The genome of the Western clawed frog Xenopus tropicalis. Science. 2010;328 5978:633-6.
17. Session AM, Uno Y, Kwon T, Chapman JA, Toyoda A, Takahashi S, et al. Genome evolution in the allotetraploid frog Xenopus laevis. Nature. 2016;538 7625:336.
18. Richardson MF, Sequeira F, Selechnik D, Carneiro M, Vallinoto M, Reid JG, et al. Improving amphibian genomic resources: a multi-tissue reference transcriptome of an iconic invader. GigaScience. 2017;7 1:1-7.
19. Andrews S. FastQC: a quality control tool for high throughput sequence data. 2010.
20. Bolger AM, Lohse M and Usadel B. Trimmomatic: a flexible trimmer for Illumina sequence data. Bioinformatics. 2014;30 15:2114-20.
21. Simpson JT, Wong K, Jackman SD, Schein JE, Jones SJ and Birol I. ABySS: a parallel assembler for short read sequence data. Genome research. 2009;19 6:1117-23.
22. Ye C, Hill CM, Wu S, Ruan J and Ma ZS. DBG2OLC: efficient assembly of large genomes using long erroneous reads of the third generation sequencing technologies. Scientific reports. 2016;6:31900.
23. Langmead B and Salzberg SL. Fast gapped-read alignment with Bowtie 2. Nature methods. 2012;9 4:357-9.
24. Li H, Handsaker B, Wysoker A, Fennell T, Ruan J, Homer N, et al. The sequence alignment/map format and SAMtools. Bioinformatics. 2009;25 16:2078-9.
25. Walker BJ, Abeel T, Shea T, Priest M, Abouelliel A, Sakthikumar S, et al. Pilon: an integrated tool for comprehensive microbial variant detection and genome assembly improvement. PloS one. 2014;9 11:e112963.
26. Vinogradov AE. Genome size and GC-percent in vertebrates as determined by flow cytometry: The triangular relationship. Cytometry Part A. 1998;31 2:100-9.
27. Simão FA, Waterhouse RM, Ioannidis P, Kriventseva EV and Zdobnov EM. BUSCO: assessing genome assembly and annotation completeness with single-copy orthologs. Bioinformatics. 2015;31 19:3210-2.
28. Camacho C, Coulouris G, Avagyan V, Ma N, Papadopoulos J, Bealer K, et al. BLAST+: architecture and applications. BMC bioinformatics. 2009;10 1:421.
29. Mistry J, Finn RD, Eddy SR, Bateman A and Punta M. Challenges in homology search: HMMER3 and convergent evolution of coiled-coil regions. Nucleic acids research. 2013;41 12:e121-e.
30. Keller O, Kollmar M, Stanke M and Waack S. A novel hybrid gene prediction method employing protein multiple sequence alignments. Bioinformatics. 2011;27 6:757-63.
31. Rice P, Longden I and Bleasby A. EMBOSS: the European molecular biology open software suite. Elsevier Current Trends, 2000.
32. Bachmann K. Specific nuclear DNA amounts in toads of the genus Bufo. Chromosoma. 1970;29 3:365-74.
33. Camper J, Ruedas L, Bickham J and Dixon J. The relationship of genome size with developmental rates and reproductive strategies in five families of neotropical bufonoid frogs. Life Sci Adv. 1993;12:79-87.
34. Bachmann K. Nuclear DNA and developmental rate in frogs. Quarterly Journal of the Florida Academy of Sciences. 1972;35 4:225-31.
35. Chipman AD, Khaner O, Haas A and Tchernov E. The evolution of genome size: what can be learned from anuran development? Journal of Experimental Zoology Part A: Ecological Genetics and Physiology. 2001;291 4:365-74.
36. Griffin C, Scott D and Papworth D. The influence of DNA content and nuclear volume on the frequency of radiation-induced chromosome aberrations in Bufo species. Chromosoma. 1970;30 2:228-49.
37. Goin OB, Goin CJ and Bachmann K. DNA and amphibian life history. Copeia. 1968:532-40.

38. MacCulloch RD, Upton DE and Murphy RW. Trends in nuclear DNA content among amphibians and reptiles. Comparative Biochemistry and Physiology Part B: Biochemistry and Molecular Biology. 1996;113 3:601-5.
39. Marçais G and Kingsford C. A fast, lock-free approach for efficient parallel counting of occurrences of k-mers. Bioinformatics. 2011;27 6:764-70.
40. Vurture GW, Sedlazeck FJ, Nattestad M, Underwood CJ, Fang H, Gurtowski J, et al. GenomeScope: fast reference-free genome profiling from short reads. Bioinformatics. 2017;33 14:2202-4.
41. Wilhelm J, Pingoud A and Hahn M. Real-time PCR-based method for the estimation of genome sizes. Nucleic Acids Research. 2003;31 10:e56-e.
42. Holt C and Yandell M. MAKER2: an annotation pipeline and genome-database management tool for second-generation genome projects. BMC bioinformatics. 2011;12 1:491.
43. Slater GSC and Birney E. Automated generation of heuristics for biological sequence comparison. BMC bioinformatics. 2005;6 1:31.
44. Smit AFA, Hubley R and Green P. 2013–2015. RepeatMasker Open-4.0. 2013. http://www.repeatmasker.org/.
45. Hubley R, Finn RD, Clements J, Eddy SR, Jones TA, Bao W, et al. The Dfam database of repetitive DNA families. Nucleic acids research. 2015;44 D1:D81-D9.
46. Smit AFA and Hubley R. 2008–2015. RepeatModeler Open-1.0. 2008. http://www.repeatmasker.org/.
47. Korf I. Gene finding in novel genomes. BMC bioinformatics. 2004;5 1:59.
48. Apweiler R, Bairoch A, Wu CH, Barker WC, Boeckmann B, Ferro S, et al. UniProt: the Universal Protein knowledgebase. Nucleic Acids Research. 2004;32 suppl_1:D115-D9. doi:10.1093/nar/gkh131.
49. Richardson MF, Sequeira F, Selechnik D, Carneiro M, Vallinoto M, Reid JG, et al. Supporting data for "Improving amphibian genomic resources: a multitissue reference transcriptome of an iconic invader." Giga-Science Database 2017. http://dx.doi.org/10.5524/100374.
50. Davey NE, Shields DC and Edwards RJ. SLiMDisc: short, linear motif discovery, correcting for common evolutionary descent. Nucleic acids research. 2006;34 12:3546-54.
51. Patro R, Duggal G, Love MI, Irizarry RA and Kingsford C. Salmon provides fast and bias-aware quantification of transcript expression. Nature methods. 2017;14 4:417.
52. Altenhoff AM, Boeckmann B, Capella-Gutierrez S, Dalquen DA, DeLuca T, Forslund K, et al. Standardized benchmarking in the quest for orthologs. Nature methods. 2016;13 5:425.
53. Lee E, Helt GA, Reese JT, Munoz-Torres MC, Childers CP, Buels RM, et al. Web Apollo: a web-based genomic annotation editing platform. Genome biology. 2013;14 8:R93.
54. Davey NE, Edwards RJ and Shields DC. The SLiMDisc server: short, linear motif discovery in proteins. Nucleic acids research. 2007;35 suppl_2:W455-W9.
55. Katoh K and Standley DM. MAFFT multiple sequence alignment software version 7: improvements in performance and usability. Molecular biology and evolution. 2013;30 4:772-80.
56. Nguyen L-T, Schmidt HA, von Haeseler A and Minh BQ. IQ-TREE: a fast and effective stochastic algorithm for estimating maximum-likelihood phylogenies. Molecular biology and evolution. 2014;32 1:268-74.
57. Creevey C and McInerney JO. Clann: investigating phylogenetic information through supertree analyses. Bioinformatics. 2004;21 3:390-2.
58. Rambaut A. FigTree v1.4. Molecular evolution, phylogenetics and epidemiology Edinburgh, UK: University of Edinburgh, Institute of Evolutionary Biology. 2012.

Tables

Table 1. Summary statistics of generated whole genome shotgun sequencing data. Bold rows indicate data used for assembly.

Platform	Library Type	Mean insert size (kb)	Mean read length (bp)	Number of reads	Number of bases (Gb)
HiSeqX (raw)	Paired-end	0.35	147.7	1,857,762,090	282.92
**HiSeqX (filtered)**			**140.6**	**1,205,616,705**	**169.47**
PacBio RS II	SMRTbell	15-50	8,852	2,794,391	24.736
PacBio RS II	SMRTbell	15-50	9,085	595,447	5.409
PacBio RS II	SMRTbell	15-50	10,432	1,867,543	19.482
PacBio RS II	SMRTbell	20-50	10,834	2,487,852	26.952
**PacBio Total**			**9,887**	**7,745,233**	**76.58**
**PacBio Unique[1]**			**10,987**	**6,167,714**	**67.77**

1. Longest read per sequenced molecule (SMRT ZMW).

Table 2. Summary of genome assemblies. For comparison, statistics are provided for two existing neobatrachian genomes, *Nanorana parkeri* (v2) [15] and *Lithobates catesbeianus* (v2.1)[14], and two anuran reference genomes, *Xenopus tropicalis* (v9.1) [16] and *Xenopus laevis* (v9.2) [17]. Lengths are given to 3 s.f. All percentages are given to 1 d.p.

**Genome Assembly**	**Hybrid (v2.2)**	**Short read**	**Long read**	***N. parkeri* (v2.0)**	***L. catesbeianus* (v2.1)**	***X. tropicalis* (v9.1)**	***X. laevis* (v9.2)**
Total Length (Gb)	2.55	3.75	2.69	2.07	6.25	1.44	2.72
No. scaffolds	31,392	19.9 M*	31,392*	135,808	1.54 M	6,822	108,033
Proportion gap (%N)	0.0%	0.1%	0.0%	3.9%	11.6%	4.9%	11.4%
N50	168 kb	583 bp	167 kb	1.06 Mb	39.4 kb	135 Mb	137 Mb
L50	3,373	715 k	3,531	555	31,248	5	9
Longest scaffold	3.53 Mb	72.6 kb	3.64 Mb	8.61 Mb	1.38 Mb	195 Mb	220 Mb
GC	43.2%	43.3%	42.9%	42.6%	43.1%	40.1%	39.0%
**BUSCO[1]**							
Complete Single copy	80.9%	15.5%	2.2%	83.4%	42.3%	87.5%	52.9%
Complete Duplicate	2.2%	0.7%	0.0%	1.6%	0.9%	1.0%	39.8%
Fragment	7.5%	33.6%	2.2%	7.2%	22.3%	6.0%	3.2%

1. BUSCO v2.0.1 short summary statistics (n=3950).

* Statistics for short and long read assemblies refer to contigs used for hybrid assembly.

Table 3. GenomeScope genome size estimates for *Rhinella marina* based on raw trimmed Illumina data using different combinations of k and maximum k-mer coverage. Lengths are in megabases (0 d.p.).

**Data**	**Max kmer coverage**	**Unique Length (Mb)**		**Repeat Length (Mb)**		**Genome Size (Mb)**	
		**Min**	**Max**	**Min**	**Max**	**Min**	**Max**
Raw (k=21)	1000	1,365	1,366	489	489	1,853	1,855
Raw (k=21)	10000	1,365	1,365	874	874	2,239	2,240
Raw (k=23)	1000	1,453	1,455	470	471	1,924	1,926
Raw (k=23)	10000	1,454	1,454	842	842	2,296	2,296
Q30 (k=21)	1000	1,307	1,308	462	462	1,768	1,771
Q30 (k=21)	10000	1,307	1,308	749	749	2,056	2,057
Q30 (k=23)	1000	1,389	1,391	438	439	1,828	1,830
Q30 (k=23)	10000	1,390	1,391	713	713	2,103	2,104

Table 4. Estimation of *Rhinella marina* genome size using various methods and the corresponding level of sequencing coverage (3 s.f.). GenomeScope values in this table are mean values from the four setting combinations.

**Method**	**Estimated Genome Size (Gb)**	**Illumina coverage (X)**	**PacBio coverage (X)**	**Reference**
Flow cytometry (mean)	4.33	65.3	17.7	[26, 33, 35, 38]
Flow cytometry (min)	3.98	71.1	19.2	[38]
Flow cytometry (max)	4.90	57.7	15.6	[35]
Densitometry (mean)	4.95	57.1	15.5	[32, 34, 36, 37]
Densitometry (min)	4.06[#]	69.7	18.9	[37]
Densitometry (max)	5.65	50.1	13.6	[32]
GenomeScope (raw)	2.08	136	36.8	-
GenomeScope (Q30)	1.94	146	39.4	-
qPCR (zfp292)	2.38	119	32.1	-
Assembly (v2.2)	2.55	111	30.0	-

value adjusted to account for updated size of reference genome used to infer *R. marina* genome size.

Table 5. Summary statistics of consensus protein-coding gene predictions and predicted repeat elements (including RNA genes) for the *Rhinella marina* v2.2 draft genome. Lengths are given to 3 s.f. Coverage and mean depth statistics for PacBio and Q30-trimmed Illumina reads are given to 2 d.p.

Element	Count	No. scaffolds	Avg. length	Total length	Genome coverage	PacBio depth (X)	Illumina depth (X)
Protein-coding gene	58,302	19,530	18.8 kb	1.10 Gb	42.91%	20.32	58.07
Transcript	58,302	19,530	1.24 kb	72.3 Mb	2.83%	20.49	65.41
- Similar to known	25,846	11,918	1.90 kb	49.1 Mb	1.92%	20.08	56.42
- Unknown	32,456	15,213	714 bp	23.2 Mb	0.91%	20.98	68.82
Exon	309,718	19,530	233 bp	72.3 Mb	2.83%	20.49	65.41
- Coding	294,535	19,530	207 bp	60.8 Mb	2.38%	20.67	66.97
Intron	251,416	18,509	4.08 kb	1.03 Gb	40.09%	20.30	57.55
5’ UTR	15,855	8,839	208 bp	3.29 Mb	0.13%	18.69	53.86
CDS	58,302	19,530	1.04 kb	60.8 Mb	2.38%	20.67	66.97
3’ UTR	11,965	5,780	682 bp	8.16 Mb	0.32%	19.91	58.52
BUSCO SC Complete	3,194	2,014	32.6 kb	104 Mb	4.07%	19.89	53.01
**Repeats**							
SINE	21,620	9,322	338 bp	7.31 Mb	0.29%	19.45	58.23
LINE	268,569	27,620	513 bp	138 Mb	5.38%	21.03	72.29
LTR	201,817	24,949	504 bp	102 Mb	3.98%	22.62	68.96
DNA	817,405	30,689	600 bp	490 Mb	19.17%	21.67	68.37
Helitron	20,319	9,340	826 bp	16.8 Mb	0.66%	19.32	56.81
Retroposon	1,042	829	549 bp	570 kb	0.02%	18.22	50.87
Other	18	17	209 bp	3.7 kb	0.00%	14.27	24.60
Unknown	1,610,883	30,966	513 bp	826 Mb	32.28%	20.12	59.39
Satellite	25,557	10,270	440 bp	11.3 Mb	0.44%	18.38	54.21
Simple repeats	968,947	30,620	56.9 bp	55.1 Mb	2.16%	18.88	48.51
Low complexity	141,028	24,020	51.8 bp	7.30 Mb	0.29%	22.48	64.48
rRNA	5,227	2,923	422 bp	2.20 Mb	0.09%	40.88	142.42
tRNA	5,558	4,474	105 bp	583 kb	0.02%	29.15	140.06
snRNA	21,788	9,432	546 bp	11.9 Mb	0.47%	24.63	89.12

srpRNA	17	11	268 bp	4.55 kb	0.00%	22.11	140.44
scRNA	3	3	69.0 bp	207 bp	0.00%	15.53	47.29
RNA	418	266	482 bp	202 kb	0.01%	32.65	173.99
**Repeat TOTAL**[1]	4,110,222	31,179	406 bp	1.63 Gb	63.9%	20.82	63.79

1. Values for repeat totals account for overlapping repeats.

Table 6. Proportions of predicted protein and transcript sequences exceeding 50%, 80%, 95% or 99% coverage in the top BLAST+ hit from the published transcriptome [18], and combined coverage for the top ten transcript hits. All percentages given to 3 s.f.

Type	Count	Coverage in top transcript hit				Coverage in top 10 transcript hits			
		50%+	80%+	95%+	99%+	50%+	80%+	95%+	99%+
Protein (similar to known)	25,846	93.6	76.7	56.5	40.7	97.5	90.3	72.7	54.2
Transcript (similar to known)	25,846	75.0	50.0	30.8	21.4	82.6	73.1	57.2	40.9
Protein (unknown)	32,456	79.9	49.8	27.1	15.8	85.7	66.3	44.4	29.9
Transcript (unknown)	32,456	43.6	21.5	12.1	8.61	52.6	37.3	25.4	19.1

Figure legends

Figure 1. *Rhinella marina.* An adult cane toad.

Figure 2. Schematic overview of project workflow. A summary of the experimental methods used for sequencing, assembly, annotation and size estimation of the cane toad genome. Transcriptome data (orange segment) was obtained from our previous study [18].

Figure 3. Assessment of genome assembly completeness. BUSCO analysis of *Rhinella marina* genome assembly (v2.0 uncorrected, v2.1 pilon polishing, v2.2 pilon and arrow polishing, combined v2.1, 2.2 and 2.2 ratings), *Lithobates catesbeianus* (v2.1), *Nanorana parkeri* (v2.0), *Xenopus tropicalis* (v9.1) and *Xenopus leavis* (v9.2) genomes using the tetrapoda_odb9 orthologue set (n=3950). The *Xenopus leavis* genome duplication is made clear by the large number of paralogs (light blue) with respect to other assemblies.

Figure 4. GenomeScope k-mer frequency and log-transformed k-mer coverage profiles. (A) raw Illumina data (k=23), (B) Q30 trimmed Illumina data (k=23). Profiles for k=21 are similar (data not shown).

Figure 5. Key protein statistics for predicted genes with and without annotated similarity to known genes. Histograms of (A) protein length, and (B) MAKER2 Annotation Edit Distance (AED), for "similar" (blue) and "unknown" (red) classes of predicted genes.

Figure 6. Multi-tissue gene expression for predicted genes with and without annotated similarity to known genes. (A) Histograms of RNA-Seq TPM for "similar" (blue) and "unknown" (red) classes of predicted genes, capped at 100 TPM. (B) "similar" and (C) "unknown" gene expression, rated as: Very low (<1 TPM), Low (1-9 TPM), Medium (10-99 TPM) or High (100+ TPM).

Figure 7. Assessment of assembly annotation completeness. BUSCO analysis for (A) all BUSCO tetrapoda genes (n=3950), and (B) the subset of BUSCO genes rated as "Missing" from the Richardson *et al.* transcriptome [18]. *R. marina* (combined): combined v2.0, v2.1 and v2.2 ratings; Annotation:

combined MAKER proteome and transcriptome ratings; GigaDB: combined assembly and annotation ratings; Cane Toad: combined assembly, annotation and Richardson *et al.* transcriptome [18].

Figure 8. Phylogenetic supertree of 15 selected chordate taxa constructed from phylogenetic trees for 6,417 high confidence cane toad proteins. Branch labels indicate percentage consistency (see text), rounded down. Numbers following each taxon are the number and percentage of source trees containing that taxon. The tree has been rooted using fish as an outgroup and visualised with FigTree [58]. The full supertree of 52 taxa is available as Figure S1.

Figure 9. Summary of the main annotation classes for *Rhinella marina* genome assembly. Identified repeat classes exceeding 2% of assembly have been plotted separately (1 d.p.). All other repeats, including "Unknown", have been grouped as "Other repeats". The percentage for introns excludes any repeat sequences within those introns.

Figure 1

Click here to access/download;Figure;Figure 1.eps

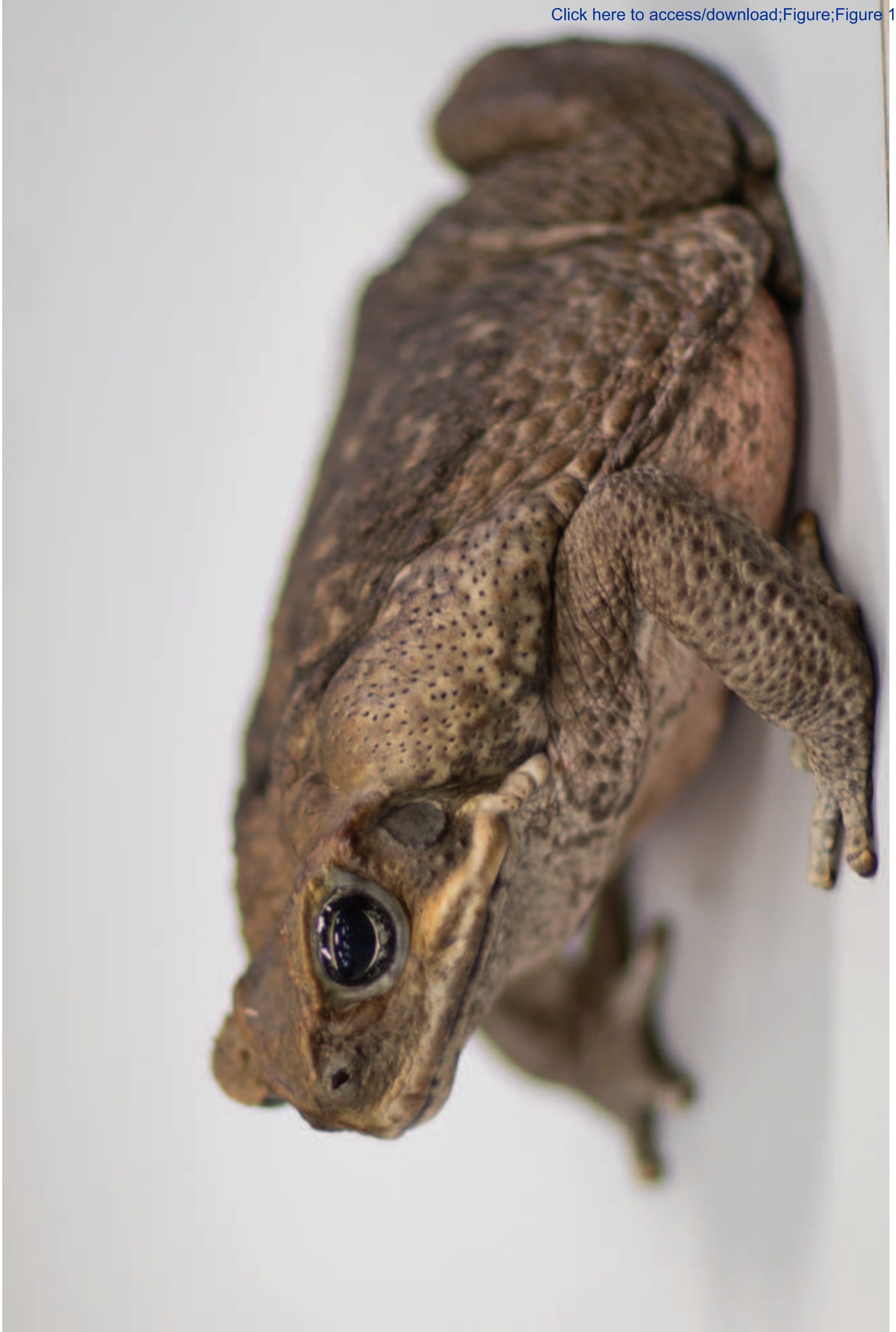

Figure 2

Click here to access/download;Figure;Figure 2.eps

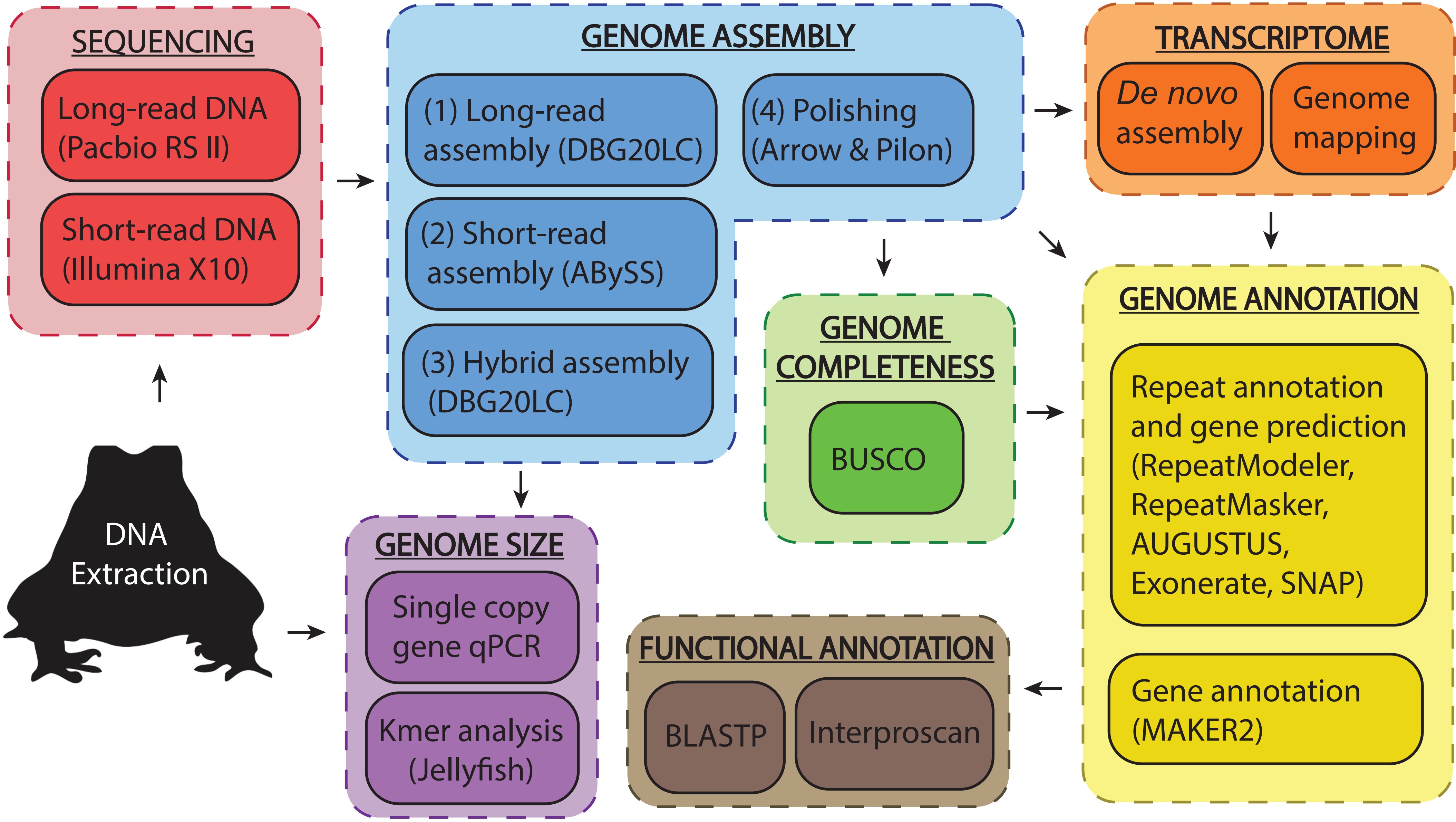

Figure 3

Click here to access/download;Figure;Figure 3.eps

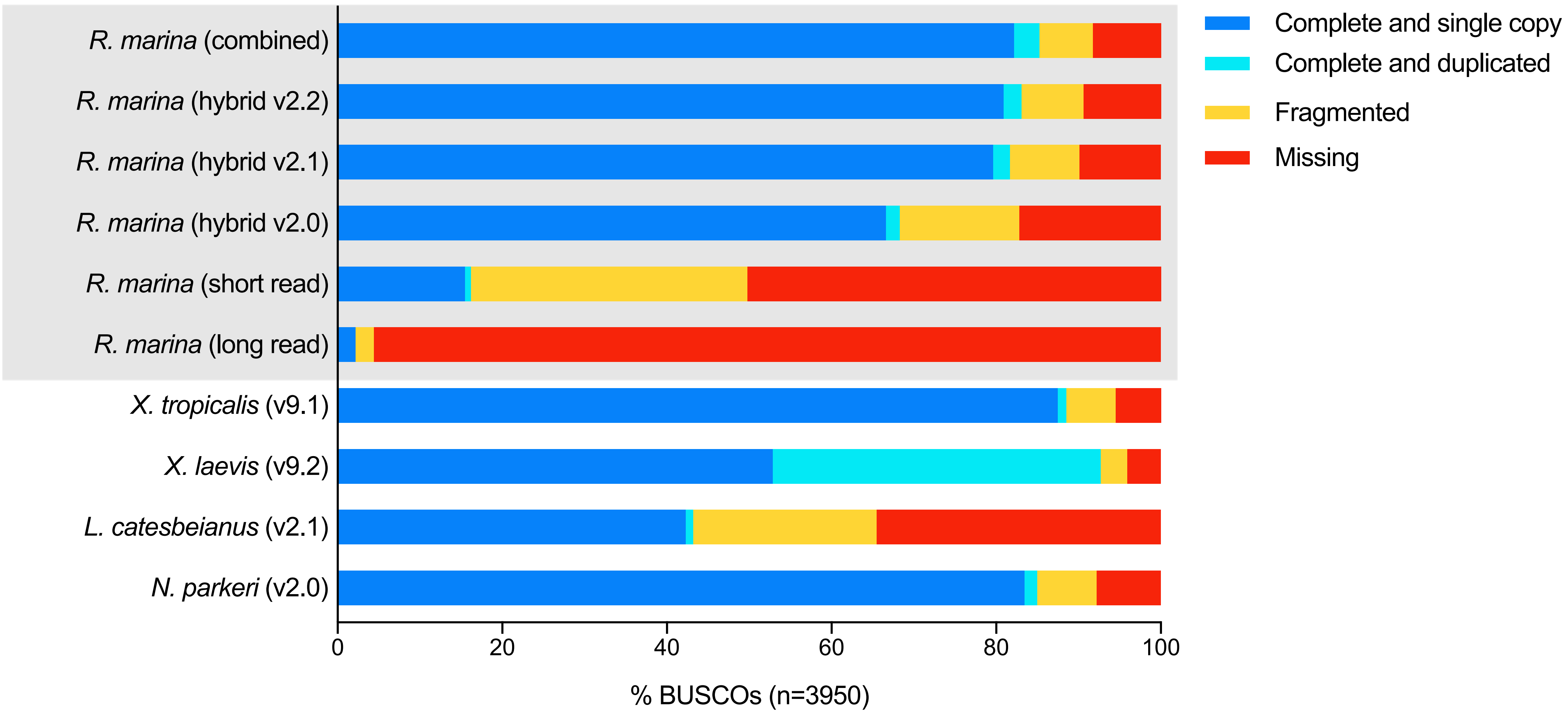

Click here to access/download;Figure;Figure 4.eps

Figure 4

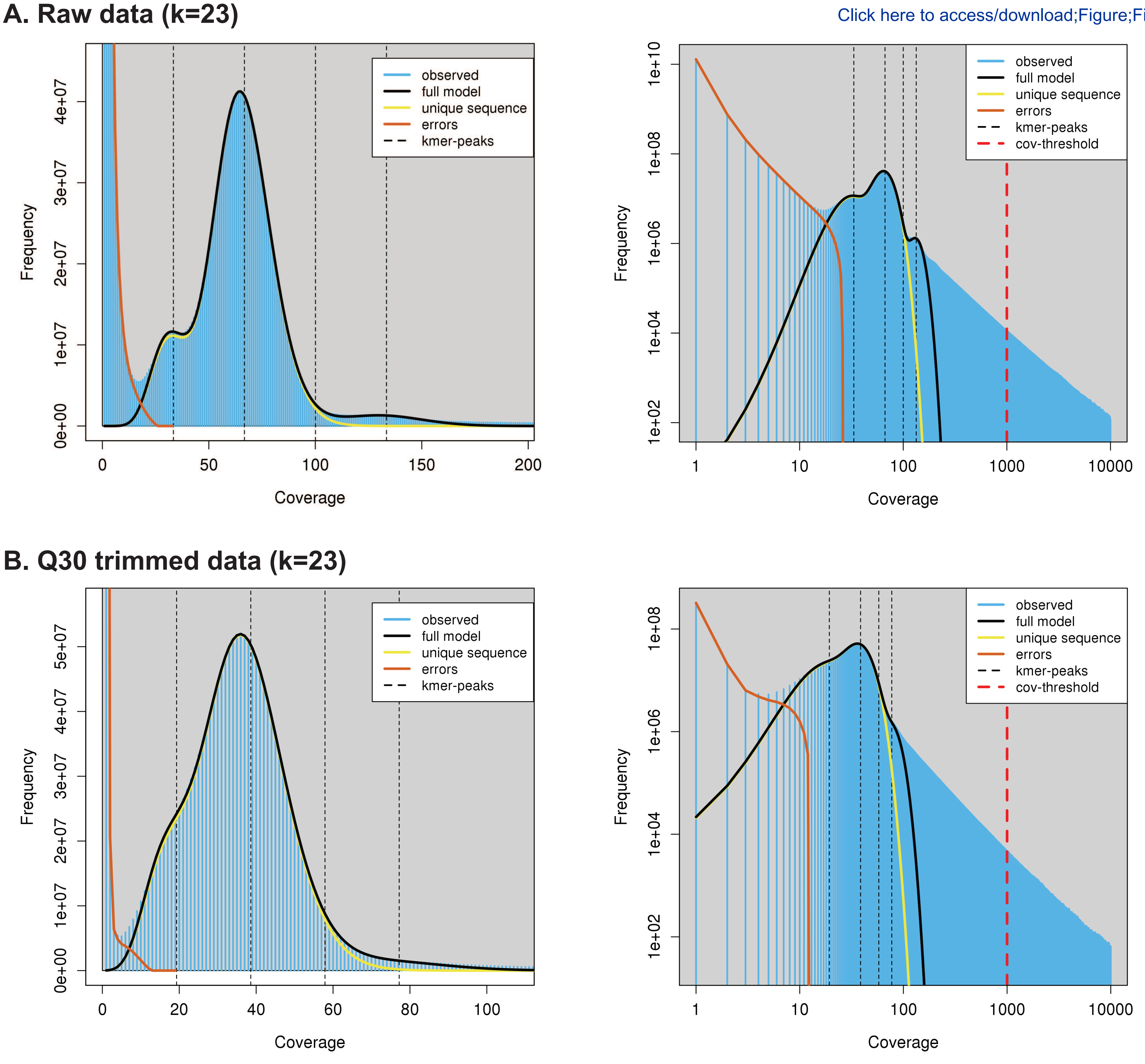

Figure 5

Click here to access/download;Figure;Figure 5.pdf

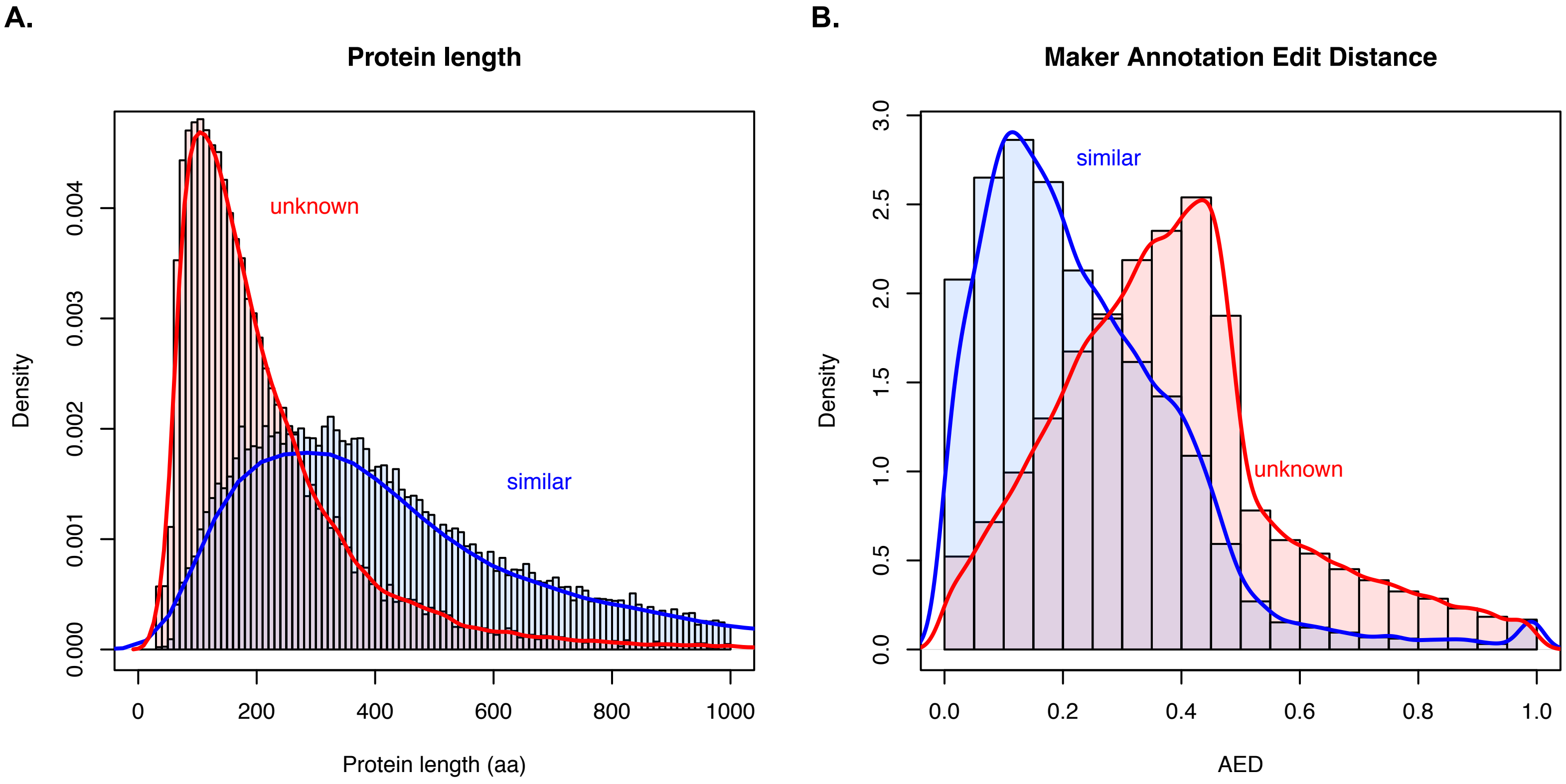

Figure 6

Click here to access/download;Figure;Figure 6.pdf

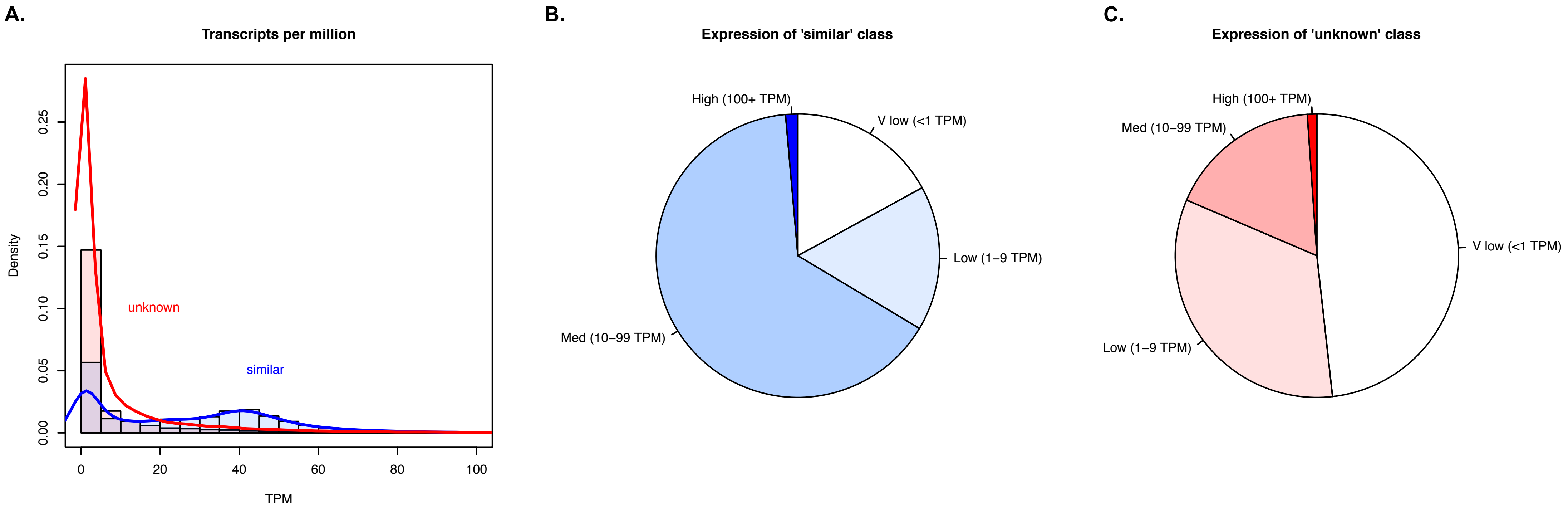

Figure 7

Click here to access/download;Figure;Figure 7.eps

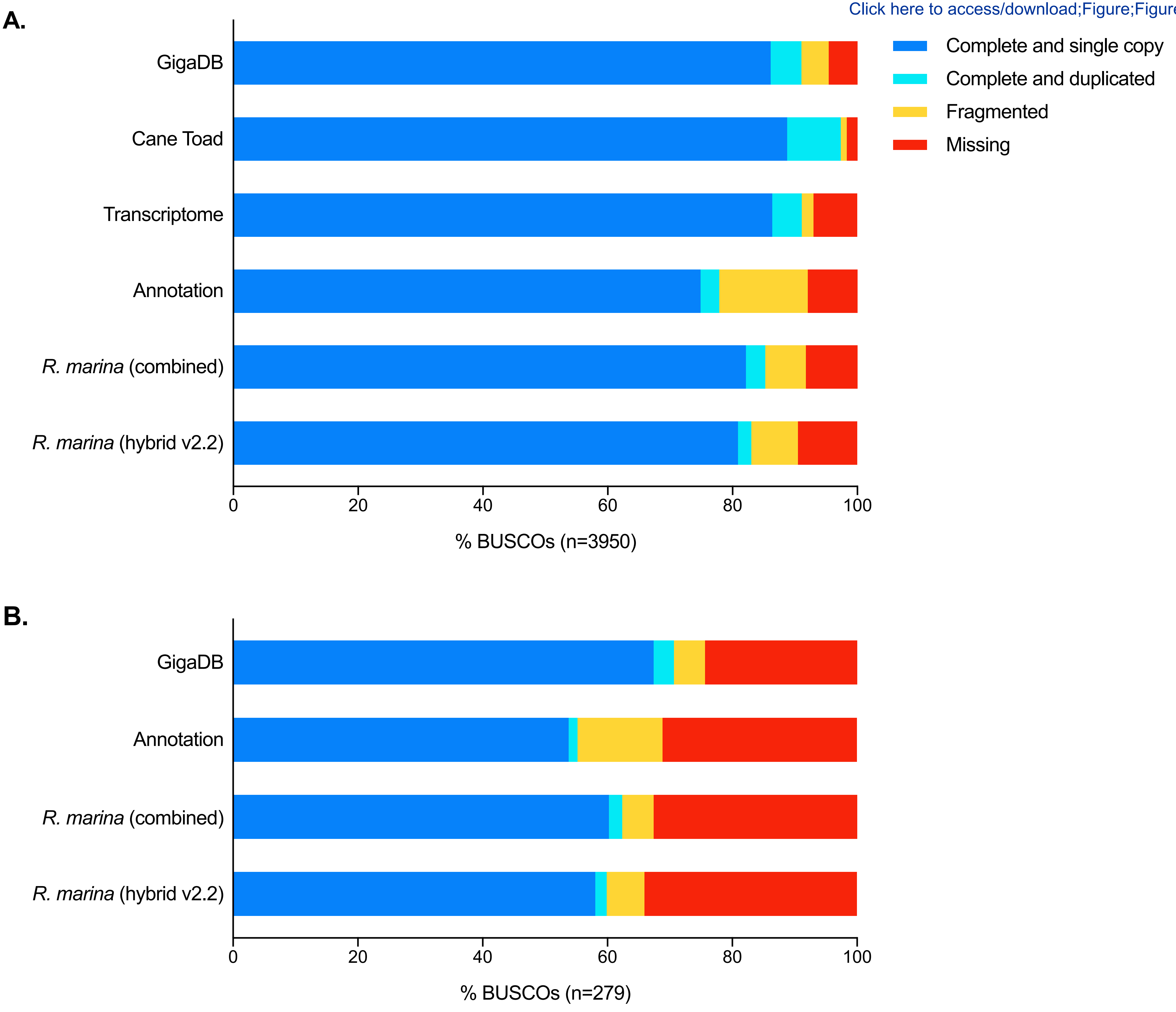

Figure 8

Click here to access/download;Figure;Figure 8.pdf

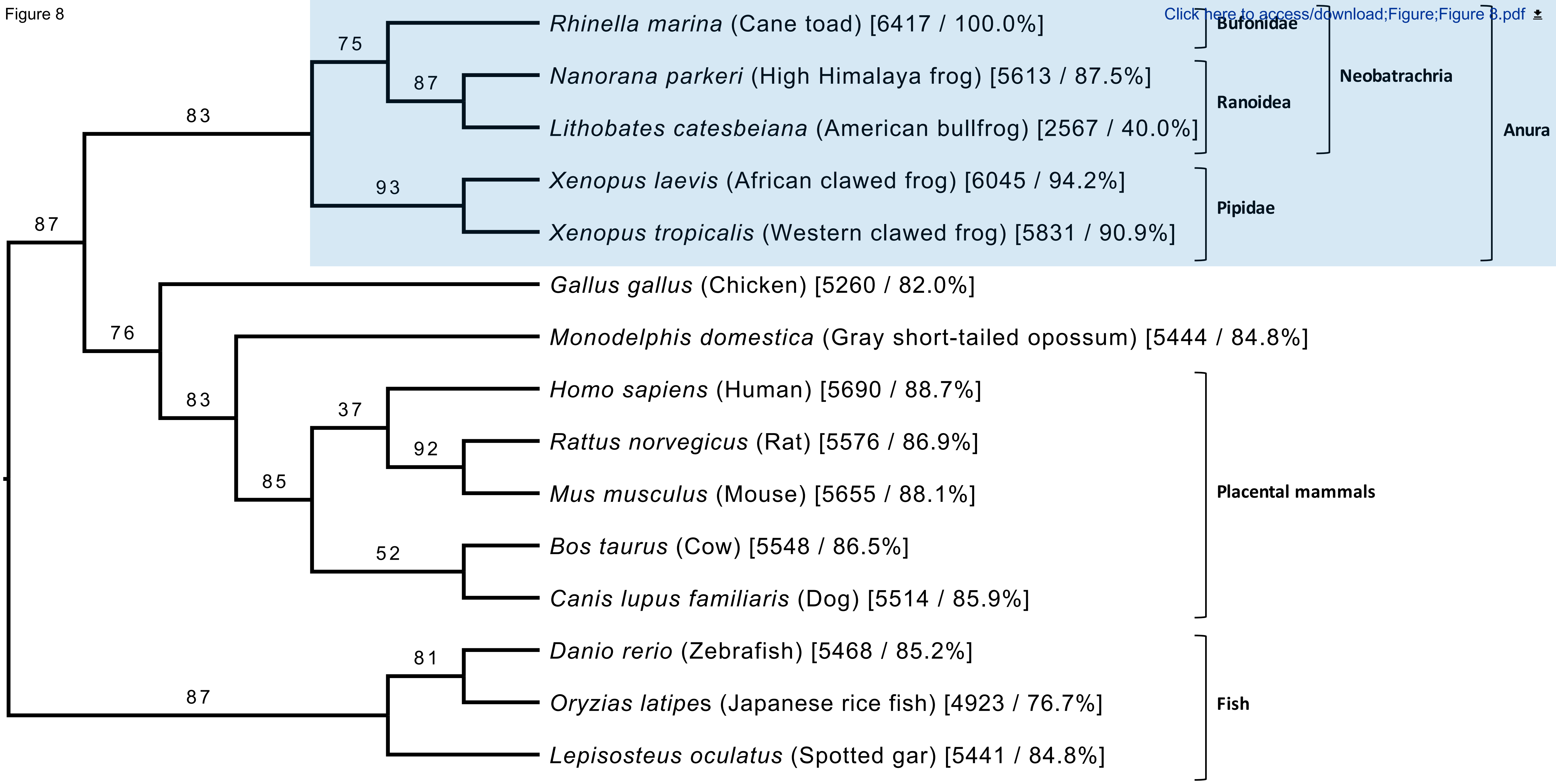

Figure 9

Click here to access/download;Figure;Figure 9.eps

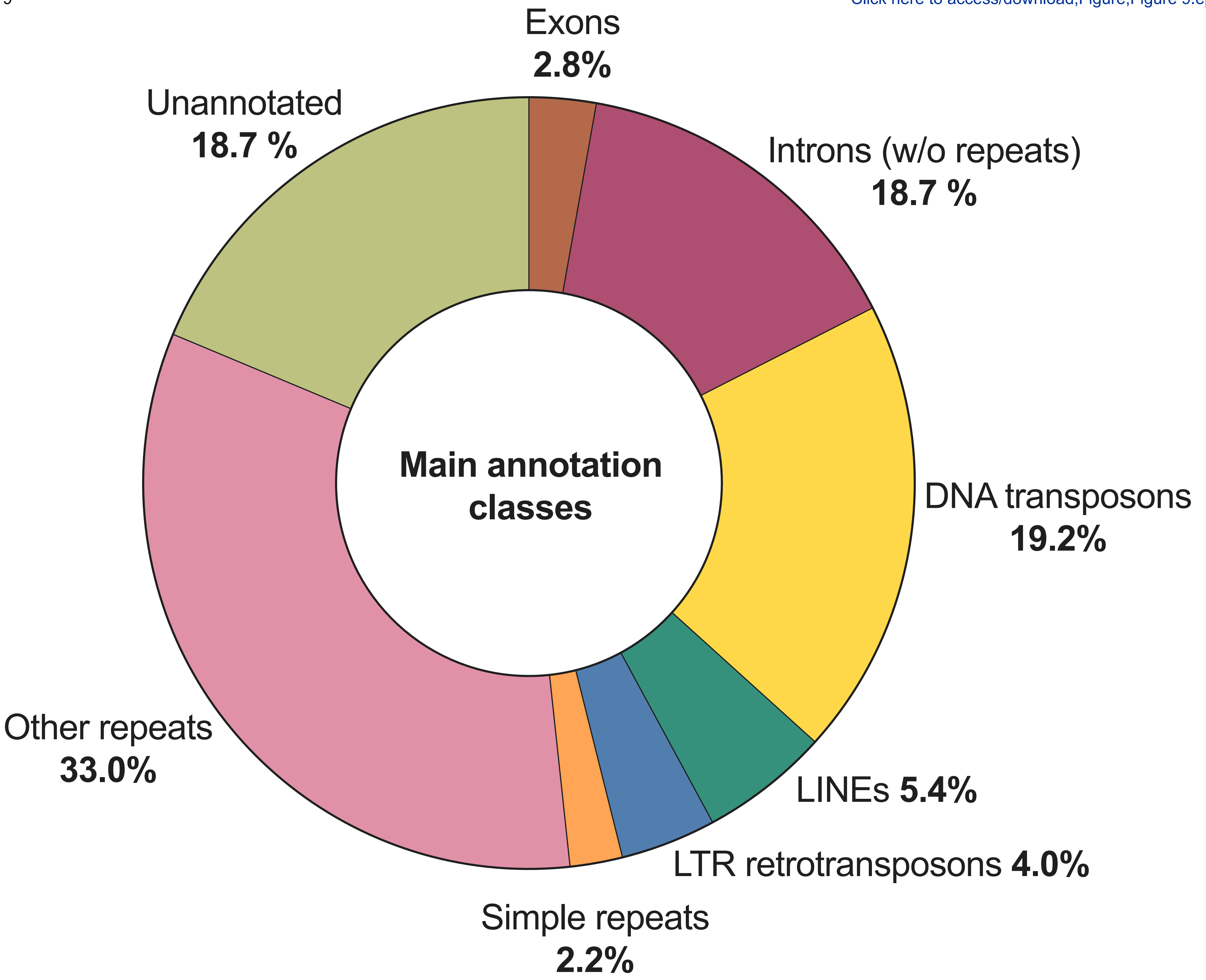

Figure S1

Click here to access/download
Supplementary Material
Figure S1.pdf

Table S1

Click here to access/download
Supplementary Material
Table S1.xlsx

Table S2

Click here to access/download
Supplementary Material
Table S2.xlsx

Table S3

Click here to access/download
Supplementary Material
Table S3.xlsx

Table S4

Click here to access/download
Supplementary Material
Table S4.xlsx
