# Supplementary material for: Draft genome assembly of the invasive cane toad, Rhinella marina
Source: Gigascience. 2018 Aug 7;7(9):giy095. doi: 10.1093/gigascience/giy095 (PMC6145236; doi:10.1093/gigascience/giy095)
Supplement: GIGA-D-18-00104_Revision_3.pdf [file giy095_giga-d-18-00104_revision_3.pdf]

GigaScience

Draft genome assembly of the invasive cane toad, Rhinella marina

--Manuscript Draft--

**Manuscript Number:**	GIGA-D-18-00104R3
**Full Title:**	Draft genome assembly of the invasive cane toad, Rhinella marina
**Article Type:**	Data Note
**Funding Information:**	Australian Research Council (DP160102991) — Dr. Lee A Rollins; Prof. Richard Shine<br>Australian Research Council (DE150101393) — Dr. Lee A Rollins<br>Australian Research Council (FL170100022) — Prof. Edward C Holmes<br>Australian Research Council (LE150100031) — Prof. Marc R Wilkins<br>Australian Research Council (FL120100074) — Prof. Richard Shine<br>The National Council for Scientific and Technological Development (CNPq) (302892/2016-8) — Assoc. Prof. Marcelo Vallinoto<br>Bioplatforms Australia — Not applicable
**Abstract:**	Background: The cane toad (Rhinella marina formerly Bufo marinus) is a species native to Central and South America that has spread across many regions of the globe. Cane toads are known for their rapid adaptation and deleterious impacts on native fauna in invaded regions. However, despite an iconic status, there are major gaps in our understanding of cane toad genetics. The availability of a genome would help to close these gaps and accelerate cane toad research. Findings: We report a draft genome assembly for R. marina, the first of its kind for the Bufonidae family. We used a combination of long read PacBio RS II and short read Illumina HiSeq X sequencing to generate a total of 359.5 Gb of raw sequence data. The final hybrid assembly of 31,392 scaffolds was 2.55 Gb in length with a scaffold N50 of 168 kb. BUSCO analysis revealed that the assembly included full length or partial fragments of 90.6% of tetrapod universal single-copy orthologs (n=3950), illustrating that the gene-containing regions have been well-assembled. Annotation predicted 25,846 protein coding genes with similarity to known proteins in SwissProt. Repeat sequences were estimated to account for 63.9% of the assembly. Conclusion: The R. marina draft genome assembly will be an invaluable resource that can be used to further probe the biology of this invasive species. Future analysis of the genome will provide insights into cane toad evolution and enrich our understanding of their interplay with the ecosystem at large.
**Corresponding Author:**	Peter White, Ph.D.<br>University of New South Wales<br>Sydney, NSW AUSTRALIA
**Corresponding Author Secondary Information:**	
**Corresponding Author's Institution:**	University of New South Wales
**Corresponding Author's Secondary Institution:**	
**First Author:**	Richard J Edwards
**First Author Secondary Information:**	
**Order of Authors:**	Richard J Edwards<br>Daniel Enosi Tuipulotu<br>Timothy G Amos

Powered by Editorial Manager® and ProduXion Manager® from Aries Systems Corporation

	Denis O'Meally
	Mark F Richardson
	Tonia L Russell
	Marcelo Vallinoto
	Miguel Carneiro
	Nuno Ferrand
	Marc R Wilkins
	Fernando Sequeira
	Lee A Rollins
	Edward C Holmes
	Richard Shine
	Peter A White
**Order of Authors Secondary Information:**	
**Response to Reviewers:**	Please note that quoted text is from the manuscript. Additions or modifications to text within the manuscript are explicitly stated.<br><br>Comment 1.<br><br>This manuscript adopted "a hybrid de novo whole genome assembly strategy," a relatively new technique, which should require more quality controls than the conventional technique combining shotgun sequences, mate-pair sequences, PacBio long read sequences, and Hi-C or CHICAGO methods. Most comments from the reviewers including mine for basic analyses with the assembly are for quality controls, not for analyses "beyond the scope of this paper," as the authors say. Furthermore, they used a wild-caught frog, which must contain distinct alleles, probably making assembly processes difficult. How did the authors overcome allelic differences? I'm worried about one undesired possibility that the current assembly contains one of the two alleles, and short scaffolds contain fragments of the other alleles, which may explain many fragmented ORFs in the assembly as well as underestimation of the genome size by the k-mer genome size estimation and qPCR. Do the authors have any evidence to exclude this possibility?<br><br>Response to comment 1:<br><br>The DBG2OLC technique we employed is not unconventional. It has been used in over a dozen genome assemblies, including those published in GigaScience (golden mussel, European beech & Chinese herbal fleabane), Nature Genetics (apple & sea lamprey), Nature Plants (Xerophyta viscosa), Cell (Egyptian Rousette bat), and Genome Biology and Evolution (clam shrimp). Genome assembly is not a 'solved problem' and few (if any) assemblies use identical combinations of technology, sequencing depth and assembly/scaffolding methods. We agree that independent assembly of allelic variants is a potential issue for heterozygous non-haploid assemblies, and highlight it as a possible cause of the high ORF numbers (L225-7):<br><br>“artefactual duplications in the genome assembly, either through under-assembly or legitimate assembly of two heterozygous diploid copies;”<br><br>And L231-2:<br><br>“Of the 3,279 complete BUSCO genes identified (Table 2), only 85 (2.59%) were duplicated. This suggests that there is not widespread duplication in the assembly.”<br><br>In addition, extensive assembly of allelic variants would inflate the genome assembly size dramatically, and we see no evidence of this. We also see a trend in the data (consistent with the contiguity statistics) that fragmentation is a likely cause for inflated

	ORF counts (L264-9):<br><br>“Excluding proteins with annotation indicating possible viral or transposable element origin, 45.7% of "similar" proteins and 96.8% of “unknown” proteins had the same closest X. tropicalis blastp hit as another predicted protein. Consistent with this being related to gene fragmentation, there was a negative relationship between the number of cane toad proteins sharing a given X. tropicalis top hit, and how much of the X. tropicalis hit was covered by each cane toad protein.”<br><br>Although the evidence presented makes widespread duplicated assembly (allelic or otherwise) unlikely, we acknowledge that, as with all draft assemblies, there will be some scaffolds and ORFs that represent allelic variants. We have therefore added this caveat (L269-70):<br><br>“Nevertheless, it is likely that some of these protein fragments represent allelic variants that have been redundantly assembled.” [additional text in manuscript]<br><br>There is no consistent way to globally identify and distinguish these from duplications, particularly in a repeat-rich genome like the cane toad. We have therefore opted to adopt a conservative filtering approach as detailed analysis of genes/regions of interest should identify any such issues on a case-by-case basis. As previously noted (and see point 4 below), we have unambiguously stated that our statistics refer to the assembly and we stop short of making unsubstantiated claims about the cane toad genome. Impact on genome size is discussed in Point 2, below.<br><br><br>Comment 2:<br><br>In addition, the reported genome sizes of Rhinella marina (the same as Bufo marinus) varied between 3.98 and 5.65 Gb [26, 32-38]. Among the cited references, the papers by MacCulloch et al. (1996) and Chipman et al. (2001) appear to be reliable, because, in comparison with the genome size of Xenopus laevis (3.1 Gb), that of Bufo marinus was estimated to be 3.98 and 3.59, respectively, (the mean is 3.77 Gb) by assuming that 1pg DNA corresponds to 1 Gb. By the way, is Rhinella marina truly diploid? If so, its genome contains much more transposable elements and/or repetitive sequences than the allotetraploid genome of Xenopus laevis. According to the X. laevis genome paper (Session et al., 2016), total shotgun sequences in contigs (nucleotide stretches without N) are 2.45 Gb in allotetraploid X. laevis, which is similar to the final hybrid assembly of 2.55 Gb in diploid R. marina. This might imply again artificial sequence redundancy in the hybrid assembly due to allelic differences in wild R. marina. This may also explain the inconsistency between the flow cytometry-based genome size of 3.77 Gb and the k-mer-estimated genome size of ~2.0 Gb. Did the authors check artificial internal redundancy due to the two distinct alleles? The authors need to discuss this kind of issue in their paper.<br><br><br>Response to comment 2:<br><br>We have no evidence against diploidy in Rhinella marina and the published karyotype does not show evidence of higher ploidy. We did consider artificial sequence redundancy, however this would inflate the estimated genome size (and assembly), not reduce it, and so it cannot be the explanation for the observed differences. Likewise, if the qPCR primers were allele-specific (point 1), the apparent genome size would be doubled, not halved. The kmer method we used (GenomeScope) was a diploid method and explicitly incorporates allelic variation into its estimation model. As readers may not be familiar with this method, we have expanded our discussion of this issue with an extra a sentence to emphasise this point (L176-7):<br><br>“GenomeScope explicitly models heterozygous diploid kmer distributions, which should make it robust to the additional challenge of sequencing a wild animal. However, GenomeScope predictions are affected by non-uniform repeat distributions and this difference could indicate high copy number repeats in the genome that are difficult to model accurately.” [additional text in manuscript]

Powered by Editorial Manager® and ProduXion Manager® from Aries Systems Corporation

	Comment 3:<br><br>In Summary: According to the authors, "Annotation predicted 58,302 protein coding genes" include many fragmented ORFs. Because of this, the number (58,302) is meaningless, which should be removed from the summary. In the answer, the authors wrote "however many of these may be bona fide functional members of the cane toad proteome," but what is the rational to think like this? For example, what percentage of these ORFs are expressed? In general, such unexpressed ORFs are not counted as protein-coding genes. Therefore, the statement "however many of these may be bona fide functional members of the cane toad proteome" should be deleted if there is no supporting evidence.<br><br>Response to comment 3:<br><br>We have rephrased the sentence in the abstract (L62):<br><br>“Annotation predicted 25,846 protein coding genes with similarity to known proteins in SwissProt.” [modified text in manuscript]<br><br>The manuscript includes analysis and discussion of transcriptomic support for the predictions, including a warning that some of the 58,302 predicted protein coding genes may be false annotations (L242-254). The quoted statement refers to predicted proteins that may originate from transposable elements or viruses. Exaptation of transposons and endogenous viral elements is common in nature and we have no reason to believe that it will not have happened in the cane toad. We have expanded the expression analysis as suggested to support this statement (and moved it to follow discussion of the expression data), L251-4:<br><br>“Further review of the predicted protein descriptions revealed 4,357 with likely origins in transposable elements (including 4,114 LINE-1 ORFs) and 215 from viruses. However, many of these may be bona fide functional members of the cane toad proteome; 1,447 (33.2%) “transposon” and 151 (70.2%) of “viral” transcripts had support for expression > 1 TPM.” [additional text in manuscript]<br><br>Comment 4:<br><br>Fig. 5 (now Fig. 9) represents the feature of the assembly sequence, not the genome. The authors need to carefully state which it is in the figures, legends, and main text.<br><br>Response to comment 4:<br><br>This is clearly stated in the revised text and figure legend (emphasis added):<br><br>“RepeatMasker annotations from the MAKER pipeline support this interpretation, with over 4.1 million repeat sequences detected, accounting for 63.9% of the assembly (Table 5). The mean repeat length is 406 bp, which exceeds the Illumina read length used in our study (mean 140.6 bp paired-end). This makes short-read assembly of these regions difficult, as reflected by the poor ABySS contiguity (contig N50 = 583 bp, Table 2), and emphasises the need for long read data in this organism. The most abundant class of repeat elements are of unknown type (1.61 million elements covering 32.28% of the assembly), with DNA transposons the most abundant known class of element (817,262 repeats; 19.17% coverage). Of these, the most abundant are of the hAT-Ac (231,332 copies) and TcMar-Tc1 (226,145 copies) superfamilies (Table S4). Accounting for overlaps between repeat and gene features, 18.7% of the assembly (479,397,014 bp) has no annotation (Figure 9).”<br><br>The title of the figure is “Summary of the main annotation classes for Rhinella marina genome assembly.”
**Additional Information:**	

Powered by Editorial Manager® and ProduXion Manager® from Aries Systems Corporation

Question	Response
Are you submitting this manuscript to a special series or article collection?	No
**Experimental design and statistics**<br><br>Full details of the experimental design and statistical methods used should be given in the Methods section, as detailed in our Minimum Standards Reporting Checklist. Information essential to interpreting the data presented should be made available in the figure legends.<br><br>Have you included all the information requested in your manuscript?	Yes
**Resources**<br><br>A description of all resources used, including antibodies, cell lines, animals and software tools, with enough information to allow them to be uniquely identified, should be included in the Methods section. Authors are strongly encouraged to cite Research Resource Identifiers (RRIDs) for antibodies, model organisms and tools, where possible.<br><br>Have you included the information requested as detailed in our Minimum Standards Reporting Checklist?	Yes
**Availability of data and materials**<br><br>All datasets and code on which the conclusions of the paper rely must be either included in your submission or deposited in publicly available repositories (where available and ethically appropriate), referencing such data using a unique identifier in the references and in the "Availability of Data and Materials" section of your manuscript.	Yes

Have you have met the above requirement as detailed in our Minimum Standards Reporting Checklist?	

Powered by Editorial Manager® and ProduXion Manager® from Aries Systems Corporation

Manuscript with DOI
Click here to view linked References
Click here to access/download;Manuscript;CaneToadGenomePaper (final)

Draft genome assembly of the invasive cane toad, *Rhinella marina*

Richard J Edwards[1], Daniel Enosi Tuipulotu[1†], Timothy G Amos[1†], Denis O'Meally[2], Mark F Richardson[3,4], Tonia L Russell[5], Marcelo Vallinoto[6,7], Miguel Carneiro[6], Nuno Ferrand[6,8,9], Marc R Wilkins[1,5], Fernando Sequeira[6], Lee A Rollins[3,10], Edward C Holmes[11], Richard Shine[12] and Peter A White[1,*]

[†]These authors contributed equally to the work.

[1]School of Biotechnology and Biomolecular Sciences, University of New South Wales, Sydney, NSW, Australia

[2]Sydney School of Veterinary Science, Faculty of Science, University of Sydney, Camperdown, New South Wales, Australia

[3]School of Life and Environmental Sciences, Centre for Integrative Ecology, Deakin University, Geelong, VIC, Australia

[4]Bioinformatics Core Research Group, Deakin University, Geelong, VIC, Australia

[5]Ramaciotti Centre for Genomics, University of New South Wales, Sydney, NSW, Australia

[6]CIBIO/*InBIO*, Centro de Investigação em Biodiversidade e Recursos Genéticos, Universidade do Porto, Vairão, Portugal

[7]Laboratório de Evolução, Instituto de Estudos Costeiros (IECOS), Universidade Federal do Pará, Bragança, Pará, Brazil

[8]Departamento de Biologia, Faculdade de Ciências, Universidade do Porto, Porto, Portugal

[9]Department of Zoology, Faculty of Sciences, University of Johannesburg, Auckland Park, South Africa

[10]Evolution and Ecology Research Centre, School of Biological Earth and Environmental Sciences, University of New South Wales, Sydney, NSW, Australia

[11]Marie Bashir Institute for Infectious Diseases and Biosecurity, Charles Perkins Centre, School of Life and Environmental Sciences and Sydney Medical School, University of Sydney, Sydney, NSW, Australia

[12]School of Life and Environmental Sciences, Faculty of Science, University of Sydney, Camperdown, New South Wales, Australia

Emails of all authors: richard.edwards@unsw.edu.au (RJE), d.enosi@unsw.edu.au (DET), t.amos@unsw.edu.au (TGA), omeally@gmail.com (DO), m.richardson@deakin.edu.au (MFR), t.russell@unsw.edu.au (TLR), mvallinoto@cibio.up.pt (MV), miguel.carneiro@cibio.up.pt (MC), nferrand@cibio.up.pt (NF), m.wilkins@unsw.edu.au (MRW), fsequeira@cibio.up.pt (FS), l.rollins@unsw.edu.au (LAR), edward.holmes@sydney.edu.au (ECH), rick.shine@sydney.edu.au (RS), p.white@unsw.edu.au (PAW).

*Corresponding author address: School of Biotechnology and Biomolecular Sciences, University of New South Wales, Sydney, NSW, Australia. Tel: +61-293853780; Email: p.white@unsw.edu.au

Peter White ORCID ID: 0000-0002-6046-9631

Abstract

Background: The cane toad (*Rhinella marina* formerly *Bufo marinus*) is a species native to Central and South America that has spread across many regions of the globe. Cane toads are known for their rapid adaptation and deleterious impacts on native fauna in invaded regions. However, despite an iconic status, there are major gaps in our understanding of cane toad genetics. The availability of a genome would help to close these gaps and accelerate cane toad research. **Findings:** We report a draft genome assembly for *R. marina*, the first of its kind for the Bufonidae family. We used a combination of long read PacBio RS II and short read Illumina HiSeq X sequencing to generate a total of 359.5 Gb of raw sequence data. The final hybrid assembly of 31,392 scaffolds was 2.55 Gb in length with a scaffold N50 of 168 kb. BUSCO analysis revealed that the assembly included full length or partial fragments of 90.6% of tetrapod universal single-copy orthologs (n=3950), illustrating that the gene-containing regions have been well-assembled. Annotation predicted 25,846 protein coding genes with similarity to known proteins in SwissProt. Repeat sequences were estimated to account for 63.9% of the assembly. **Conclusion:** The *R. marina* draft genome assembly will be an invaluable resource that can be used to further probe the biology of this invasive species. Future analysis of the genome will provide insights into cane toad evolution and enrich our understanding of their interplay with the ecosystem at large.

Keywords: cane toad; *Rhinella marina;* sequencing; hybrid assembly; genome; annotation

Data Description

Introduction

The cane toad (*Rhinella marina* formerly *Bufo marinus*) (Figure 1) is a true toad (Bufonidae) native to Central and South America that has been introduced to many areas across the globe [1]. Since its introduction into Queensland in 1935, the cane toad has spread widely and now occupies more than 1.2 million square kilometres of the Australian continent, fatally poisoning predators like the northern quoll, freshwater crocodiles, and several species of native lizards and snakes [1-5]. The ability of cane toads to kill predators with toxic secretions has contributed to the success of their invasion [1]. To date, research on cane toads has focused primarily on ecological impacts, rapid evolution of phenotypic traits, and population genetics using neutral markers [6, 7], with limited knowledge of the genetic changes that allow the cane toad to thrive in the Australian environment [8-11]. A reference genome will be useful for studying loci subject to rapid evolution and could provide valuable insights into how invasive species adapt to new environments. Amphibian genomes have a preponderance of repetitive DNA [12, 13], confounding assembly with the limited read lengths of first- and second-generation sequencing technologies. Here, we employ a hybrid assembly of PacBio long reads and Illumina short reads (Figure 2) to overcome assembly challenges presented by the repetitive nature of the cane toad genome. Using this approach, we assembled a draft genome of *R. marina* that is comparable in contiguity and completeness to other published anuran genomes [14-17]. We used our previously published transcriptomic data [18] and other published anuran sequences to annotate the genome. Our draft cane toad assembly will serve as a reference for genetic and evolutionary studies, and provides a template for continued refinement with additional sequencing efforts.

Sample collection, library construction and sequencing

Adult female cane toads were collected by hand from Forrest River in Oombulgurri, WA (15.1818ºS, 127.8413ºE) in June 2015. Toads were placed in individual damp cloth bags and transported by plane to Sydney, NSW before they were anaesthetised by refrigeration for four hours and killed by subsequent freezing. High-molecular weight genomic DNA (gDNA) was extracted from the liver of a single female

using the genomic-tip 100/G kit (Qiagen, Hilden, Germany). This was performed with supplemental RNase (Astral Scientific, Taren Point, Australia) and proteinase K (NEB, Ipswich, MA, USA) treatment, as per the manufacturer's instructions. Isolated genomic DNA was further purified using AMPure XP beads (Beckman Coulter, Brea, CA, USA) to eliminate sequencing inhibitors. DNA quantity was assessed using the Quanti-iT PicoGreen dsDNA kit (Thermo Fisher Scientific, Waltham, MA, USA), DNA purity was calculated using a Nanodrop spectrophotometer (Thermo Fisher Scientific), and molecular integrity assessed by pulse-field gel electrophoresis.

For short read sequencing, a paired-end library was constructed from the gDNA using the TruSeq PCR-free library preparation kit (Illumina, San Diego, CA, USA). Insert sizes ranged between 200-800 bp. This library was sequenced (2 × 150 bp) on the HiSeq X Ten platform (Illumina) to generate approximately 282.9 Gb of raw data (Table 1). Illumina short sequencing reads were assessed for quality using FastQC v0.10.1 [19]. Low quality reads filtered were trimmed using Trimmomatic v0.36 [20] with a Q30 threshold (LEADING:30, TRAILING:30, SLIDINGWINDOW:4:30) and a minimum 100 bp read length, leaving 64.9% of the reads generated, of which 75.2% were in retained read pairs.

For long read sequencing, we utilised the single-molecule real time (SMRT) sequencing technology (Pacific Biosciences, Menlo Park, CA, USA). Four SMRTbell libraries were prepared from gDNA using the SMRTBell template preparation kit 1.0 (Pacific Biosciences). To increase subread length, either 15-50 kb or 20-50 kb BluePippin size selection (Sage Science, Beverly, MA, USA) was performed on each library. Recovered fragments were sequenced using P6C4 sequencing chemistry on the RS II platform (240 min movie time). The four SMRTbell libraries were sequenced on a total of 97 SMRT cells to generate 7,745,233 subreads for a total of 76.6 Gb of raw data. Collectively, short and long read sequencing produced around 359.5 Gb of data (Table 1).

Genome assembly

We employed a hybrid *de novo* whole genome assembly strategy, combining both short read and long read data. Trimmed Q30-filtered short reads were *de novo* assembled with ABySS v1.3.6 [21] using k=64 and default parameters (contig N50 = 583 bp) (Table 2). Long sequence reads were *de novo*

assembled using the program DBG2OLC [22] (k 17 AdaptiveTh 0.0001 KmerCovTh 2 MinOverlap 20 RemoveChimera 1) (contig N50 = 167.04 kbp) (Table 2). Following this, both assemblies were merged together using the hybrid assembler ('sparc') tool of DBG2OLC with default parameters, combining the contiguity of the long read data with the improved accuracy of the high coverage Illumina assembly. This hybrid assembly (v2.0) was twice 'polished' to remove errors. In the first round, the Q30 trimmed Illumina reads were mapped to the hybrid assembly with bowtie v2.2.9 [23] and filtered for proper pairs using samtools v1.3.1 [24]. Scaffolds were polished with Pilon v1.21 [25] to generate the second iteration of the assembled genome (v2.1). In the second round, PacBio subreads were mapped to assembly v2.1 for error correction using SMRT analysis software (Pacific Biosciences): PacBio subreads for each library were converted to BAM format with bax2bam v0.0.08 and aligned to the genome using pbalign v.0.3.0. BAM alignment files were combined using samtools merge v1.3.1 and the scaffolds polished with Arrow v2.1.0 to generate the final genome assembly (v2.2). Our final draft assembly of the cane toad genome (v2.2) has 31,392 scaffolds with an N50 of 167 kb (Table 2). The GC content (43.23%) is within 1% of the published estimate of 44.17%, determined by flow cytometry [26].

Assessment of genome completeness

BUSCO [27] analysis of conserved single copy orthologues is widely used as a proxy for genome completeness and accuracy. While direct comparisons are only truly valid within an organism, comparing BUSCO scores to genomes from related organisms provides a useful benchmark. We ran BUSCO v2.0.1 (short mode, lineage tetrapoda_odb9, BLAST+ v2.2.31 [28], HMMer v3.1b2 [29], AUGUSTUS v3.2.2 [30], EMBOSS v6.5.7 [31]) on each of our assemblies, along with four published anuran genomes (Figure 3, Table 2). The hybrid assembly combined the completeness of the long read assembly with the accuracy of the short read assembly, providing an enormous boost in BUSCO completeness from less than 50% full and partial orthologs to over 90%. Error correction through pilon and arrow polishing had a positive effect on the BUSCO measurement of genome completeness, with an increase of 7.8% in the number of full and partial orthologs between v2.0 and 2.2. For the polished assembly (v2.2), 3279 (83.0%) of the 3950 ultra-conserved tetrapod genes were complete, 296 (7.5%)

were fragmentary and 375 (9.5%) were missing. It should be noted that these numbers mask some underlying complexity of BUSCO assessments; aggregate improvements in BUSCO scores with polishing include some losses as well as gains. Taking the best rating for each BUSCO in v2.0, v2.1 or v2.2 reduces the number of missing BUSCO genes to 326 (8.3%) and increases the complete number to 3366 (85.2%) (Figure 3, "*R. marina* (combined)"). This is explored further in the "Genome annotation and prediction" section, below. Overall, BUSCO metrics indicate that our draft *R. marina* genome is approaching the quality and completeness of the widely used anuran amphibian reference genomes for *X. laevis* (v9.2) [17] and *X. tropicalis* (v.9.1) [16] and compares well to the recently published neobatrachian genomes of *Nanorana parkeri* (v2) [15] and *Lithobates catesbeianus* (v2.1) [14].

Estimation of *R. marina* genome size

Previous reports have estimated the size of the cane toad genome from 3.98-5.65 Gb using either densitometry or flow cytometry analysis of stained nuclei within erythrocytes, hepatocytes and renal cells [26, 32-38]. We employed two alternative strategies to measure the genome size, using short read k-mer distributions and qPCR of single copy genes. K-mer frequencies were calculated for both raw and trimmed Q30-filtered paired-end short reads (Table 1) with Jellyfish v2.2.3 [39] using k=21 and k=23, and a maximum k-mer count of 10,000. K-mer distributions were analysed using GenomeScope [40] with mean read lengths of 148 bp (raw) or 141 bp (Q30) and k-mer coverage cut-offs of 1000 and 10,000 (Table 3, Figure 4). GenomeScope gave genome size estimates ranging from 1.77 Gb to 2.30 Gb with the raw reads giving consistently larger estimates (1.85 Gb to 2.30 Gb) than the trimmed and filtered reads (1.77 Gb to 2.10 Gb). Estimates of the unique (single copy) region of the genome were more consistent, ranging from 1.31 Gb to 1.46 Gb, with k=23 estimates 99 Mb (raw) or 80 Mb (Q30) higher than k=21. Increasing the GenomeScope maximum k-mer coverage threshold had the greatest effect on predicted genome size, increasing repeat length estimates by 274 Mb to 385 Mb. GenomeScope explicitly models heterozygous diploid kmer distributions, which should make it robust to the additional challenge of sequencing a wild animal. However, GenomeScope predictions are affected by non-uniform repeat distributions and this difference could indicate high copy number

repeats in the genome that are difficult to model accurately. It is possible that high frequency repeats with raw sequencing counts exceeding 10,000 are resulting in an underestimate of total repeat length and therefore genome size, compared to the previous densitometry and flow cytometry predictions.

In the second approach, the *zfp292* (zinc finger protein 292) gene was selected from our BUSCO analysis as a single-copy target for genome estimation by qPCR [41]. First, PCR was used to amplify a 326 bp region of *zfp292* (scaffold 6589, position 345,750-346,075) in a 25 µL reaction that contained 50 ng of gDNA, 200 µM dNTP, 0.625 units of Taq polymerase (Invitrogen), 10 × Taq polymerase buffer (Invitrogen) and 0.4 µM of each primer (Table S1). The amplicon was cloned into the pGEM-T Easy vector (Promega, Madison, WI, USA) and the resultant plasmid was linearised with NdeI before being serially diluted to generate a qPCR standard (10^1-10^9 copies/µL). To amplify a smaller region (120 bp) within *zfp292* (scaffold 6589, position 345,858-345,977) gDNA (10-25 ng) or 1 µL of the diluted standards were used as a template for a 20 µL qPCR reaction containing 2 × iTaq SYBR Green mastermix (BioRad, Hercules, CA, USA) and 0.5 µM of each primer (Table S1). Cycle threshold values obtained for each plasmid dilution were used to generate a standard curve and infer the number of *zfp292* amplicons generated from the template gDNA of known quantity. Genome sizes were generated from the formulae outlined by [41] and the average of two estimates (2.81 Gb and 1.94 Gb) were used to obtain a genome size of 2.38 Gb. This genome size provides an estimated combined 151X sequencing coverage (119X Illumina and 32X PacBio) (Table 4).

Our genome size estimation of 1.98 to 2.38 Gbp is smaller than the 2.55 Gbp assembly size, and differs significantly from previously published estimates of 4 Gbp or more for this species. We suggest this is a result of the repetitive nature of the genome (see below). Given this is the first estimate of the cane toad genome size using either k-mer or qPCR analysis, further investigations are required to more clearly understand the discrepancy in our estimates with respect to published genome sizes. Here we estimate the depth of sequencing coverage using both sequence-based and cytometric genome size measures (Table 4).

Genome annotation and gene prediction

Annotation of the draft genome was performed using MAKER2 v2.31.6 [42], BLAST+ v2.2.31 [28], AUGUSTUS v3.2.2 [30], Exonerate v2.2.0 [43], RepeatMasker v4.0.6 [44] (DFAM [45], Library Dfam_1.2; RMLibrary v20150807), RepeatModeler v1.0.8 [46] and SNAP v2013-11-29 [47] using all SwissProt protein sequences (downloaded 2017-02-23)[48] . AUGUSTUS was trained using BUSCO v2.0.1 (long mode, lineage tetrapoda_odb9) and a multi-tissue reference transcriptome we previously generated from tadpoles and six adult cane toad tissues [18] (available from GigaDB [49], Genbank accession PRJNA383966). Whole-tadpoles and the brain, liver, spleen, muscle, ovary and testes of adult toads from Australia and Brazil were used to prepare cDNA libraries for the multi-tissue transcriptome sequencing. After the initial training run, two further iterations of MAKER2 were run using HMMs from SNAP training created from the previous run. Functional annotation of protein-coding genes predicted by MAKER2 were generated using Interproscan 5.25-64.0, with the following settings: -dp -t p -pa -goterms -iprlookup -appl TIGRFAM, SFLD, Phobius, SUPERFAMILY, PANTHER, Gene3D, Hamap, ProSiteProfiles, Coils, SMART, CDD, PRINTS, ProSitePatterns, SignalP_EUK, Pfam, ProDom, MobiDBLite, PIRSF, TMHMM. BLAST+ v2.6.0 [28] was used to annotate predicted genes using all Swissprot proteins (release 2017_08, downloaded 2017-09-01) [48] using the following settings: -evalue 0.000001 -seg yes -soft_masking true -lcase_masking -max_hsps 1.

In total, 58,302 protein-coding genes were predicted by the MAKER pipeline with an average of 5.3 exons and 4.3 introns per gene (Table 5). Of these, 5,225 are single exon genes, giving 4.7 introns per multi-exon gene with an average intron length of 4.08 kb. Predicted coding sequences make up 2.38% of the assembly. MAKER predicted considerably more than the approximately twenty thousand genes expected for a typical vertebrate genome. There are two likely explanations for this: (1) artefactual duplications in the genome assembly, either through under-assembly or legitimate assembly of two heterozygous diploid copies; (2) over-prediction of proteins during genome annotation, including pseudogenes with high homology to functional genes, proteins from transposable elements or other repeats, and multiple fragments of open reading frames (ORFs) from the same gene (due to fragmentation of the genome) and lncRNA genes that have been incorrectly assigned a coding sequence.

Of the 3,279 complete BUSCO genes identified (Table 2), only 85 (2.59%) were duplicated. This suggests that there is not widespread duplication in the assembly. Only 25,846 predicted genes were annotated as similar to known proteins in SwissProt, with the remaining 32,456 predictions "of unknown function". This is consistent with over-prediction being the primary cause of inflated gene numbers. Poor quality protein predictions are generally shorter (generated from fragmented or random ORFs) and have a larger Annotation Edit Distance (AED) when compared to real proteins. Consistent with this, the predicted proteins of unknown function are shorter in sequence (median length 171 aa) to those with Swissprot hits (median length 388 aa) (Figure 5A) and have a greater AED (median 0.37 versus 0.2) (Figure 5B). To investigate this further, predicted transcript and protein sequences were searched against the published *de novo* assembled transcriptome [18] using BLAST+ v2.2.31 [28] blastn or tblastn (top 10 hits, e-value < 10^{-10}) and compiled with GABLAM v2.28.3 [50]. For 56.5% of proteins with functional annotation, 95%+ of the protein length mapped to the top transcript hit (Table 6). Only 27.1% of unknown proteins had 95%+ coverage in the top transcript hit, which is again consistent with over-prediction. We also reanalysed the multi-tissue RNA-Seq data from Richardson *et al.* [18] by mapping the reads onto the MAKER predicted transcripts. Filtered reads (adaptor sequences and reads with avg. Phred < 30 removed) were mapped with Salmon v0.8.0 [51] (Quasi-mapping default settings, IU libtype parameter). Read counts were converted into transcripts per million (TPM) by normalising by transcript length, dividing by the sum of the length-normalised read counts, and then multiplying by one million. We observed lower expression levels overall in the "unknown" set (Figure 6). With the caveat that real proteins may have very low expression, this is also consistent with the "unknown" gene set containing false annotations. Further review of the predicted protein descriptions revealed 4,357 with likely origins in transposable elements (including 4,114 LINE-1 ORFs) and 215 from viruses. However, many of these may be bona fide functional members of the cane toad proteome: 1,447 (33.2%) "transposon" and 151 (70.2%) of "viral" transcripts had support for expression > 1 TPM.

To investigate the role of fragmented ORFs, we downloaded the Quest For Orthologues (QFO) reference proteomes (QFO 04/18) [52] and used BLAST+ v2.2.31 [28] blastp (e-value < 10^{-7}) to identify the top hit for each predicted protein in (a) all eukaryote reference proteomes, and (b) the *Xenopus*

tropicalis reference proteome. BLAST results were converted into global coverage with GABLAM v2.28.3 [50]. As expected, the vast majority (99.6%) of "similar" proteins had a blastp hit the QFO proteomes (data not shown). Perhaps surprisingly, nearly two thirds (66.5%) of "unknown" proteins also had a blastp hit, but these had lower coverage of the reference proteins than did proteins in the "similar" class (data not shown). A "combined coverage" score was calculated for each protein, taking the minimum percentage coverage of either the query protein or its top QFO hit. This metric was related to annotation quality, showing an inverse relationship with AED (data not shown). Excluding proteins with annotation indicating possible viral or transposable element origin, 45.7% of "similar" proteins and 96.8% of "unknown" proteins had the same closest *X. tropicalis* blastp hit as another predicted protein. Consistent with this being related to gene fragmentation, there was a negative relationship between the number of cane toad proteins sharing a given *X. tropicalis* top hit, and how much of the *X. tropicalis* hit was covered by each cane toad protein. Nevertheless, it is likely that some of these protein fragments represent allelic variants that have been redundantly assembled.

We ran BUSCO v2.0.1 (short mode, lineage tetrapoda_odb9, BLAST+ v2.2.31 [28], HMMer v3.1b2 [29], AUGUSTUS v3.2.2 [30], EMBOSS v6.5.7 [31]) on the MAKER2 transcriptome and proteome and retained the most complete rating for each gene (Figure 7A, Table S2, "Annotation"). MAKER annotation had fewer missing BUSCO genes than the v2.2 assembly (314 vs 375) but many more fragmented (561 vs 296). Equivalent BUSCO analysis of the Richardson *et al.* transcriptome [18] was only missing 296 genes. However, as seen with the assembly versions, these values mask hidden complexity. Combined BUSCO analysis of our hybrid assembly (v2.0, v2.1, v2.2) and annotation, revealed only 181 missing genes (Figure 7A, Table S2, "GigaDB"). Furthermore, >50% of the 279 genes "Missing" in the transcriptome are found in the genome and/or its annotation (Figure 7B, Table S2). When the transcriptome and our genome are combined, only 68 BUSCO genes (1.7%) are "Missing" and 3845 (97.3%) are "Complete" (Figure 7B, Table S2, "CaneToad"). This highlights the usefulness of our assembly, and illustrates the complementary nature of genome and transcriptome data: the former is more comprehensive but more difficult to assemble and annotate, whereas the latter is easier to assemble into full-length coding sequences but will miss some tissue-specific and lowly

expressed genes. Some of the remaining "Missing" BUSCO genes may be present but too fragmented to reach the score threshold.

Future work will be needed to improve the quality of gene annotation. We have included all of the MAKER2 predictions in our annotation and a full table of protein statistics and top blastp hits from this analysis for further biological analyses (Table S3). Annotation has also been made available via a WebApollo [53] genome browser (http://edwapollo.babs.unsw.edu.au/) and an associated search tool (http://www.slimsuite.unsw.edu.au/servers/apollo.php). This will facilitate community curation and annotation of genes of interest. For researchers who would like to use cane toad proteins in general evolutionary analyses, we have also created a "high quality" dataset of 6,580 protein-coding genes with an AED no greater than 0.25 and at least 90% reciprocal coverage of its top QFO blastp hit, excluding possible viral and transposon proteins, available from the *GigaScience* database.

Phylogenetic analysis of high quality proteins

To further validate the high-quality protein data set, GOPHER [54] v3.4.2 was used to predict orthologues for each protein. QFO (04/18) [52] eukaryotic reference proteomes were supplemented with Uniprot Reference proteomes for *Lithobates catesbeiana* (UP000228934) [14] and *Xenopus laevis* (UP000186698) [17] and the annotated protein sequences of *Nanorana parkeri* v2 [15]. GOPHER orthologues were predicted with default settings based on a modified mutual best hit algorithm that accounts for one-to-many or many-to-many orthologous relationships and retains the closest orthologue from each species. The closest orthologues were aligned with MAFFT [55] v7.310 (default settings) and phylogenetic trees inferred with IQ-TREE [56] v1.6.1 (default settings) for alignments containing at least three sequences. Phylogenetic trees were inferred in this manner for 6,417 of the 6,580 high quality proteins. A supertree was then constructed from the 6,417 individual protein trees using CLANN [57] v4.2.2 (DFIT Most Similar Supertree Algorithm) (Figure 8, Figure S1). Branch consistency was calculated for each branch as the proportion of source trees with taxa either side of the branch that have no conflicts in terms of the placement of those taxa. The supertree supports the known phylogeny for amphibians used in this study, giving additional confidence in the quality and utility of these protein annotations. All alignments and trees are available in supplementary data via the *GigaScience* database.

Repeat identification and analysis

The cane toad genome has proven very difficult to assemble using short reads alone, which suggests a high frequency of repetitive sequences, as for other amphibians [12, 13]. RepeatMasker annotations from the MAKER pipeline support this interpretation, with over 4.1 million repeat sequences detected, accounting for 63.9% of the assembly (Table 5). The mean repeat length is 406 bp, which exceeds the Illumina read length used in our study (mean 140.6 bp paired-end). This makes short-read assembly of these regions difficult, as reflected by the poor ABySS contiguity (contig N50 = 583 bp, Table 2), and emphasises the need for long read data in this organism. The most abundant class of repeat elements are of unknown type (1.61 million elements covering 32.28% of the assembly), with DNA transposons the most abundant known class of element (817,262 repeats; 19.17% coverage). Of these, the most abundant are of the hAT-Ac (231,332 copies) and TcMar-Tc1 (226,145 copies) superfamilies (Table S4). Accounting for overlaps between repeat and gene features, 18.7% of the assembly (479,397,014 bp) has no annotation (Figure 9).

Conclusion

This draft genome assembly will be an invaluable tool for advancing knowledge of anuran biology, genetics and the evolution of invasive species. Furthermore, we envisage these data will facilitate the development of biocontrol strategies that reduce the impact of cane toads on native fauna.

Availability of supporting data

Raw genomic sequencing data (Illumina and PacBio) and assembled scaffolds have been deposited in the ENA with the study accession PRJEB24695 and assembly accession GCA_900303285. The genome assembly and annotation are also available in the *GigaScience* database, and via a WebApollo [53] genome browser and an associated search tool [59]. Data further supporting this work is available in the GigaScience database, GigaDB [60].

List of abbreviations

AED: annotation edit distance; BUSCO: Benchmarking Universal Single-Copy Orthologs; BLAST: Basic Local Alignment Search Tool, qPCR: quantitative polymerase chain reaction, HMM: hidden Markov model, CDS: coding sequence; bp: base pair; gDNA: genomic DNA; ORF: open reading frame; QFO: Quest For Orthologues; SMRT: single-molecule real time; SINE: short interspersed nuclear element; LINE: long interspersed nuclear element, LTR: long terminal repeat; TE: transposable elements; TPM: transcripts per million; UTR: untranslated region, s.f.: significant figure

Additional files

Figure S1. Phylogenetic supertree constructed from phylogenetic trees for 6,417 high confidence cane toad proteins.

Table S1. Primers used for genome size estimation by single copy gene qPCR.

Table S2. Individual and combined full BUSCO gene ratings for cane toad assemblies, annotation, transcriptome.

Table S3. Sequence statistics, top BLAST hits, and classification for MAKER2 annotations.

Table S4. RepeatMasker statistics broken down by repeat category.

Ethics approval and consent to participate

All experimentation was performed under the approval of the University of Sydney Animal Ethics Committee.

Consent for publication

Not applicable

Competing interests

The authors declare that they have no competing interests.

Funding

This work was supported by the Cane Toad Genome Consortium and Bioplatforms Australia. Funding awarded to L.A.R. and R.S was provided by the Australian Research Council (DP160102991). L.A.R. is funded by ARC DECRA Fellowship (DE150101393). R.S. is funded by ARC Australian Laureate Fellowship (FL120100074). E.C.H. is funded by ARC Australian Laureate Fellowship (FL170100022). M.R.W. and the Ramaciotti Centre for Genomics acknowledge infrastructure funding from the Australian Research Council (LE150100031), the Australian Government NCRIS scheme administered by Bioplatforms Australia and the New South Wales Government RAAP scheme. M.V. is supported by a CNPq fellowship (302892/2016-8). D.E.T. was supported through an Australian Government Research Training Program (RTP) scholarship.

Author's contributions

P.A.W coordinated the project. P.A.W, R.S, E.C.H, L.A.R, R.J.E, M.W. designed the study. P.A.W, R.S, E.C.H, L.A.R, R.J.E and F.S funded the project. R.S provided the cane toad samples. D.E.T performed the genomic DNA extraction, PCR experiments and data analysis. T.L.R performed the sequencing. R.J.E and T.G.A performed the genome assemblies and primary data analysis. D.O and T.G.A. performed the genome annotation. R.J.E, D.E.T, T.G.A and P.A.W and wrote the manuscript. All authors edited and approved the final manuscript.

Acknowledgements

We thank the Ramaciotti Centre for Genomics, UNSW and the Kinghorn Centre for Clinical Genomics at the Garvan Institute of Medical Research who performed the sequencing in this study. This project was undertaken with the assistance of resources and services from the National Computational Infrastructure (NCI), which is supported by the Australian Government. This project used computational resources from Intersect, which were in part supported by the Australian Research Council. The authors acknowledge UNSW Science's high performance computing cluster (Katana), the UNSW School of Biotechnology and Biomolecular Sciences high memory node, the Sydney Informatics Hub and the University of Sydney's high performance computing cluster (Artemis) for

providing the high performance computing resources that have contributed to the research results reported within this paper. We thank the members of the UNSW Science Faculty Computing unit, particularly Duncan Smith, for all his help getting software installed and running on the katana computer cluster. We thank Dr Andrew Kelly for early discussions on cane toad genome size and technical assistance with gDNA extractions.

References

1. Shine R. The ecological impact of invasive cane toads (Bufo marinus) in Australia. The Quarterly Review of Biology. 2010;85 3:253-91.
2. Phillips BL, Brown GP, Greenlees M, Webb JK and Shine R. Rapid expansion of the cane toad (Bufo marinus) invasion front in tropical Australia. Austral Ecology. 2007;32 2:169-76.
3. Phillips BL, Brown GP and Shine R. Assessing the potential impact of cane toads on Australian snakes. Conservation Biology. 2003;17 6:1738-47.
4. Smith JG and Phillips BL. Toxic tucker: the potential impact of cane toads on Australian reptiles. Pacific Conservation Biology. 2006;12 1:40-9.
5. Urban MC, Phillips BL, Skelly DK and Shine R. The cane toad's (Chaunus [Bufo] marinus) increasing ability to invade Australia is revealed by a dynamically updated range model. Proceedings of the Royal Society of London B: Biological Sciences. 2007;274 1616:1413-9.
6. Slade R and Moritz C. Phylogeography of Bufo marinus from its natural and introduced ranges. Proceedings of the Royal Society of London B: Biological Sciences. 1998;265 1398:769-77.
7. Sequeira F, Sodré D, Ferrand N, Bernardi JA, Sampaio I, Schneider H, et al. Hybridization and massive mtDNA unidirectional introgression between the closely related Neotropical toads Rhinella marina and R. schneideri inferred from mtDNA and nuclear markers. BMC evolutionary biology. 2011;11 1:264.
8. Rollins LA, Richardson MF and Shine R. A genetic perspective on rapid evolution in cane toads (Rhinella marina). Molecular Ecology. 2015;24 9:2264-76.
9. Estoup A, Baird SJ, Ray N, Currat M, CORNUET J, Santos F, et al. Combining genetic, historical and geographical data to reconstruct the dynamics of bioinvasions: application to the cane toad Bufo marinus. Molecular ecology resources. 2010;10 5:886-901.
10. Trumbo DR, Epstein B, Hohenlohe PA, Alford RA, Schwarzkopf L and Storfer A. Mixed population genomics support for the central marginal hypothesis across the invasive range of the cane toad (Rhinella marina) in Australia. Molecular ecology. 2016;25 17:4161-76.
11. Leblois R, Rousset F, Tikel D, Moritz C and Estoup A. Absence of evidence for isolation by distance in an expanding cane toad (Bufo marinus) population: an individual-based analysis of microsatellite genotypes. Molecular Ecology. 2000;9 11:1905-9.
12. Bozzoni I and Beccari E. Clustered and interspersed repetitive DNA sequences in four amphibian species with different genome size. Biochimica et Biophysica Acta (BBA)-Nucleic Acids and Protein Synthesis. 1978;520 2:245-52.
13. Olmo E. Genome variations in the transition from amphibians to reptiles. Journal of molecular evolution. 1991;33 1:68-75.
14. Hammond SA, Warren RL, Vandervalk BP, Kucuk E, Khan H, Gibb EA, et al. The North American bullfrog draft genome provides insight into hormonal regulation of long noncoding RNA. Nature Communications. 2017;8 1:1433.
15. Sun Y-B, Xiong Z-J, Xiang X-Y, Liu S-P, Zhou W-W, Tu X-L, et al. Whole-genome sequence of the Tibetan frog Nanorana parkeri and the comparative evolution of tetrapod genomes. Proceedings of the National Academy of Sciences. 2015;112 11:E1257-E62.
16. Hellsten U, Harland RM, Gilchrist MJ, Hendrix D, Jurka J, Kapitonov V, et al. The genome of the Western clawed frog Xenopus tropicalis. Science. 2010;328 5978:633-6.

17. Session AM, Uno Y, Kwon T, Chapman JA, Toyoda A, Takahashi S, et al. Genome evolution in the allotetraploid frog Xenopus laevis. Nature. 2016;538 7625:336.
18. Richardson MF, Sequeira F, Selechnik D, Carneiro M, Vallinoto M, Reid JG, et al. Improving amphibian genomic resources: a multi-tissue reference transcriptome of an iconic invader. GigaScience. 2017;7 1:1-7.
19. Andrews S. FastQC: a quality control tool for high throughput sequence data. 2010.
20. Bolger AM, Lohse M and Usadel B. Trimmomatic: a flexible trimmer for Illumina sequence data. Bioinformatics. 2014;30 15:2114-20.
21. Simpson JT, Wong K, Jackman SD, Schein JE, Jones SJ and Birol I. ABySS: a parallel assembler for short read sequence data. Genome research. 2009;19 6:1117-23.
22. Ye C, Hill CM, Wu S, Ruan J and Ma ZS. DBG2OLC: efficient assembly of large genomes using long erroneous reads of the third generation sequencing technologies. Scientific reports. 2016;6:31900.
23. Langmead B and Salzberg SL. Fast gapped-read alignment with Bowtie 2. Nature methods. 2012;9 4:357-9.
24. Li H, Handsaker B, Wysoker A, Fennell T, Ruan J, Homer N, et al. The sequence alignment/map format and SAMtools. Bioinformatics. 2009;25 16:2078-9.
25. Walker BJ, Abeel T, Shea T, Priest M, Abouelliel A, Sakthikumar S, et al. Pilon: an integrated tool for comprehensive microbial variant detection and genome assembly improvement. PloS one. 2014;9 11:e112963.
26. Vinogradov AE. Genome size and GC-percent in vertebrates as determined by flow cytometry: The triangular relationship. Cytometry Part A. 1998;31 2:100-9.
27. Simão FA, Waterhouse RM, Ioannidis P, Kriventseva EV and Zdobnov EM. BUSCO: assessing genome assembly and annotation completeness with single-copy orthologs. Bioinformatics. 2015;31 19:3210-2.
28. Camacho C, Coulouris G, Avagyan V, Ma N, Papadopoulos J, Bealer K, et al. BLAST+: architecture and applications. BMC bioinformatics. 2009;10 1:421.
29. Mistry J, Finn RD, Eddy SR, Bateman A and Punta M. Challenges in homology search: HMMER3 and convergent evolution of coiled-coil regions. Nucleic acids research. 2013;41 12:e121-e.
30. Keller O, Kollmar M, Stanke M and Waack S. A novel hybrid gene prediction method employing protein multiple sequence alignments. Bioinformatics. 2011;27 6:757-63.
31. Rice P, Longden I and Bleasby A. EMBOSS: the European molecular biology open software suite. Elsevier Current Trends, 2000.
32. Bachmann K. Specific nuclear DNA amounts in toads of the genus Bufo. Chromosoma. 1970;29 3:365-74.
33. Camper J, Ruedas L, Bickham J and Dixon J. The relationship of genome size with developmental rates and reproductive strategies in five families of neotropical bufonoid frogs. Life Sci Adv. 1993;12:79-87.
34. Bachmann K. Nuclear DNA and developmental rate in frogs. Quarterly Journal of the Florida Academy of Sciences. 1972;35 4:225-31.
35. Chipman AD, Khaner O, Haas A and Tchernov E. The evolution of genome size: what can be learned from anuran development? Journal of Experimental Zoology Part A: Ecological Genetics and Physiology. 2001;291 4:365-74.
36. Griffin C, Scott D and Papworth D. The influence of DNA content and nuclear volume on the frequency of radiation-induced chromosome aberrations in Bufo species. Chromosoma. 1970;30 2:228-49.
37. Goin OB, Goin CJ and Bachmann K. DNA and amphibian life history. Copeia. 1968:532-40.
38. MacCulloch RD, Upton DE and Murphy RW. Trends in nuclear DNA content among amphibians and reptiles. Comparative Biochemistry and Physiology Part B: Biochemistry and Molecular Biology. 1996;113 3:601-5.
39. Marçais G and Kingsford C. A fast, lock-free approach for efficient parallel counting of occurrences of k-mers. Bioinformatics. 2011;27 6:764-70.

40. Vurture GW, Sedlazeck FJ, Nattestad M, Underwood CJ, Fang H, Gurtowski J, et al. GenomeScope: fast reference-free genome profiling from short reads. Bioinformatics. 2017;33 14:2202-4.
41. Wilhelm J, Pingoud A and Hahn M. Real-time PCR-based method for the estimation of genome sizes. Nucleic Acids Research. 2003;31 10:e56-e.
42. Holt C and Yandell M. MAKER2: an annotation pipeline and genome-database management tool for second-generation genome projects. BMC bioinformatics. 2011;12 1:491.
43. Slater GSC and Birney E. Automated generation of heuristics for biological sequence comparison. BMC bioinformatics. 2005;6 1:31.
44. Smit AFA, Hubley R and Green P. 2013–2015. RepeatMasker Open-4.0. 2013. http://www.repeatmasker.org/.
45. Hubley R, Finn RD, Clements J, Eddy SR, Jones TA, Bao W, et al. The Dfam database of repetitive DNA families. Nucleic acids research. 2015;44 D1:D81-D9.
46. Smit AFA and Hubley R. 2008–2015. RepeatModeler Open-1.0. 2008. http://www.repeatmasker.org/.
47. Korf I. Gene finding in novel genomes. BMC bioinformatics. 2004;5 1:59.
48. Apweiler R, Bairoch A, Wu CH, Barker WC, Boeckmann B, Ferro S, et al. UniProt: the Universal Protein knowledgebase. Nucleic Acids Research. 2004;32 suppl_1:D115-D9. doi:10.1093/nar/gkh131.
49. Richardson MF, Sequeira F, Selechnik D, Carneiro M, Vallinoto M, Reid JG, et al. Supporting data for "Improving amphibian genomic resources: a multitissue reference transcriptome of an iconic invader." Giga-Science Database 2017. http://dx.doi.org/10.5524/100374.
50. Davey NE, Shields DC and Edwards RJ. SLiMDisc: short, linear motif discovery, correcting for common evolutionary descent. Nucleic acids research. 2006;34 12:3546-54.
51. Patro R, Duggal G, Love MI, Irizarry RA and Kingsford C. Salmon provides fast and bias-aware quantification of transcript expression. Nature methods. 2017;14 4:417.
52. Altenhoff AM, Boeckmann B, Capella-Gutierrez S, Dalquen DA, DeLuca T, Forslund K, et al. Standardized benchmarking in the quest for orthologs. Nature methods. 2016;13 5:425.
53. Lee E, Helt GA, Reese JT, Munoz-Torres MC, Childers CP, Buels RM, et al. Web Apollo: a web-based genomic annotation editing platform. Genome biology. 2013;14 8:R93.
54. Davey NE, Edwards RJ and Shields DC. The SLiMDisc server: short, linear motif discovery in proteins. Nucleic acids research. 2007;35 suppl_2:W455-W9.
55. Katoh K and Standley DM. MAFFT multiple sequence alignment software version 7: improvements in performance and usability. Molecular biology and evolution. 2013;30 4:772-80.
56. Nguyen L-T, Schmidt HA, von Haeseler A and Minh BQ. IQ-TREE: a fast and effective stochastic algorithm for estimating maximum-likelihood phylogenies. Molecular biology and evolution. 2014;32 1:268-74.
57. Creevey C and McInerney JO. Clann: investigating phylogenetic information through supertree analyses. Bioinformatics. 2004;21 3:390-2.
58. Rambaut A. FigTree v1.4. Molecular evolution, phylogenetics and epidemiology Edinburgh, UK: University of Edinburgh, Institute of Evolutionary Biology. 2012.
59. SlimSuite WebApollo search tool. http://www.slimsuite.unsw.edu.au/servers/apollo.php.
60. Edwards RJ, Enosi Tuipulotu D, Amos TG, O'Meally D, Richardson MF, Russell TL, Vallinoto M, Carneiro M, Ferrand N, Wilkins MR, Sequeira F, Rollins LA, Holmes EC, Shine R, White PA: Supporting data for "Draft genome assembly of the invasive cane toad, Rhinella marina" GigaScience Database. 2018. http://dx.doi.org/10.5524/100483

Tables

Table 1. Summary statistics of generated whole genome shotgun sequencing data. Bold rows indicate data used for assembly.

Platform	Library Type	Mean insert size (kb)	Mean read length (bp)	Number of reads	Number of bases (Gb)
HiSeqX (raw)	Paired-end	0.35	147.7	1,857,762,090	282.92
**HiSeqX (filtered)**			**140.6**	**1,205,616,705**	**169.47**
PacBio RS II	SMRTbell	15-50	8,852	2,794,391	24.736
PacBio RS II	SMRTbell	15-50	9,085	595,447	5.409
PacBio RS II	SMRTbell	15-50	10,432	1,867,543	19.482
PacBio RS II	SMRTbell	20-50	10,834	2,487,852	26.952
**PacBio Total**			**9,887**	**7,745,233**	**76.58**
**PacBio Unique[1]**			**10,987**	**6,167,714**	**67.77**

1. Longest read per sequenced molecule (SMRT ZMW).

Table 2. Summary of genome assemblies. For comparison, statistics are provided for two existing neobatrachian genomes, *Nanorana parkeri* (v2) [15] and *Lithobates catesbeianus* (v2.1)[14], and two anuran reference genomes, *Xenopus tropicalis* (v9.1) [16] and *Xenopus laevis* (v9.2) [17]. Lengths are given to 3 s.f. All percentages are given to 1 d.p.

**Genome Assembly**	**Hybrid (v2.2)**	**Short read**	**Long read**	***N. parkeri* (v2.0)**	***L. catesbeianus* (v2.1)**	***X. tropicalis* (v9.1)**	***X. laevis* (v9.2)**
Total Length (Gb)	2.55	3.75	2.69	2.07	6.25	1.44	2.72
No. scaffolds	31,392	19.9 M*	31,392*	135,808	1.54 M	6,822	108,033
Proportion gap (%N)	0.0%	0.1%	0.0%	3.9%	11.6%	4.9%	11.4%
N50	168 kb	583 bp	167 kb	1.06 Mb	39.4 kb	135 Mb	137 Mb
L50	3,373	715 k	3,531	555	31,248	5	9
Longest scaffold	3.53 Mb	72.6 kb	3.64 Mb	8.61 Mb	1.38 Mb	195 Mb	220 Mb
GC	43.2%	43.3%	42.9%	42.6%	43.1%	40.1%	39.0%
**BUSCO[1]**							
Complete Single copy	80.9%	15.5%	2.2%	83.4%	42.3%	87.5%	52.9%
Complete Duplicate	2.2%	0.7%	0.0%	1.6%	0.9%	1.0%	39.8%
Fragment	7.5%	33.6%	2.2%	7.2%	22.3%	6.0%	3.2%

1. BUSCO v2.0.1 short summary statistics (n=3950).

* Statistics for short and long read assemblies refer to contigs used for hybrid assembly.

Table 3. GenomeScope genome size estimates for *Rhinella marina* based on raw trimmed Illumina data using different combinations of k and maximum k-mer coverage. Lengths are in megabases (0 d.p.).

Data	Max kmer coverage	Unique Length (Mb)		Repeat Length (Mb)		Genome Size (Mb)	
		Min	Max	Min	Max	Min	Max
Raw (k=21)	1000	1,365	1,366	489	489	1,853	1,855
Raw (k=21)	10000	1,365	1,365	874	874	2,239	2,240
Raw (k=23)	1000	1,453	1,455	470	471	1,924	1,926
Raw (k=23)	10000	1,454	1,454	842	842	2,296	2,296
Q30 (k=21)	1000	1,307	1,308	462	462	1,768	1,771
Q30 (k=21)	10000	1,307	1,308	749	749	2,056	2,057
Q30 (k=23)	1000	1,389	1,391	438	439	1,828	1,830
Q30 (k=23)	10000	1,390	1,391	713	713	2,103	2,104

Table 4. Estimation of *Rhinella marina* genome size using various methods and the corresponding level of sequencing coverage (3 s.f.). GenomeScope values in this table are mean values from the four setting combinations.

Method	Estimated Genome Size (Gb)	Illumina coverage (X)	PacBio coverage (X)	Reference
Flow cytometry (mean)	4.33	65.3	17.7	[26, 33, 35, 38]
Flow cytometry (min)	3.98	71.1	19.2	[38]
Flow cytometry (max)	4.90	57.7	15.6	[35]
Densitometry (mean)	4.95	57.1	15.5	[32, 34, 36, 37]
Densitometry (min)	4.06[#]	69.7	18.9	[37]
Densitometry (max)	5.65	50.1	13.6	[32]
GenomeScope (raw)	2.08	136	36.8	-
GenomeScope (Q30)	1.94	146	39.4	-
qPCR (zfp292)	2.38	119	32.1	-
Assembly (v2.2)	2.55	111	30.0	-

value adjusted to account for updated size of reference genome used to infer *R. marina* genome size.

Table 5. Summary statistics of consensus protein-coding gene predictions and predicted repeat elements (including RNA genes) for the *Rhinella marina* v2.2 draft genome. Lengths are given to 3 s.f. Coverage and mean depth statistics for PacBio and Q30-trimmed Illumina reads are given to 2 d.p.

**Element**	**Count**	**No. scaffolds**	**Avg. length**	**Total length**	**Genome coverage**	**PacBio depth (X)**	**Illumina depth (X)**
Protein-coding gene	58,302	19,530	18.8 kb	1.10 Gb	42.91%	20.32	58.07
Transcript	58,302	19,530	1.24 kb	72.3 Mb	2.83%	20.49	65.41
- Similar to known	25,846	11,918	1.90 kb	49.1 Mb	1.92%	20.08	56.42
- Unknown	32,456	15,213	714 bp	23.2 Mb	0.91%	20.98	68.82
Exon	309,718	19,530	233 bp	72.3 Mb	2.83%	20.49	65.41
- Coding	294,535	19,530	207 bp	60.8 Mb	2.38%	20.67	66.97
Intron	251,416	18,509	4.08 kb	1.03 Gb	40.09%	20.30	57.55
5’ UTR	15,855	8,839	208 bp	3.29 Mb	0.13%	18.69	53.86
CDS	58,302	19,530	1.04 kb	60.8 Mb	2.38%	20.67	66.97
3’ UTR	11,965	5,780	682 bp	8.16 Mb	0.32%	19.91	58.52
BUSCO SC Complete	3,194	2,014	32.6 kb	104 Mb	4.07%	19.89	53.01
**Repeats**							
SINE	21,620	9,322	338 bp	7.31 Mb	0.29%	19.45	58.23
LINE	268,569	27,620	513 bp	138 Mb	5.38%	21.03	72.29
LTR	201,817	24,949	504 bp	102 Mb	3.98%	22.62	68.96
DNA	817,405	30,689	600 bp	490 Mb	19.17%	21.67	68.37
Helitron	20,319	9,340	826 bp	16.8 Mb	0.66%	19.32	56.81
Retroposon	1,042	829	549 bp	570 kb	0.02%	18.22	50.87
Other	18	17	209 bp	3.7 kb	0.00%	14.27	24.60
Unknown	1,610,883	30,966	513 bp	826 Mb	32.28%	20.12	59.39
Satellite	25,557	10,270	440 bp	11.3 Mb	0.44%	18.38	54.21
Simple repeats	968,947	30,620	56.9 bp	55.1 Mb	2.16%	18.88	48.51
Low complexity	141,028	24,020	51.8 bp	7.30 Mb	0.29%	22.48	64.48
rRNA	5,227	2,923	422 bp	2.20 Mb	0.09%	40.88	142.42
tRNA	5,558	4,474	105 bp	583 kb	0.02%	29.15	140.06
snRNA	21,788	9,432	546 bp	11.9 Mb	0.47%	24.63	89.12

srpRNA	17	11	268 bp	4.55 kb	0.00%	22.11	140.44
scRNA	3	3	69.0 bp	207 bp	0.00%	15.53	47.29
RNA	418	266	482 bp	202 kb	0.01%	32.65	173.99
**Repeat TOTAL**[1]	4,110,222	31,179	406 bp	1.63 Gb	63.9%	20.82	63.79

1. Values for repeat totals account for overlapping repeats.

Table 6. Proportions of predicted protein and transcript sequences exceeding 50%, 80%, 95% or 99% coverage in the top BLAST+ hit from the published transcriptome [18], and combined coverage for the top ten transcript hits. All percentages given to 3 s.f.

**Type**	**Count**	**Coverage in top transcript hit**				**Coverage in top 10 transcript hits**			
		**50%+**	**80%+**	**95%+**	**99%+**	**50%+**	**80%+**	**95%+**	**99%+**
Protein (similar to known)	25,846	93.6	76.7	56.5	40.7	97.5	90.3	72.7	54.2
Transcript (similar to known)	25,846	75.0	50.0	30.8	21.4	82.6	73.1	57.2	40.9
Protein (unknown)	32,456	79.9	49.8	27.1	15.8	85.7	66.3	44.4	29.9
Transcript (unknown)	32,456	43.6	21.5	12.1	8.61	52.6	37.3	25.4	19.1

Figure legends

Figure 1. *Rhinella marina.* An adult cane toad.

Figure 2. Schematic overview of project workflow. A summary of the experimental methods used for sequencing, assembly, annotation and size estimation of the cane toad genome. Transcriptome data (orange segment) was obtained from our previous study [18].

Figure 3. Assessment of genome assembly completeness. BUSCO analysis of *Rhinella marina* genome assembly (v2.0 uncorrected, v2.1 pilon polishing, v2.2 pilon and arrow polishing, combined v2.1, 2.2 and 2.2 ratings), *Lithobates catesbeianus* (v2.1), *Nanorana parkeri* (v2.0), *Xenopus tropicalis* (v9.1) and *Xenopus leavis* (v9.2) genomes using the tetrapoda_odb9 orthologue set (n=3950). The *Xenopus leavis* genome duplication is made clear by the large number of paralogs (light blue) with respect to other assemblies.

Figure 4. GenomeScope k-mer frequency and log-transformed k-mer coverage profiles. (A) raw Illumina data (k=23), (B) Q30 trimmed Illumina data (k=23). Profiles for k=21 are similar (data not shown).

Figure 5. Key protein statistics for predicted genes with and without annotated similarity to known genes. Histograms of (A) protein length, and (B) MAKER2 Annotation Edit Distance (AED), for "similar" (blue) and "unknown" (red) classes of predicted genes.

Figure 6. Multi-tissue gene expression for predicted genes with and without annotated similarity to known genes. (A) Histograms of RNA-Seq TPM for "similar" (blue) and "unknown" (red) classes of predicted genes, capped at 100 TPM. (B) "similar" and (C) "unknown" gene expression, rated as: Very low (<1 TPM), Low (1-9 TPM), Medium (10-99 TPM) or High (100+ TPM).

Figure 7. Assessment of assembly annotation completeness. BUSCO analysis for (A) all BUSCO tetrapoda genes (n=3950), and (B) the subset of BUSCO genes rated as "Missing" from the Richardson *et al.* transcriptome [18]. *R. marina* (combined): combined v2.0, v2.1 and v2.2 ratings; Annotation:

combined MAKER proteome and transcriptome ratings; GigaDB: combined assembly and annotation ratings; Cane Toad: combined assembly, annotation and Richardson *et al.* transcriptome [18].

Figure 8. Phylogenetic supertree of 15 selected chordate taxa constructed from phylogenetic trees for 6,417 high confidence cane toad proteins. Branch labels indicate percentage consistency (see text), rounded down. Numbers following each taxon are the number and percentage of source trees containing that taxon. The tree has been rooted using fish as an outgroup and visualised with FigTree [58]. The full supertree of 52 taxa is available as Figure S1.

Figure 9. Summary of the main annotation classes for *Rhinella marina* genome assembly. Identified repeat classes exceeding 2% of assembly have been plotted separately (1 d.p.). All other repeats, including “Unknown”, have been grouped as “Other repeats”. The percentage for introns excludes any repeat sequences within those introns.

Figure 1

Click here to access/download;Figure;Figure 1.eps

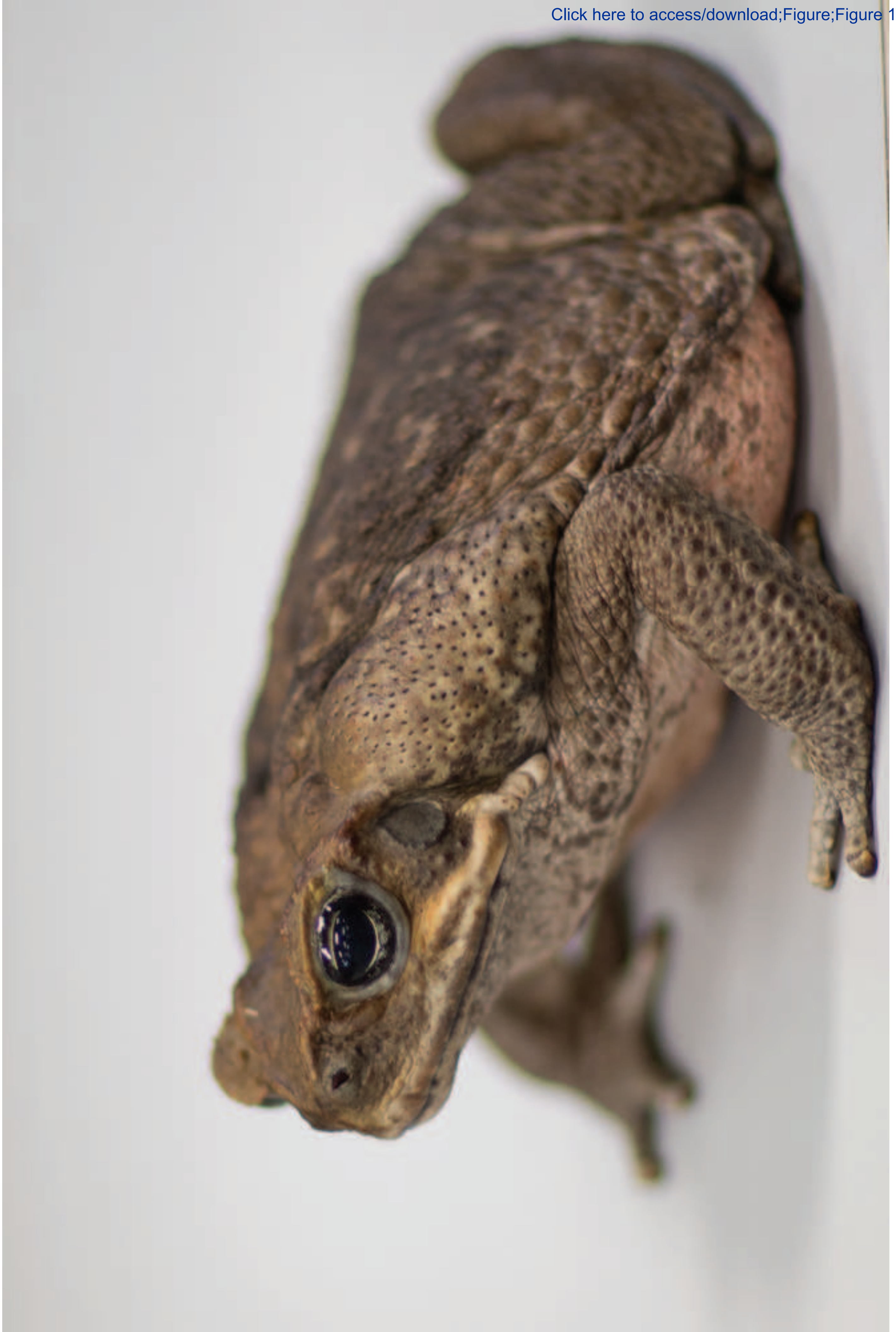

Figure 2

Click here to access/download;Figure;Figure 2.eps

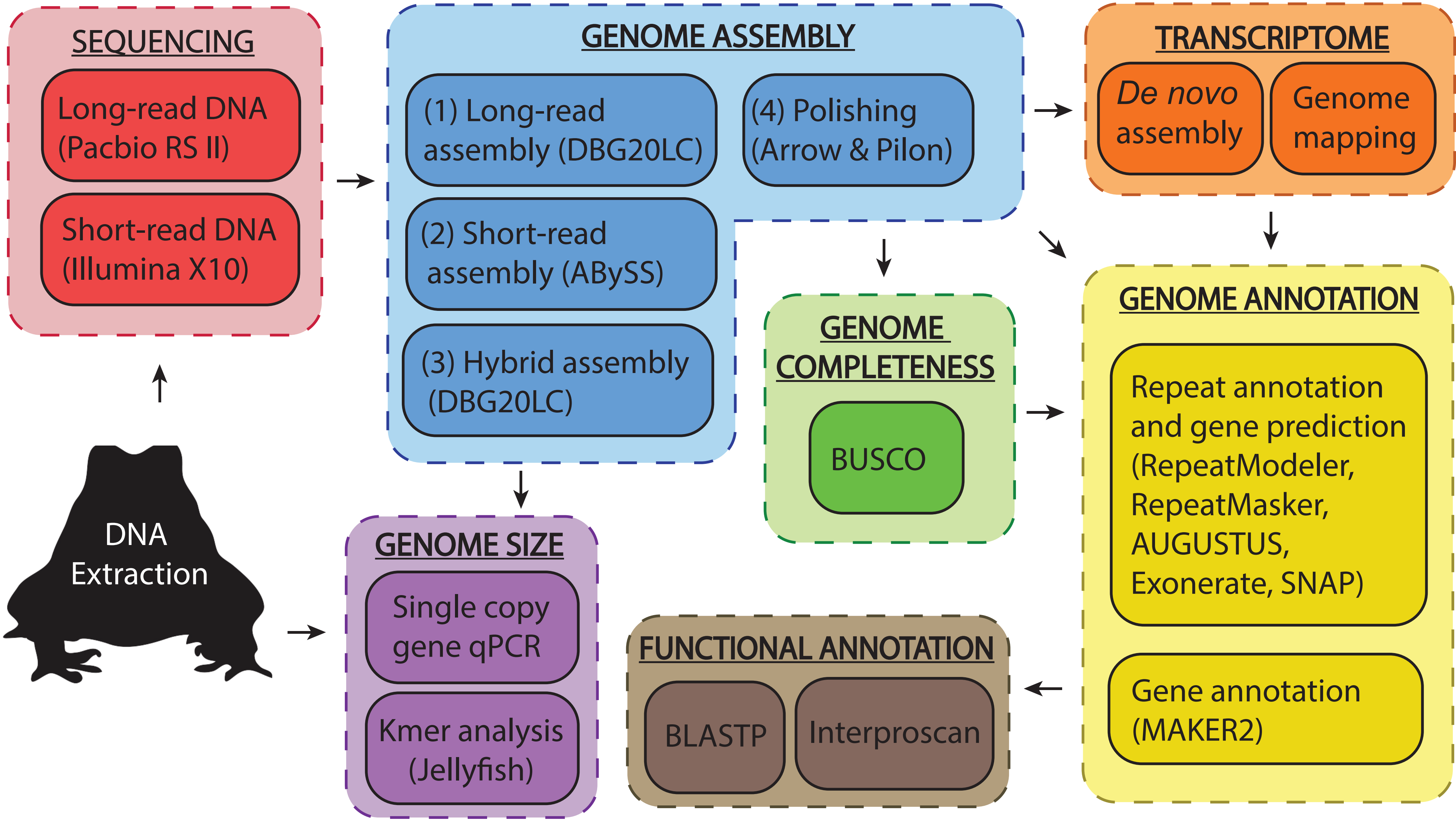

Figure 3

Click here to access/download;Figure;Figure 3.eps

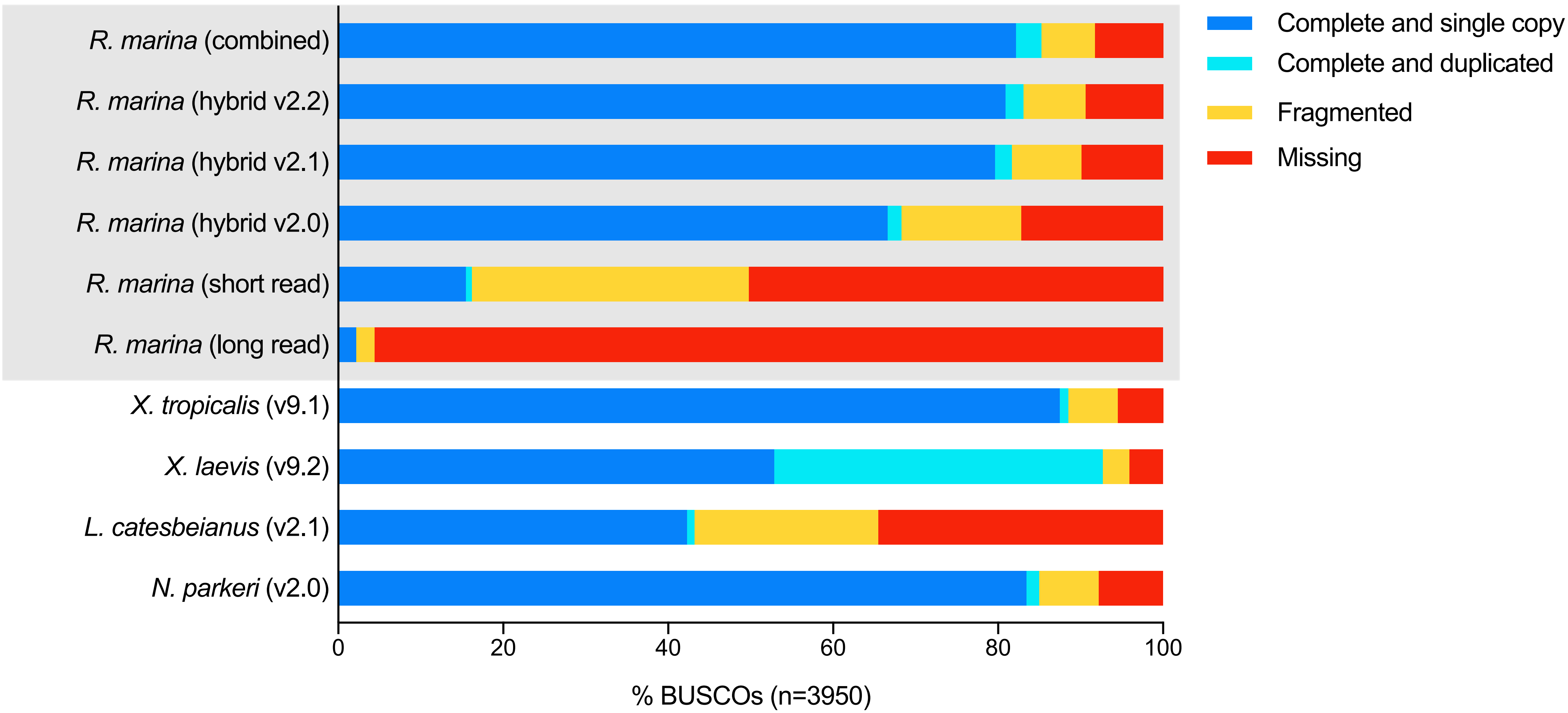

Figure 4

Click here to access/download;Figure;Figure 4.eps

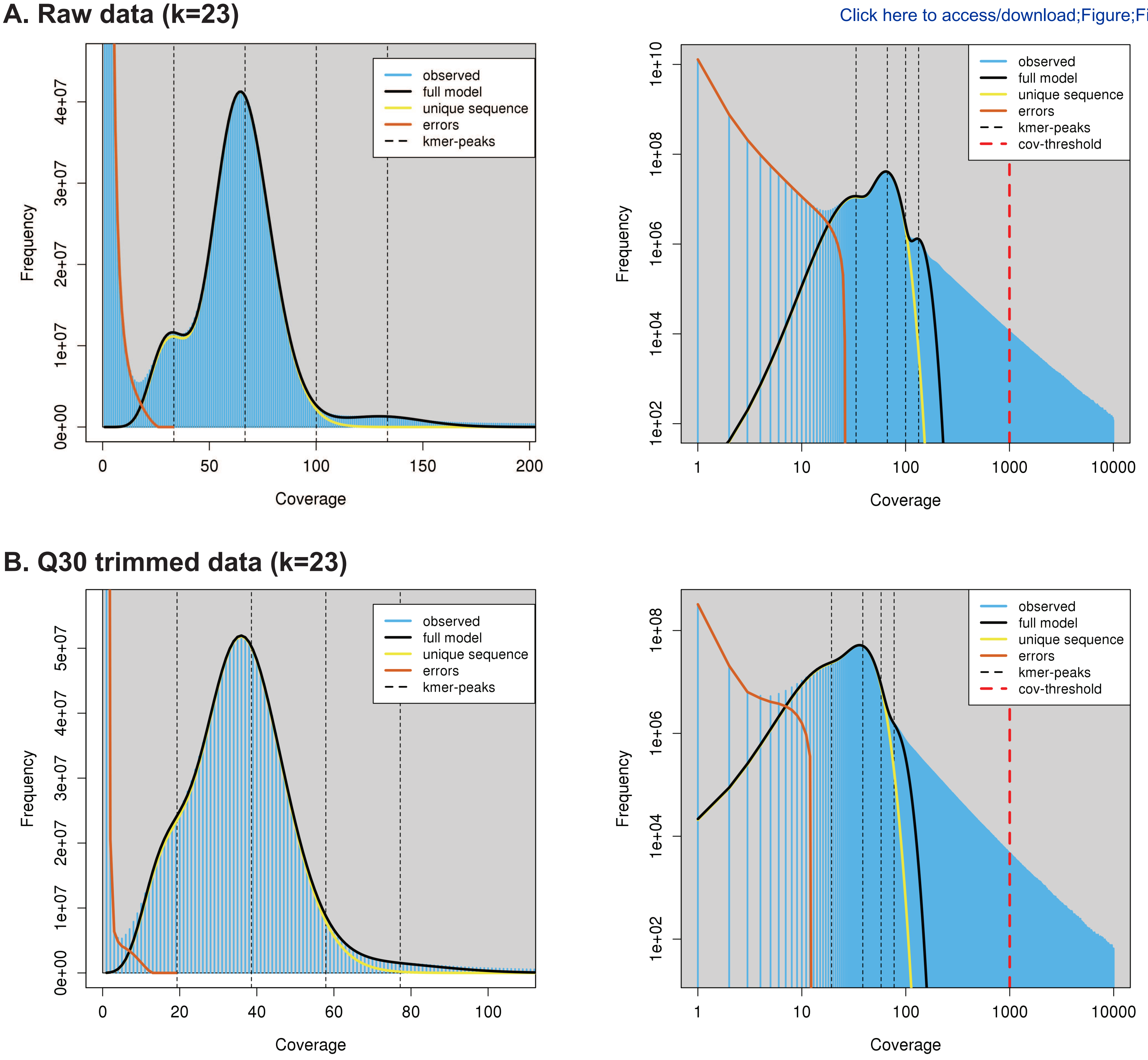

Figure 5

Click here to access/download;Figure;Figure 5.pdf

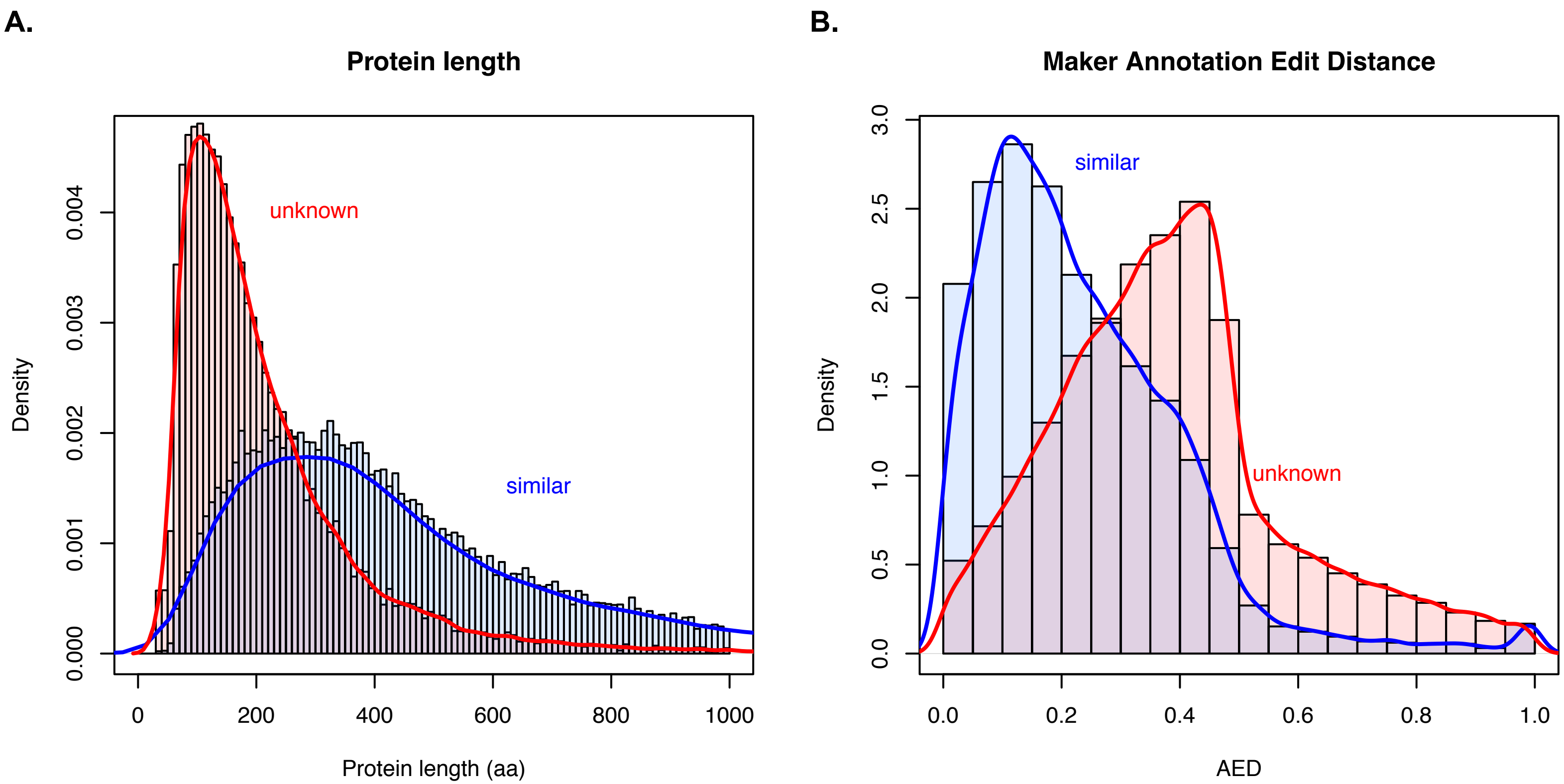

Figure 6 Click here to access/download;Figure;Figure 6.pdf

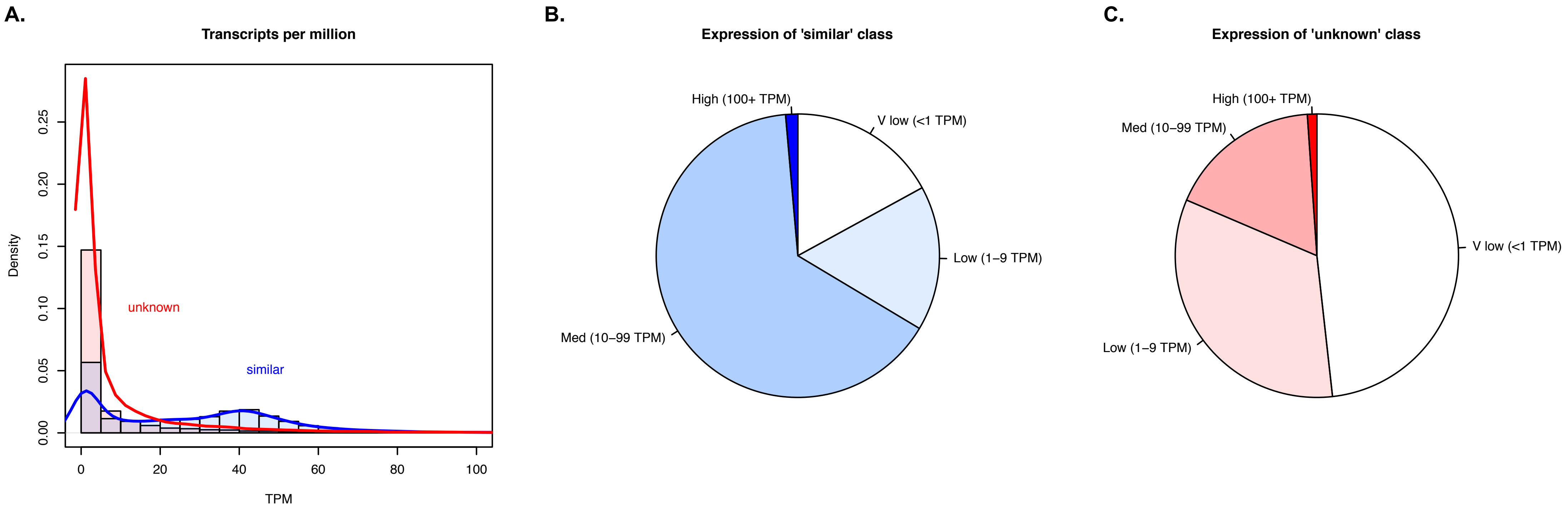

Figure 7

Click here to access/download;Figure;Figure 7.eps

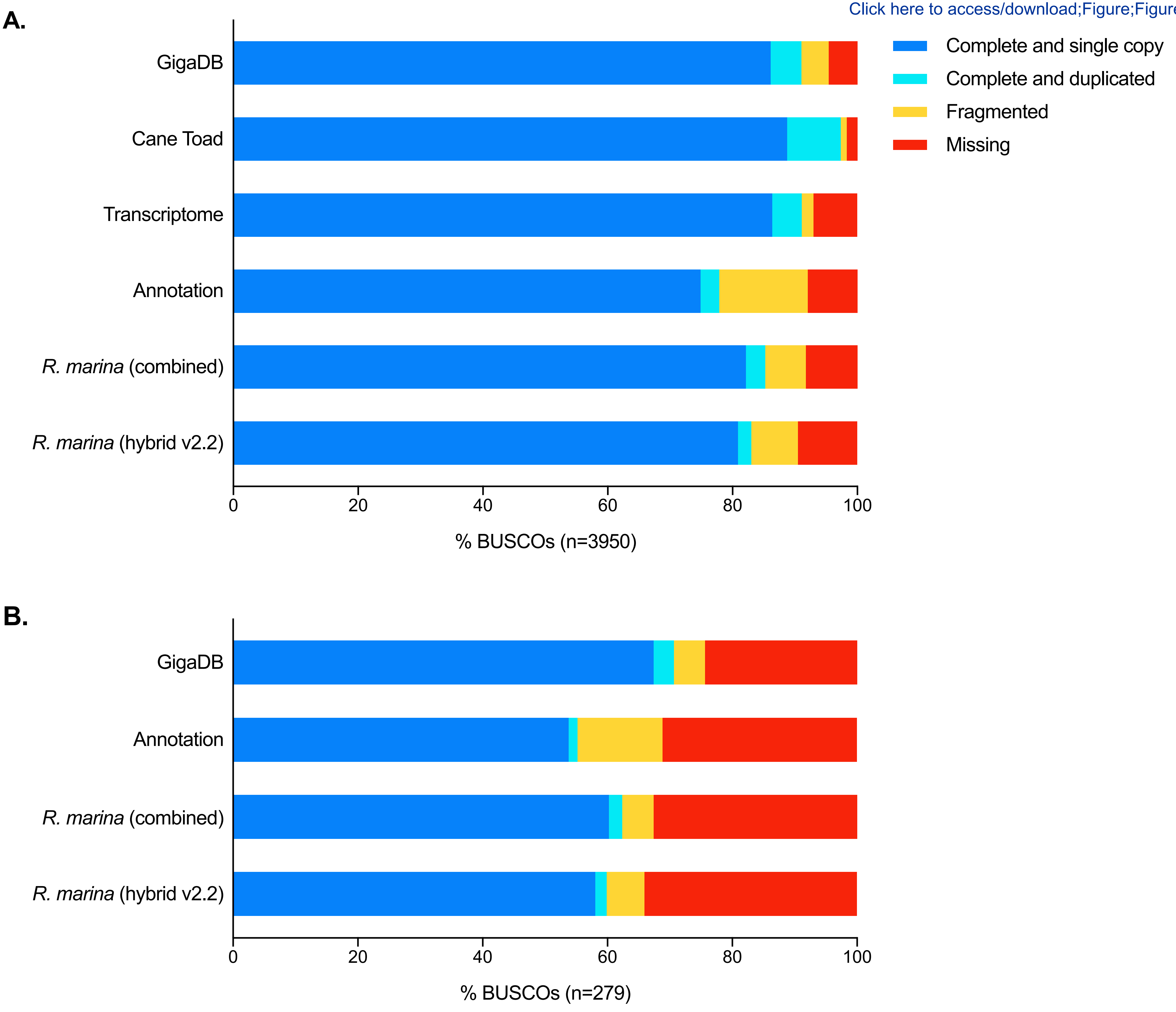

Figure 8

Click here to access/download;Figure;Figure 8.pdf

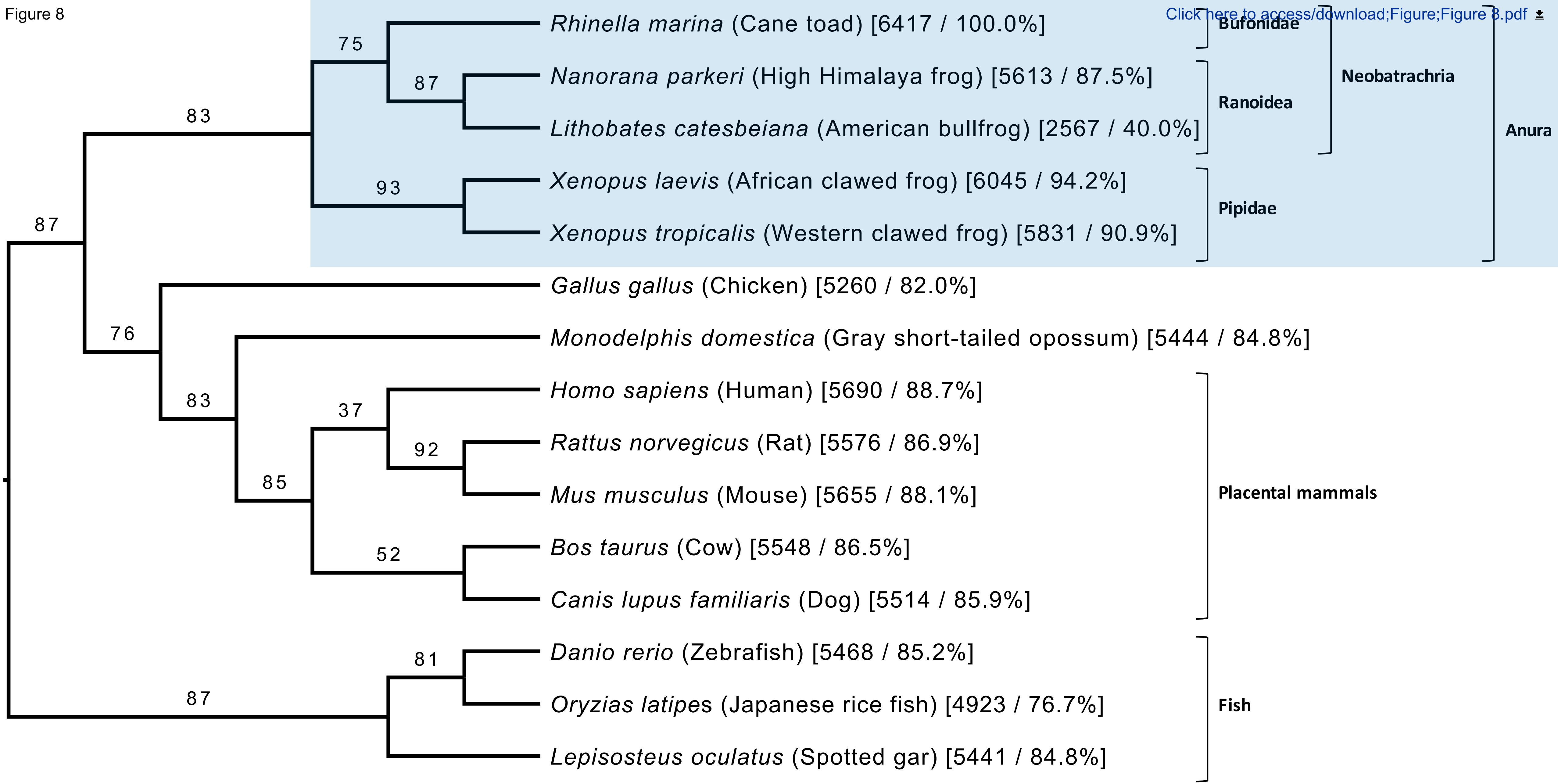

Click here to access/download;Figure;Figure 9.eps

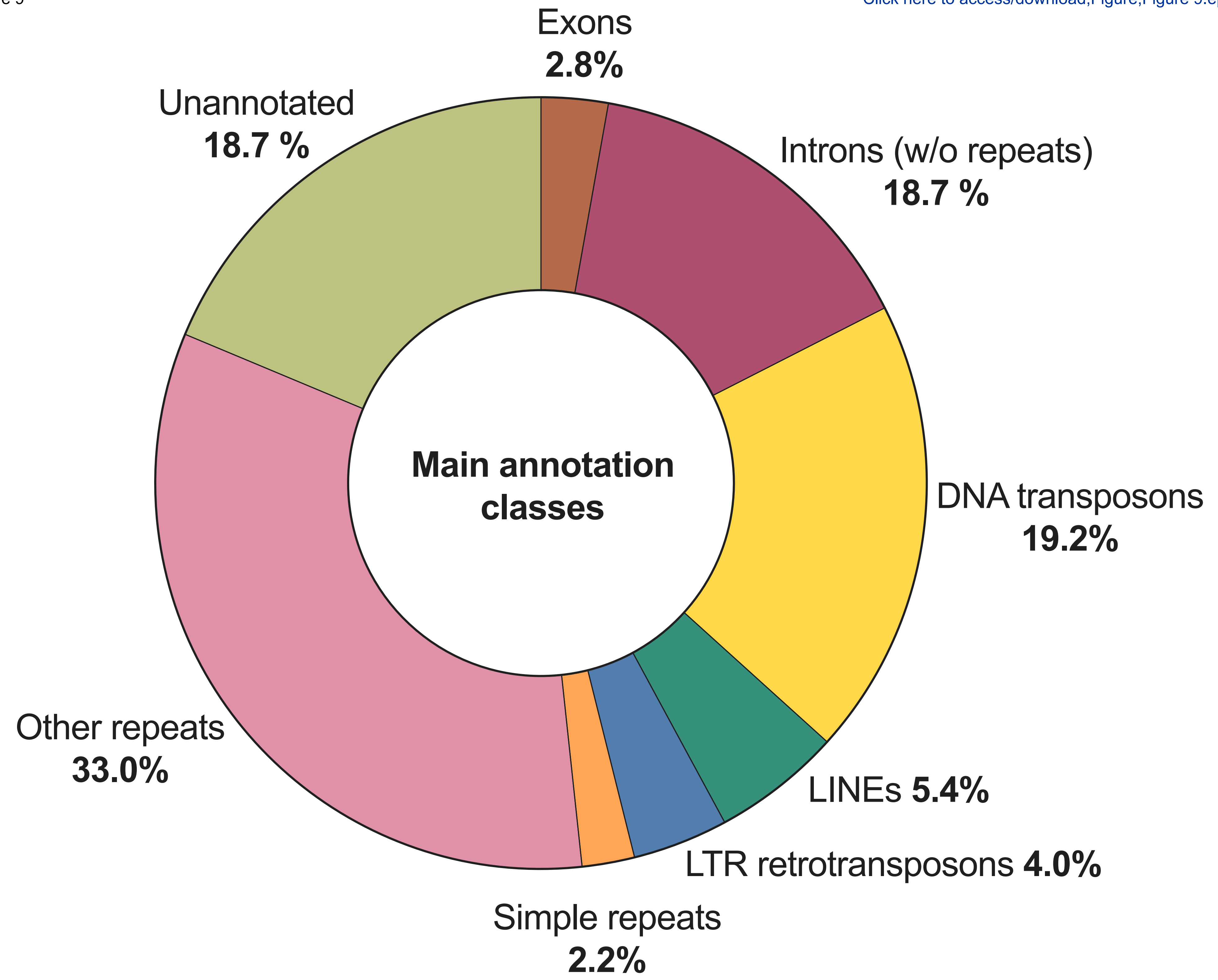

Figure S1

Click here to access/download
Supplementary Material
Figure S1.pdf

Table S1

Click here to access/download
Supplementary Material
Table S1.xlsx

Table S2

Click here to access/download
Supplementary Material
Table S2.xlsx

Table S3

Click here to access/download
Supplementary Material
Table S3.xlsx

Table S4

Click here to access/download
Supplementary Material
Table S4.xlsx
