# Supplementary material for: Draft genome assembly of the invasive cane toad, Rhinella marina
Source: Gigascience. 2018 Aug 7;7(9):giy095. doi: 10.1093/gigascience/giy095 (PMC6145236; doi:10.1093/gigascience/giy095)
Supplement: Additional Files [file giy095_supplemental_files.zip › Figure S1.pdf]

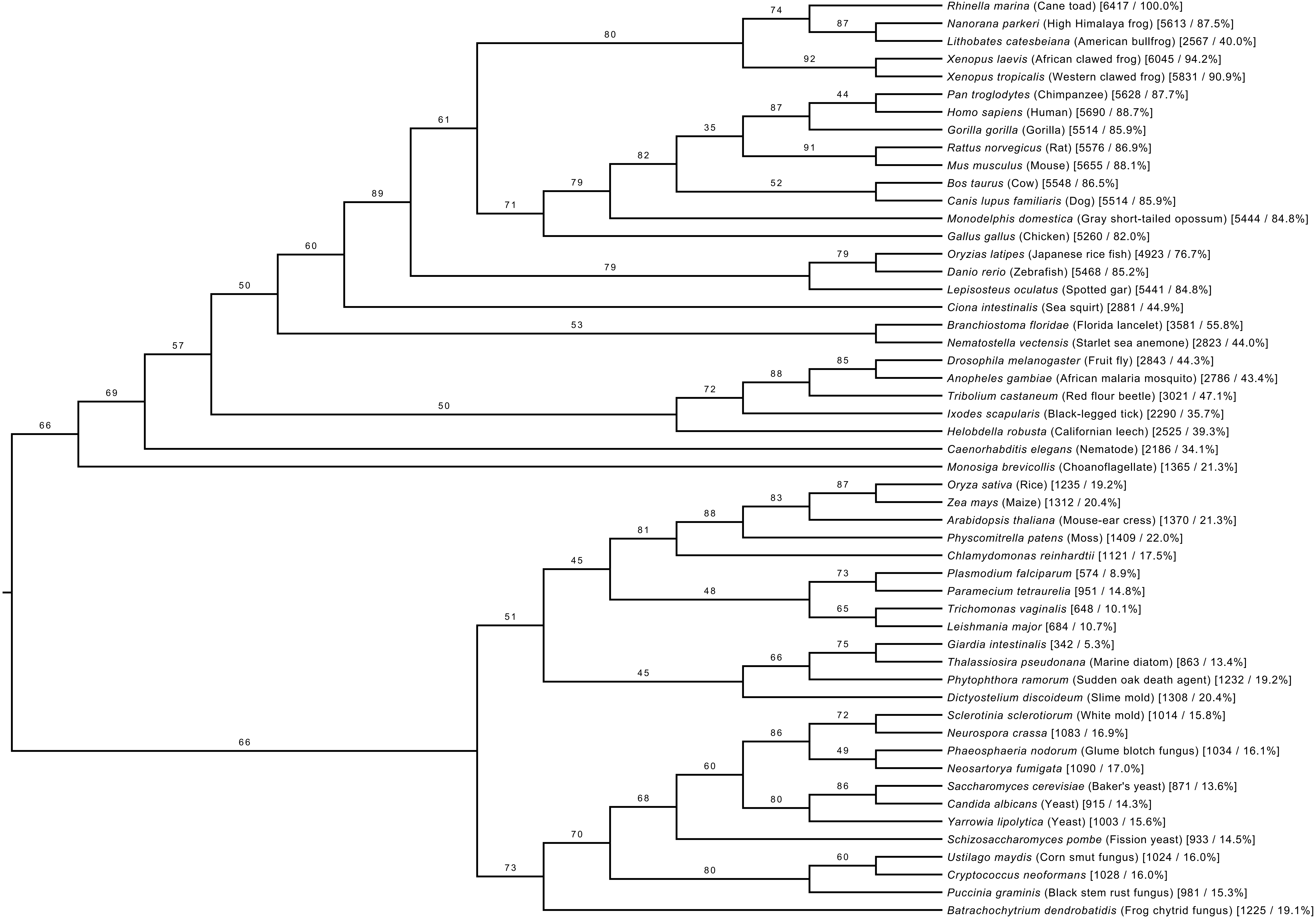

Figure 8. Phylogenetic supertree of 15 selected chordate taxa constructed from phylogenetic trees for 6,417 high confidence cane toad proteins. Branch labels indicate percentage consistency (see text), rounded down. Numbers following each taxon are the number and percentage of source trees containing that taxon. The tree has been rooted using fish as an outgroup and visualised with FigTree [58]. The full supertree of 52 taxa is available as Figure S1.
