# Supplementary material for: Draft genome assembly of the invasive cane toad, Rhinella marina
Source: Gigascience. 2018 Aug 7;7(9):giy095. doi: 10.1093/gigascience/giy095 (PMC6145236; doi:10.1093/gigascience/giy095)
Supplement: Response_to_Reviewer_Comments_Original_Submission.pdf [file giy095_response_to_reviewer_comments_original_submission.pdf]

Author's Response To Reviewer Comments

Close

Please note that text within quotation marks is new or amended text taken directly from the revised manuscript.

Reviewer #1

1. Where I think the authors fall short is on not reporting any insights about (or derived from) the genome (aside from repeat content), despite having the first-hand look at it. It is a data note and therefore no requirements for biological analyses, but surely something can be said about the genes you have predicted? E.g. any gene families stand out? Are there genes in your genome draft that could explain, at least partially, the enormous success this species has in non-native environments? For instance, what is known about the gene(s) involved in the production of the toxic secretions you mentioned? Are there any clues from the resources you are sharing with the community?

We have had an incredible amount of interest from researchers with requests for access to this genome assembly for further biological studies. Thus, the main driving factor for submission was to make our data publicly available for more detailed analysis by the scientific community. Given that this is a data note, we believe that comprehensive biological analyses are best suited for follow-up publications. Further analysis will delay publication and prevent sharing of this highly petitioned dataset. However, we have released the genome in a WebApollo genome browser and included additional analysis of the predicted proteins, which we hope will encourage community annotation and curation of the genome which will aid such biological analyses (lines 286-291):

"Future work will be needed to improve the quality of gene annotation. We have included all of the MAKER2 predictions in our annotation and a full table of protein statistics and top blastp hits from this analysis for further biological analyses (Table S3). Annotation has also been made available via a WebApollo [53] genome browser (http://edwapollo.babs.unsw.edu.au/) and an associated search tool (http://www.slimsuite.unsw.edu.au/servers/apollo.php). This will facilitate community curation and annotation of genes of interest."

Furthermore, it should be noted that the authors have several publications currently under review that address the questions put forward by the reviewer regarding genes involved in invasion and toxic production.

2. I look at the supporting data available on the FTP server and everything checks out. I do have a recommendation for an additional file (see below).

We have added a high-confidence gene set as recommended (see point 4, below).

3. The authors claim that the draft genome "sets a milestone in the field of anuran genetics".

I would like the authors to describe why it is so, in their conclusion.

Both reviewer 1 and 3 have made a comment regarding the use of the word “milestone”. This has been omitted from the sentence.

4. Typically, for genome papers, a high-confidence gene set is also reported/provided (in addition to what is presented, often based on AED and other criteria). A high confidence set would be a very useful resource to have, reduce the gene space in the process and present a more focused, gold standard list of better-annotated genes. This set stands a higher chance of yielding valuable and meaningful insights for your and future studies, set that would hold against scientific scrutiny (In the process weeding out the many, potentially spurious, gene predictions reported herein).

We have created a high confidence gene set as recommended by the reviewer. This is based on MAKER2 Annotation Edit Distance (AED) and reciprocal high coverage BLAST hits to reference proteomes. We have also generated a table (Supplementary Table S4) with additional supporting data for each predicted gene to make it easier for users to identify subsets that meet confidence criteria appropriate to their own goals. The high confidence gene set has been uploaded to GigaDB (lines 291-294):

"For researchers who would like to use cane toad proteins in general evolutionary analyses, we have also created a "high quality" dataset of 6,580 protein-coding genes with an AED no greater than 0.25 and at least 90% reciprocal coverage of its top QFO blastp hit, excluding possible viral and transposon proteins, available from the GigaScience database."

5. The sentence on line 240 starting with "Critically.." is not accurate and needs to be re-worked. FYI some short read assemblers are able to assemble through repeats larger than read length with the help of paired-end information. You could rephrase to something like "The average length (XX +/- Std. dev.) of most (XX%) of these repeat classes exceeds that of the Illumina reads used in our study (Paired-end 150bp), making the short read assembly difficult in these regions. This is reflected by the low assembly contiguity (contig N50 length = 583bp)."

We agree with the reviewer that this sentence over-states the problems presented to short read assembly and have rephrased that sentence:

"The mean repeat length is 406 bp, which exceeds the Illumina read length used in our study (mean 140.6 bp paired-end). This makes short-read assembly of these regions difficult, as reflected by the poor ABySS contiguity (contig N50 = 583 bp, Table 2), and emphasises the need for long read data in this organism."

6. Though I must say that such a low contiguity figure is very untypical for an ABySS assembly, even for a highly repeated genome. Especially since your library captures sizes as long as 800bp. I am concerned about gDNA content/representation, as you seem to only

have constructed a single paired-end library. Building multiple libraries from the same tissue source, preferably from 2 or more samples, prevent possible sampling/lab manipulation biases and ensures you have captured the entire genomic content. I also recommend building libraries of various insert sizes: 500, 2kbp, 5kbp whenever possible, especially when it is your only source of long-range information for assembly. This helps short read assemblers resolve repeats and increase the contiguity of the resulting assembly. Since you mainly used the ABySS short-read assembly for improving the accuracy of the DBG2OLC long read one, it might be ok in this case (especially since you recover many complete BUSCOs), but it also explains why a hybrid assembly approach does not improve the N50 length metric of the long-read DBG2OLC assembly - where I think it should.

We agree with the reviewer that the ABySS assembly is not as good as one might expect given its performance in other species. This was reflected by comparatively poor performance by other short read assembly attempts. We have had much better success using the same library preparation, PE strategy and ABySS assembly in other species. We think that the difficulties we've experienced whilst attempting to assemble the genome from short read data is most probably related to its high repeat content (see point 5, above). We acknowledge that it could also be influenced by gDNA representation, although the high BUSCO coverage gives us confidence of good coverage.

We agree that multiple insert sizes have improved the short-read assembly. However, we decided that generating more long read data was more useful. The reviewer is correct that we "mainly used the ABySS short-read assembly for improving the accuracy of the DBG2OLC long read one". We acknowledge that this is not a final, complete cane toad genome, and trust that future sequencing efforts will be able to improve upon our assembly. Despite this, the draft genome in its current state will be enormously useful to the community.

7. The cane toad reference transcriptome was published by the Authors and used as direct evidence for MAKER gene prediction. The Authors briefly mentioned it as a "multi-tissue" from tadpoles and adults. It would be good to provide more information (2-3 sentences) on this evidence in the present study (so readers readily know what went in the gene prediction tools), especially if that information could be used to gain insights on cane toad genetics.

The following sentence has been added to the MS (lines 211-213).

"Whole-tadpoles and the brain, liver, spleen, muscle, ovary and testes of adult toads from Australia and Brazil were used to prepare cDNA libraries for the multi-tissue transcriptome sequencing."

8. line 219, typo, should read "Approximately"

This has been fixed in the manuscript.

9. Make sure you report to single digit (or double) consistently, throughout.

Table 2 has been fixed to give all percentage values to 1 d.p. Elsewhere, we have tried to consistently use the number of significant figures or decimal places that we consider to be appropriate for given values.

Reviewer #2

1. Although BUCSO analysis can be used for genome completeness, it is based on protein coding genes, so I think 'Assessment of genome completeness' would be better to be merged with 'Genome annotation and gene prediction' section.

We respectfully disagree with the reviewer on this point. BUSCO is a set of software and data for "assessing genome assembly and annotation completeness with single-copy orthologs". An explicit objective of the tool is assessing genome completeness. It is also an important part of our manuscript's narrative that our draft genome is capturing the majority of protein-coding regions well, despite being quite fragmented when contig statistics alone are considered. However, we acknowledge that BUSCO can also be used to assess annotation completeness and have added the BUSCO short score for the MAKER2 gene set to the 'Genome annotation and gene prediction' section, along with more discussion of observed differences (see point 2, below).

2. In previous publication with R. marina transcriptome (Richardson, et al., GigaScience, 2018; doi: 10.1093/gigascience/gix114; Ref #18 on current manuscript), it was reported that 1.7% of BUCSO genes were fragmented, and 7.4% of them were missing on their 62,202 CDS transcripts. These numbers look better than genome-based result described in this manuscript (7.5% of fragmented, and 9.5% of missing). Authors may need to discuss the difference among these two annotations.

These differences are consistent with results from the BUSCO manuscript, in which it states: "Nevertheless, the fact that some genome assemblies appear less complete than their corresponding gene sets (e.g. H. sapiens Table 1) reveals limitations of the BUSCO gene prediction step." … "Thus, it should be noted that while BUSCO assessments aim to robustly estimate completeness of the datasets, technical limitations (particularly gene prediction) may inflate proportions of 'fragmented' and 'missing' BUSCOs, especially for large genomes." Deeper analysis of our BUSCO results to confirm this have now been included in the discussion of BUSCO results.

Lines 153-158: "It should be noted that these numbers mask some underlying complexity of BUSCO assessments; aggregate improvements in BUSCO scores with polishing include some losses as well as gains. Taking the best rating for each BUSCO in v2.0, v2.1 or v2.2 reduces the number of missing BUSCO genes to 326 (8.3%) and increases the complete number to 3366 (85.2%) (Figure 3, "R. marina (combined)"). This is explored further in the "Genome annotation and prediction" section, below."

Lines 270-285: "We ran BUSCO v2.0.1 (short mode, lineage tetrapoda_odb9, BLAST+

v2.2.31 [28], HMMer v3.1b2 [29], AUGUSTUS v3.2.2 [30], EMBOSS v6.5.7 [31]) on the MAKER2 transcriptome and proteome and retained the most complete rating for each gene (Figure 7A, Table S2, "Annotation"). MAKER annotation had fewer missing BUSCO genes than the v2.2 assembly (314 vs 375) but many more fragmented (561 vs 296). Equivalent BUSCO analysis of the Richardson et al. transcriptome [18] was only missing 296 genes. However, as seen with the assembly versions, these values mask hidden complexity. Combined BUSCO analysis of our hybrid assembly (v2.0, v2.1, v2.2) and annotation, revealed only 181 missing genes (Figure 7A, Table S2, “GigaDB”). Furthermore, >50% of the 279 genes “Missing” in the transcriptome are found in the genome and/or its annotation (Figure 7B, Table S2). When the transcriptome and our genome are combined, only 68 BUSCO genes (1.7%) are “Missing” and 3845 (97.3%) are “Complete” (Figure 7B, Table S2, “CaneToad”). This highlights the usefulness of our assembly, and illustrates the complementary nature of genome and transcriptome data: the former is more comprehensive but more difficult to assemble and annotate, whereas the latter is easier to assemble into full-length coding sequences but will miss some tissue-specific and lowly expressed genes. Some of the remaining "Missing" BUSCO genes may be present but too fragmented to reach the score threshold."

3. The analysis of 'unknown function' genes with published de novo transcriptome (p.9 line 229-) seems to have a circularity. Authors used all RNA-seq data already on their annotation, which are also used for de novo transcriptome construction (p.9 line 206). So instead of analyzing their matched length, I recommend to analyze their expression level from RNA-seq data. If 'unknown function' genes were mis-annotated genes as authors thought, it should have lower level of evidence for expression, compared to 'known function' genes.

We disagree with the reviewer that there is circularity in our argument. The same RNA-Seq data was used for prediction of both annotated and “unknown function” genes, so there is no reason for any difference in how well different subsets of predicted genes map to the transcriptome. (The transcriptome data was not pre-filtered at any step based on annotation.) Nevertheless, we agree that it is useful to look at expression levels. This has been incorporated into our extended analysis of the predicted genes (lines 247-254):

"We also reanalysed the multi-tissue RNA-Seq data from Richardson et al. [18] by mapping the reads onto the MAKER predicted transcripts. Filtered reads (adaptor sequences and reads with avg. Phred < 30 removed) were mapped with Salmon v0.8.0 [51] (Quasi-mapping default settings, IU libtype parameter). Read counts were converted into transcripts per million (TPM) by normalising by transcript length, dividing by the sum of the length-normalised read counts, and then multiplying by one million. We observed lower expression levels overall in the “unknown” set (Figure 6). With the caveat that real proteins may have very low expression, this is also consistent with the “unknown” gene set containing false annotations."

4. '3 s.f' (significant figure) notation on table headers make the reader confused. It is obvious to recognize by looking at numbers on table, so it would be better to remove it.

Reviewer 3 disagrees with this reviewer and has asked to place the shorthand ‘s.f.’ in the abbreviation list. We have kept ‘s.f.’ in the manuscript.

5. In Table 4, qPCR value is also the average of two experiments (p.8, line 190-191), so it would be fair to present min/max values for that.

These have now been included in the main text (line 193-195):

"Genome sizes were generated from the formulae outlined by [41] and the average of two estimates (2.81 Gb and 1.94 Gb) were used to obtain a genome size of 2.38 Gb."

Reviewer #3

1. p. 11; line 226: The authors identified 32,456 genes with unknown function in addition to the 25,846 predicted genes. The number of these unknown genes seem to much more than expected, but their explanation for it is insufficient. They mentioned that the median length is 171 aa, but what is the cut-off length of amino acids, and what is their range (the minimum and maximum)? In which regions in the genome sequence are those genes located? That is, are those genes scattered in the unique sequence in the genome or localized in the regions with repetitive sequences, transposable elements, or some other specific sequences? If the authors use the same strategy of pipelines for gene annotation with the X. laevis and X. tropicalis genome sequences, how many genes with unknown function could be identified and what percentage of them could be orthologous to those of R. marina?

We have expanded our analysis of the predicted genes, including analysis on the number of genes with of unknown function which have homologues in the Xenopus tropicalis reference proteome. (See also responses to Reviewer 1 (point 4), and Reviewer 2 (points 2 and 3).

Lines 235-244: "Further review of the predicted protein descriptions revealed 4,357 with likely origins in transposable elements (including 4,114 LINE-1 ORFs) and 215 from viruses, however many of these may be bona fide functional members of the cane toad proteome.

Poor quality protein predictions are generally shorter (generated from fragmented or random ORFs) and have a larger Annotation Edit Distance (AED) when compared to real proteins. Consistent with this, the predicted proteins of unknown function are shorter in sequence (median length 171 aa) to those with Swissprot hits (median length 388 aa) (Figure 5A) and have a greater AED (median 0.37 versus 0.2) (Figure 5B)."

Lines 255-269: "To investigate the role of fragmented ORFs, we downloaded the Quest For Orthologues (QFO) reference proteomes (QFO 04/18) [52] and used BLAST+ v2.2.31 [28] blastp (e-value < 10-7) to identify the top hit for each predicted protein in (a) all eukaryote reference proteomes, and (b) the Xenopus tropicalis reference proteome. BLAST results were converted into global coverage with GABLAM v2.28.3 [50]. As expected, the vast majority (99.6%) of “similar” proteins had a blastp hit the QFO proteomes (data not shown).

Perhaps surprisingly, nearly two thirds (66.5%) of “unknown” proteins also had a blastp hit, but these had lower coverage of the reference proteins than did proteins in the “similar” class (data not shown). A “combined coverage” score was calculated for each protein, taking the minimum percentage coverage of either the query protein or its top QFO hit. This metric was related to annotation quality, showing an inverse relationship with AED (data not shown). Excluding proteins with annotation indicating possible viral or transposable element origin, 45.7% of "similar" proteins and 96.8% of “unknown” proteins had the same closest X. tropicalis blastp hit as another predicted protein. Consistent with this being related to gene fragmentation, there was a negative relationship between the number of cane toad proteins sharing a given X. tropicalis top hit, and how much of the X. tropicalis hit was covered by each cane toad protein."

Re-annotation of the Xenopus genomes would be a major undertaking and is beyond the scope of this paper.

2. Figure 5: The authors need to compare the data in Figure 5 with those of other amphibian species.

We agree with the reviewer that such a comparison would be interesting, but disagree that it is necessary. We are currently unable to generate the required data with sufficient rigor to be confident of a fair comparison and this is not the direct focus of the Data Note.

3. Is Rhinella marina the same as Rhinella marinus and Bufo marinus? The authors need to describe this in the abstract and introduction for clarification.

They are the same organism. Bufo marinus is an old scientific descriptor and has been replaced with Rhinella marina. This has been clarified in the abstract (line 52) and the introduction (line 76).

4. The genome size usually means the size of haploid DNA, but, in the text and table, the authors mentioned "a haploid genome size." When the authors simply use "the genome size," does this mean "a haploid genome size?" If so, better not to use "a haploid genome size."

“haploid genome size” has been changed to “genome size” throughout the manuscript.

5. p. 10, line 179: If PCR conditions are nothing special, those could be written in the legend of Tables or Figures, or deposited to "protocol.io."

PCR conditions have been moved to the legend of Table S1.

6. The authors should include s.f. and other abbreviations, if any, that are not listed, in the list of abbreviations.

AED, BLAST, HMM, lncRNA, ORF, QFO, TE, TPM, and s.f. have been added to the abbreviations list.

Reviewer #4

1. The authors take a hybrid assembly approach and mix a single sized 350 bp fragment Illumina library with larger fragment PacBio libraries. They extracted DNA from liver from an adult female. Liver is known to endoreduplicate, which can create rearrangements and problems for de novo assembly projects. However, BUSCO analysis indicates that many of the single genes have been identified in the assembly and their results are comparable to X. tropicalis, arguably the most well assembled and annotated amphibian genome available.

We agree with the reviewer that the BUSCO analysis is sound and that our results compare well with X. tropicalis and we hope to improve our assembly in the near future by sequencing of variety of tissue types. See also response to reviewer 1, point 6.

2. They used ABySS to assemble the genome but given that this genome note format is highly technical, it might be useful to report comparisons with other assemblers that they no doubt tried and/or provide more explanation for using ABySS relative to other assemblers.

We believe such comparisons are more appropriate for a technical note than a data note. The reviewer is correct that multiple assemblies and options were tried. However, we do not feel confident that we can use these data to provide robust technical insight.

3. Regarding their metrics in Table 2. I was confused by the %N reporting for their assembly and long read libraries. The authors report 0.0% of the assembly is in gaps, which is surprising given how repetitive amphibian genomes are, how poorly assembled the toad genome is (though comparably poor to other amphibians which have Ns) and nearly all vertebrate genome assemblies (including the human genome) have some bases unresolved and/or in gaps marked by a series of Ns. The proportion in gaps is an important metric of assembly quality. If the genome really does not have any Ns, it might be useful to highlight this unique attribute somewhere in the text and provide some explanation for how they were able to eliminate gaps.

The hybrid assembly produced is primarily error-corrected long read contigs, not scaffolds. As such, the lack of Ns represents an inability of the hybrid assembler to scaffold the contigs using the ABySS assembly (see reviewer 1, point 6), rather than gap elimination.

4. Their k-mer genome size estimation analysis shows the effect of kmer size and quality trimming but remains far from the estimated genome size based on flow cytometry and other experiments. The authors follow this up with a nice qPCR experiment and provide explanation for how far they are off. Given that the genome assembly size deviates substantially from the reported size, I would worry about using this assembly to analyze repeat content (as the authors state in the manuscript).

We agree with the reviewer's reservations. We report the repeat content of the assembly, not the genome. We explicitly do not claim that this assembly is a final and completely accurate representation of the true genome sequence. We draw attention to the difficulty that the repeats present for accurate assembly.

5. As an additional confirmatory experiment to help build confidence in their results, I wonder if a synteny analysis with Xenopus tropicalis would be useful. Such a comparison might help reveal more about overall synteny and/or continuity and further strengthen their assembly results.

This is a great idea but beyond the scope of this paper.

6. Line 193-199: Here there is discussion about first estimate of genome size using either k-mer or qPCR analysis. This is not the first genome size estimate based on kmer distributions. Perhaps the authors want to state that this is the first amphibian genome estimated in this way? Maybe downplaying this sentence, or more clearly defining what they want to say here would be useful.

This sentence has been modified as per the reviewer's suggestion for better clarity (lines 199-200):

"Given this is the first estimate of the cane toad genome size using either k-mer or qPCR analysis, …"

7. There are a number of sentences in the text that oversell the results a bit and these should be corrected (for example: line 54-55---consider eliminating the line about iconic status and major gaps in understanding cane toad genetics…..this is the case for nearly all organisms; line 248---the fragmented draft assembly, early stage protein-coding annotation results, and estimates that deviate from expectation is contributing to additional fragmented amphibian assemblies; a milestone should go further than what is reported in the manuscript).

Line 54-55: in our opinion this is not over-selling. No results have been presented and only facts are stated.

Line 248 (now line 309): we agree with the reviewer and the sentence has been modified to remove 'milestone'.

8. The authors use MAKER2 for their gene annotation pipeline combined with their reference transcriptome. Given their abundant RNA-Seq, I was surprised that they did not use BRAKER1, which typically provides superior annotations compared to MAKER2. This might explain why it appears they have highly over-predicted the number of genes in the toad genome, though it could also stem from poor assembly. MAKER is widely used but their abundant RNA-Seq data is perfect for using BRAKER1 and they may obtain superior annotations using this tool.

We did consider BRAKER1 during the annotation phase. It is our understanding that later releases of Maker perform just as well as BRAKER1, with the additional benefit of repeat masking and protein alignments which BRAKER does not generate. We hope that making the data freely available, others will be able to improve on the annotations in time. To aid with this endeavour, we have also released the genome in a WebApollo genome browser, as pointed out in the response to point 1 by reviewer 1.

9. In some locations of the text, genus and species are italicized, in other locations they are not. Fix according to journal format requirements.

This has been fixed throughout the manuscript.

Close
