# Supplementary material for: Draft genome assembly of the invasive cane toad, Rhinella marina
Source: Gigascience. 2018 Aug 7;7(9):giy095. doi: 10.1093/gigascience/giy095 (PMC6145236; doi:10.1093/gigascience/giy095)
Supplement: Response_to_Reviewer_Comments_Revision_2.pdf [file giy095_response_to_reviewer_comments_revision_2.pdf]

Author's Response To Reviewer Comments

Close

Please note that quoted text is from the manuscript. Additions or modifications to text within the manuscript are explicitly stated.

Comment 1.

This manuscript adopted "a hybrid de novo whole genome assembly strategy," a relatively new technique, which should require more quality controls than the conventional technique combining shotgun sequences, mate-pair sequences, PacBio long read sequences, and Hi-C or CHICAGO methods. Most comments from the reviewers including mine for basic analyses with the assembly are for quality controls, not for analyses "beyond the scope of this paper," as the authors say. Furthermore, they used a wild-caught frog, which must contain distinct alleles, probably making assembly processes difficult. How did the authors overcome allelic differences? I'm worried about one undesired possibility that the current assembly contains one of the two alleles, and short scaffolds contain fragments of the other alleles, which may explain many fragmented ORFs in the assembly as well as underestimation of the genome size by the k-mer genome size estimation and qPCR. Do the authors have any evidence to exclude this possibility?

Response to comment 1:

The DBG2OLC technique we employed is not unconventional. It has been used in over a dozen genome assemblies, including those published in GigaScience (golden mussel, European beech & Chinese herbal fleabane), Nature Genetics (apple & sea lamprey), Nature Plants (Xerophyta viscosa), Cell (Egyptian Rousette bat), and Genome Biology and Evolution (clam shrimp). Genome assembly is not a 'solved problem' and few (if any) assemblies use identical combinations of technology, sequencing depth and assembly/scaffolding methods. We agree that independent assembly of allelic variants is a potential issue for heterozygous non-haploid assemblies, and highlight it as a possible cause of the high ORF numbers (L225-7):

“artefactual duplications in the genome assembly, either through under-assembly or legitimate assembly of two heterozygous diploid copies;”

And L231-2:

“Of the 3,279 complete BUSCO genes identified (Table 2), only 85 (2.59%) were duplicated. This suggests that there is not widespread duplication in the assembly.”

In addition, extensive assembly of allelic variants would inflate the genome assembly size dramatically, and we see no evidence of this. We also see a trend in the data (consistent with the contiguity statistics) that fragmentation is a likely cause for inflated ORF counts (L264-9):

“Excluding proteins with annotation indicating possible viral or transposable element origin, 45.7% of "similar" proteins and 96.8% of “unknown” proteins had the same closest X.

tropicalis blastp hit as another predicted protein. Consistent with this being related to gene fragmentation, there was a negative relationship between the number of cane toad proteins sharing a given X. tropicalis top hit, and how much of the X. tropicalis hit was covered by each cane toad protein."

Although the evidence presented makes widespread duplicated assembly (allelic or otherwise) unlikely, we acknowledge that, as with all draft assemblies, there will be some scaffolds and ORFs that represent allelic variants. We have therefore added this caveat (L269-70):

"Nevertheless, it is likely that some of these protein fragments represent allelic variants that have been redundantly assembled." [additional text in manuscript]

There is no consistent way to globally identify and distinguish these from duplications, particularly in a repeat-rich genome like the cane toad. We have therefore opted to adopt a conservative filtering approach as detailed analysis of genes/regions of interest should identify any such issues on a case-by-case basis. As previously noted (and see point 4 below), we have unambiguously stated that our statistics refer to the assembly and we stop short of making unsubstantiated claims about the cane toad genome.
Impact on genome size is discussed in Point 2, below.

Comment 2:

In addition, the reported genome sizes of Rhinella marina (the same as Bufo marinus) varied between 3.98 and 5.65 Gb [26, 32-38]. Among the cited references, the papers by MacCulloch et al. (1996) and Chipman et al. (2001) appear to be reliable, because, in comparison with the genome size of Xenopus laevis (3.1 Gb), that of Bufo marinus was estimated to be 3.98 and 3.59, respectively, (the mean is 3.77 Gb) by assuming that 1pg DNA corresponds to 1 Gb. By the way, is Rhinella marina truly diploid? If so, its genome contains much more transposable elements and/or repetitive sequences than the allotetraploid genome of Xenopus laevis. According to the X. laevis genome paper (Session et al., 2016), total shotgun sequences in contigs (nucleotide stretches without N) are 2.45 Gb in allotetraploid X. laevis, which is similar to the final hybrid assembly of 2.55 Gb in diploid R. marina. This might imply again artificial sequence redundancy in the hybrid assembly due to allelic differences in wild R. marina. This may also explain the inconsistency between the flow cytometry-based genome size of 3.77 Gb and the k-mer-estimated genome size of ~2.0 Gb. Did the authors check artificial internal redundancy due to the two distinct alleles? The authors need to discuss this kind of issue in their paper.

Response to comment 2:

We have no evidence against diploidy in Rhinella marina and the published karyotype does not show evidence of higher ploidy. We did consider artificial sequence redundancy, however this would inflate the estimated genome size (and assembly), not reduce it, and so it cannot be the explanation for the observed differences. Likewise, if the qPCR primers were allele-specific (point 1), the apparent genome size would be doubled, not halved. The kmer method we used (GenomeScope) was a diploid method and explicitly incorporates

allelic variation into its estimation model. As readers may not be familiar with this method, we have expanded our discussion of this issue with an extra a sentence to emphasise this point (L176-7):

“GenomeScope explicitly models heterozygous diploid kmer distributions, which should make it robust to the additional challenge of sequencing a wild animal. However, GenomeScope predictions are affected by non-uniform repeat distributions and this difference could indicate high copy number repeats in the genome that are difficult to model accurately.” [additional text in manuscript]

Comment 3:

In Summary: According to the authors, "Annotation predicted 58,302 protein coding genes" include many fragmented ORFs. Because of this, the number (58,302) is meaningless, which should be removed from the summary. In the answer, the authors wrote "however many of these may be bona fide functional members of the cane toad proteome," but what is the rational to think like this? For example, what percentage of these ORFs are expressed? In general, such unexpressed ORFs are not counted as protein-coding genes. Therefore, the statement "however many of these may be bona fide functional members of the cane toad proteome" should be deleted if there is no supporting evidence.

Response to comment 3:

We have rephrased the sentence in the abstract (L62):

“Annotation predicted 25,846 protein coding genes with similarity to known proteins in SwissProt.” [modified text in manuscript]

The manuscript includes analysis and discussion of transcriptomic support for the predictions, including a warning that some of the 58,302 predicted protein coding genes may be false annotations (L242-254). The quoted statement refers to predicted proteins that may originate from transposable elements or viruses. Exaptation of transposons and endogenous viral elements is common in nature and we have no reason to believe that it will not have happened in the cane toad. We have expanded the expression analysis as suggested to support this statement (and moved it to follow discussion of the expression data), L251-4:

“Further review of the predicted protein descriptions revealed 4,357 with likely origins in transposable elements (including 4,114 LINE-1 ORFs) and 215 from viruses. However, many of these may be bona fide functional members of the cane toad proteome; 1,447 (33.2%) “transposon” and 151 (70.2%) of “viral” transcripts had support for expression > 1 TPM.” [additional text in manuscript]

Comment 4:

Fig. 5 (now Fig. 9) represents the feature of the assembly sequence, not the genome. The authors need to carefully state which it is in the figures, legends, and main text.

Response to comment 4:

This is clearly stated in the revised text and figure legend (emphasis added):

“RepeatMasker annotations from the MAKER pipeline support this interpretation, with over 4.1 million repeat sequences detected, accounting for 63.9% of the assembly (Table 5). The mean repeat length is 406 bp, which exceeds the Illumina read length used in our study (mean 140.6 bp paired-end). This makes short-read assembly of these regions difficult, as reflected by the poor ABySS contiguity (contig N50 = 583 bp, Table 2), and emphasises the need for long read data in this organism. The most abundant class of repeat elements are of unknown type (1.61 million elements covering 32.28% of the assembly), with DNA transposons the most abundant known class of element (817,262 repeats; 19.17% coverage). Of these, the most abundant are of the hAT-Ac (231,332 copies) and TcMar-Tc1 (226,145 copies) superfamilies (Table S4). Accounting for overlaps between repeat and gene features, 18.7% of the assembly (479,397,014 bp) has no annotation (Figure 9).”

The title of the figure is “Summary of the main annotation classes for Rhinella marina genome assembly.”

Close
