# Supplementary material for: Draft genome assembly of the invasive cane toad, Rhinella marina
Source: Gigascience. 2018 Aug 7;7(9):giy095. doi: 10.1093/gigascience/giy095 (PMC6145236; doi:10.1093/gigascience/giy095)
Supplement: Reviewer_1_Report_(Original_Submission) -- Rene L Warren — 4/9/2018 Reviewed [file giy095_reviewer_1_report_(original_submission).pdf]

Reviewer Report

Title: **Draft genome assembly of the invasive cane toad, Rhinella marina**

Version: **Original Submission** **Date:** 4/9/2018

Reviewer name: **Rene L Warren**

Reviewer Comments to Author:

Report GigaScience - Cane ToadRichard Edwards and colleagues present a manuscript describing the genome assembly and annotation of the invasive cane toad. The manuscript reads very well and the accompanied genomic resource is sorely needed; To this day, very few amphibian genome sequences are available. I found the paper succinct and to the point, especially the introduction. The methods are sound, comprehensive and well described. The bioinformatics approaches employed for genome assembly and annotation appear robust, although I have a concern about the short read sequencing library upon which this worked is based (below). The [qPCR] approach to estimate the genome size is clever and I appreciate the discussion on the disparity between various methodologies. Likewise, I welcome the discussion on the over inflation of MAKER gene predictions and have a recommendation (below). I understand the rationale for sequencing the genome of this invasive species and I am sure readers and scientists will be grateful for the resources presented and made freely available here. Where I think the authors fall short is on not reporting any insights about (or derived from) the genome (aside from repeat content), despite having the first-hand look at it. It is a data note and therefore no requirements for biological analyses, but surely something can be said about the genes you have predicted? E.g. any gene families stand out? Are there genes in your genome draft that could explain, at least partially, the enormous success this species has in non-native environments? For instance, what is known about the gene(s) involved in the production of the toxic secretions you mentioned? Are there any clues from the resources you are sharing with the community?I look at the supporting data available on the FTP server and everything checks out. I do have a recommendation for an additional file (see below). Main points:With this data note, the authors advertise the cane toad genome sequence and associated annotation, and make these resources available to the community - this is good. The authors claim that the draft genome "sets a milestone in the field of anuran genetics". I would like the authors to describe why it is so, in their conclusion.Typically, for genome papers, a high-confidence gene set is also reported/provided (in addition to what is presented, often based on AED and other criteria). A high confidence set would be a very useful resource to have, reduce the gene space in the process and present a more focused, gold standard list of better-annotated genes. This set stands a higher chance of yielding valuable and meaningful insights for your and future studies, set that would hold against scientific scrutiny (In the process weeding out the many, potentially spurious, gene predictions reported herein). The sentence on line 240 starting with "Critically.." is not accurate and needs to be re-worked. FYI Some short read assemblers are able to assemble through repeats larger than read length with the help of paired-end information. You could rephrase to something like "The average length (XX +/- Std. dev.) of most (XX%) of these repeat classes exceeds that of the Illumina reads used in our study (Paired-end 150bp), making the short read assembly difficult in these regions. This is reflected by the low assembly contiguity (contig N50 length = 583bp). " Though I must say that such a low contiguity figure is very untypical for an ABySS assembly, even for a highly repeated genome. Especially since your library captures sizes as long as 800bp. I am concerned about gDNA content/representation, as you seem to only have constructed a single paired-end library. Building multiple libraries from the same tissue source, preferably from 2 or more samples, prevent possible sampling/lab manipulation biases and ensures you have captured the entire genomic content. I also recommend building libraries of various insert sizes: 500, 2kbp, 5kbp whenever possible, especially when it is your only source of long-range information for assembly. This helps short read assemblers resolve repeats and increase the contiguity of the resulting assembly. Since you mainly used the ABySS short-read assembly for improving the accuracy of the DBG2OLC long read one, it might be ok in this case (especially since you recover many complete BUSCOs), but it also explains why a hybrid assembly approach does not improve the N50 length metric of the long-read DBG2OLC assembly -

where I think it should. Minor points:The cane toad reference transcriptome was published by the Authors and used as direct evidence for MAKER gene prediction. The Authors briefly mentioned it as a "multi-tissue" from tadpoles and adults. It would be good to provide more information (2-3 sentences) on this evidence in the present study (so readers readily know what went in the gene prediction tools), especially if that information could be used to gain insights on cane toad genetics. For example, stress tests designed to measure the cane toad response (eg. RNA-seq) in say a simulated change in climate/environment would allow one to zero-in on gene(s) that could play a key role in their adaptation, or even vulnerability in non-native environments. I am not suggesting additional experiments here, but rather a synopsis of the genes you predicted from the gene annotation procedure. line 219, typo, should read "Approximately"Make sure you report to single digit (or double) consistently, throughout.Rene Warren

Level of Interest

Please indicate how interesting you found the manuscript: An article whose findings are important to those with closely related research interests

Quality of Written English

Please indicate the quality of language in the manuscript: Acceptable

Declaration of Competing Interests

Please complete a declaration of competing interests, considering the following questions:

- Have you in the past five years received reimbursements, fees, funding, or salary from an organisation that may in any way gain or lose financially from the publication of this manuscript, either now or in the future?
- Do you hold any stocks or shares in an organisation that may in any way gain or lose financially from the publication of this manuscript, either now or in the future?
- Do you hold or are you currently applying for any patents relating to the content of the manuscript?
- Have you received reimbursements, fees, funding, or salary from an organization that holds or has applied for patents relating to the content of the manuscript?
- Do you have any other financial competing interests?
- Do you have any non-financial competing interests in relation to this paper?

If you can answer no to all of the above, write 'I declare that I have no competing interests' below. If your reply is yes to any, please give details below.

I declare that I have no competing interests

I agree to the open peer review policy of the journal. I understand that my name will be included on my report to the authors and, if the manuscript is accepted for publication, my named report including any attachments I upload will be posted on the website along with the authors' responses. I agree for my

report to be made available under an Open Access Creative Commons CC-BY license (http://creativecommons.org/licenses/by/4.0/). I understand that any comments which I do not wish to be included in my named report can be included as confidential comments to the editors, which will not be published.

I agree to the open peer review policy of the journal

To further support our reviewers, we have joined with Publons, where you can gain additional credit to further highlight your hard work (see: https://publons.com/journal/530/gigascience). On publication of this paper, your review will be automatically added to Publons, you can then choose whether or not to claim your Publons credit. I understand this statement.Yes
