# Supplementary material for: Draft genome assembly of the invasive cane toad, Rhinella marina
Source: Gigascience. 2018 Aug 7;7(9):giy095. doi: 10.1093/gigascience/giy095 (PMC6145236; doi:10.1093/gigascience/giy095)
Supplement: Reviewer_2_Report_(Original_Submission) -- Taejoon Kwon — 4/15/2018 Reviewed [file giy095_reviewer_2_report_(original_submission).pdf]

Reviewer Report

Title: Draft genome assembly of the invasive cane toad, Rhinella marina

Version: Original Submission **Date:** 4/15/2018

Reviewer name: Taejoon Kwon

Reviewer Comments to Author:

Here Edward et al., reported the draft genome of cane toad Rhinella marina. Although there are several genomes recently published, still amphibian is highly understudied in genomics, so I think this data would be valuable resource to expand our knowledge in many perspectives, including the evolutionary history of this species' invasion in new environment. Authors analyzed their data thoughtfully, and released all data in public repository, so I think this manuscript is satisfactory for GigaScience as a Data Note. However, there are some points required to be clarify before publication (see below), so I think authors should revise the following points before publication:(1) Although BUCSO analysis can be used for genome completeness, it is based on protein coding genes, so I think 'Assessment of genome completeness' would be better to be merged with 'Genome annotation and gene prediction' section. (2) In previous publication with R. marina transcriptome (Richardson, et al., GigaScience, 2018; doi: 10.1093/gigascience/gix114; Ref #18 on current manuscript), it was reported that 1.7% of BUCSO genes were fragmented, and 7.4% of them were missing on their 62,202 CDS transcripts. These numbers look better than genome-based result described in this manuscript (7.5% of fragmented, and 9.5% of missing). Authors may need to discuss the difference among these two annotations.(3) The analysis of 'unknown function' genes with published de novo transcriptome (p.9 line 229-) seems to have a circularity. Authors used all RNA-seq data already on their annotation, which are also used for de novo transcriptome construction (p.9 line 206). So instead of analyzing their matched length, I recommend to analyze their expression level from RNA-seq data. If 'unknown function' genes were mis-annotated genes as authors thought, it should have lower level of evidence for expression, compared to 'known function' genes. Some minor points:(1) '3 s.f' (significant figure) notation on table headers make the reader confused. It is obvious to recognize by looking at numbers on table, so it would be better to remove it. (2) In Table 4, qPCR value is also the average of two experiments (p.8, line 190-191), so it would be fair to present min/max values for that.

Level of Interest

Please indicate how interesting you found the manuscript: Choose an item.

Quality of Written English

Please indicate the quality of language in the manuscript: Choose an item.

Declaration of Competing Interests

Please complete a declaration of competing interests, considering the following questions:

- Have you in the past five years received reimbursements, fees, funding, or salary from an organisation that may in any way gain or lose financially from the publication of this manuscript, either now or in the future?

- Do you hold any stocks or shares in an organisation that may in any way gain or lose financially from the publication of this manuscript, either now or in the future?
- Do you hold or are you currently applying for any patents relating to the content of the manuscript?
- Have you received reimbursements, fees, funding, or salary from an organization that holds or has applied for patents relating to the content of the manuscript?
- Do you have any other financial competing interests?
- Do you have any non-financial competing interests in relation to this paper?

If you can answer no to all of the above, write 'I declare that I have no competing interests' below. If your reply is yes to any, please give details below.

I declare that I have no competing interests.

I agree to the open peer review policy of the journal. I understand that my name will be included on my report to the authors and, if the manuscript is accepted for publication, my named report including any attachments I upload will be posted on the website along with the authors' responses. I agree for my report to be made available under an Open Access Creative Commons CC-BY license (http://creativecommons.org/licenses/by/4.0/). I understand that any comments which I do not wish to be included in my named report can be included as confidential comments to the editors, which will not be published.

I agree to the open peer review policy of the journal

To further support our reviewers, we have joined with Publons, where you can gain additional credit to further highlight your hard work (see: https://publons.com/journal/530/gigascience). On publication of this paper, your review will be automatically added to Publons, you can then choose whether or not to claim your Publons credit. I understand this statement. Yes
