# Supplementary material for: Draft genome assembly of the invasive cane toad, Rhinella marina
Source: Gigascience. 2018 Aug 7;7(9):giy095. doi: 10.1093/gigascience/giy095 (PMC6145236; doi:10.1093/gigascience/giy095)
Supplement: Reviewer_3_Report_(Original_Submission) -- Masanori Taira — 4/24/2018 Reviewed [file giy095_reviewer_3_report_(original_submission).pdf]

Reviewer Report

Title: **Draft genome assembly of the invasive cane toad, Rhinella marina**

Version: **Original Submission** **Date:** 4/24/2018

Reviewer name: **Masanori Taira**

Reviewer Comments to Author:

This paper describes a draft sequence of the whole genome of the cane frog, Rhinella marina. Most quality controls are well done, but "Genome annotation and gene prediction" is relatively poor. Major comments1) p. 11; line 226: The authors identified 32,456 genes with unknown function in addition to the 25,846 predicted genes. The number of these unknown genes seem to much more than expected, but their explanation for it is insufficient. They mentioned that the median length is 171 aa, but what is the cut-off length of amino acids, and what is their range (the minimum and maximum)? In which regions in the genome sequence are those genes located? That is, are those genes scattered in the unique sequence in the genome or localized in the regions with repetitive sequences, transposable elements, or some other specific sequences? If the authors use the same strategy of pipelines for gene annotation with the X. laevis and X. tropicalis genome sequences, how many genes with unknown function could be identified and what percentage of them could be orthologous to those of R. marina?2) Figure 5: The authors need to compare the data in Figure 5 with those of other amphibian species. Minor comments1) Is Rhinella marina the same as Rhinella marinus and Bufo marinus? The authors need to describe this in the abstract and introduction for clarification. 2) The genome size usually means the size of haploid DNA, but, in the text and table, the authors mentioned "a haploid genome size." When the authors simply use "the genome size," does this mean "a haploid genome size?" If so, better not to use "a haploid genome size." 3) p. 10, line 179: If PCR conditions are nothing special, those could be written in the legend of Tables or Figures, or deposited to "protocol.io."4) The authors should include s.f. and other abbreviations, if any, that are not listed, in the list of abbreviations.

Level of Interest

Please indicate how interesting you found the manuscript: Choose an item.

Quality of Written English

Please indicate the quality of language in the manuscript: Choose an item.

Declaration of Competing Interests

Please complete a declaration of competing interests, considering the following questions:

- Have you in the past five years received reimbursements, fees, funding, or salary from an organisation that may in any way gain or lose financially from the publication of this manuscript, either now or in the future?
- Do you hold any stocks or shares in an organisation that may in any way gain or lose financially from the publication of this manuscript, either now or in the future?

- Do you hold or are you currently applying for any patents relating to the content of the manuscript?
- Have you received reimbursements, fees, funding, or salary from an organization that holds or has applied for patents relating to the content of the manuscript?
- Do you have any other financial competing interests?
- Do you have any non-financial competing interests in relation to this paper?

If you can answer no to all of the above, write 'I declare that I have no competing interests' below. If your reply is yes to any, please give details below.

I declare that I have no competing interests.

I agree to the open peer review policy of the journal. I understand that my name will be included on my report to the authors and, if the manuscript is accepted for publication, my named report including any attachments I upload will be posted on the website along with the authors' responses. I agree for my report to be made available under an Open Access Creative Commons CC-BY license (http://creativecommons.org/licenses/by/4.0/). I understand that any comments which I do not wish to be included in my named report can be included as confidential comments to the editors, which will not be published.

I agree to the open peer review policy of the journal

To further support our reviewers, we have joined with Publons, where you can gain additional credit to further highlight your hard work (see: https://publons.com/journal/530/gigascience). On publication of this paper, your review will be automatically added to Publons, you can then choose whether or not to claim your Publons credit. I understand this statement.Yes
