# Supplementary material for: Draft genome assembly of the invasive cane toad, Rhinella marina
Source: Gigascience. 2018 Aug 7;7(9):giy095. doi: 10.1093/gigascience/giy095 (PMC6145236; doi:10.1093/gigascience/giy095)
Supplement: Reviewer_3_Report_(Revision_2) -- Masanori Taira — 7/15/2018 Reviewed [file giy095_reviewer_3_report_(revision_2).pdf]

Reviewer Report

Title: Draft genome assembly of the invasive cane toad, Rhinella marina

Version: Revision 2 Date: 7/15/2018

Reviewer name: Masanori Taira

Reviewer Comments to Author:

CommentsThis manuscript adopted "a hybrid de novo whole genome assembly strategy," a relatively new technique, which should require more quality controls than the conventional technique combining shotgun sequences, mate-pair sequences, PacBio long read sequences, and Hi-C or CHICAGO methods. Most comments from the reviewers including mine for basic analyses with the assembly are for quality controls, not for analyses "beyond the scope of this paper," as the authors say. Furthermore, they used a wild-caught frog, which must contain distinct alleles, probably making assembly processes difficult. How did the authors overcome allelic differences? I'm worried about one undesired possibility that the current assembly contains one of the two alleles, and short scaffolds contain fragments of the other alleles, which may explain many fragmented ORFs in the assembly as well as underestimation of the genome size by the k-mer genome size estimation and qPCR. Do the authors have any evidence to exclude this possibility?In addition, the reported genome sizes of Rhinella marina (the same as Bufo marinus) varied between 3.98 and 5.65 Gb [26, 32-38]. Among the cited references, the papers by MacCulloch et al. (1996) and Chipman et al. (2001) appear to be reliable, because, in comparison with the genome size of Xenopus laevis (3.1 Gb), that of Bufo marinus was estimated to be 3.98 and 3.59, respectively, (the mean is 3.77 Gb) by assuming that 1pg DNA corresponds to 1 Gb. By the way, is Rhinella marina truly diploid? If so, its genome contains much more transposable elements and/or repetitive sequences than the allotetraploid genome of Xenopus laevis. According to the X. laevis genome paper (Session et al., 2016), total shotgun sequences in contigs (nucleotide stretches without N) are 2.45 Gb in allotetraploid X. laevis, which is similar to the final hybrid assembly of 2.55 Gb in diploid R. marina. This might imply again artificial sequence redundancy in the hybrid assembly due to allelic differences in wild R. marina. This may also explain the inconsistency between the flow cytometry-based genome size of 3.77 Gb and the k-mer-estimated genome size of ~2.0 Gb. Did the authors check artificial internal redundancy due to the two distinct alleles? The authors need to discuss this kind of issue in their paper. About previous major comments Point 1) In Summary: According to the authors, "Annotation predicted 58,302 protein coding genes" include many fragmented ORFs. Because of this, the number (58,302) is meaningless, which should be removed from the summary. In the answer, the authors wrote "however many of these may be bona fide functional members of the cane toad proteome," but what is the rational to think like this? For example, what percentage of these ORFs are expressed? In general, such unexpressed ORFs are not counted as protein-coding genes. Therefore, the statement "however many of these may be bona fide functional members of the cane toad proteome" should be deleted if there is no supporting evidence. Point 2) Fig. 5 (now Fig. 9) represents the feature of the assembly sequence, not the genome. The authors need to carefully state which it is in the figures, legends, and main text.

Level of Interest

Please indicate how interesting you found the manuscript: Choose an item.

Quality of Written English

Please indicate the quality of language in the manuscript: Choose an item.

Declaration of Competing Interests

Please complete a declaration of competing interests, considering the following questions:

- Have you in the past five years received reimbursements, fees, funding, or salary from an organisation that may in any way gain or lose financially from the publication of this manuscript, either now or in the future?
- Do you hold any stocks or shares in an organisation that may in any way gain or lose financially from the publication of this manuscript, either now or in the future?
- Do you hold or are you currently applying for any patents relating to the content of the manuscript?
- Have you received reimbursements, fees, funding, or salary from an organization that holds or has applied for patents relating to the content of the manuscript?
- Do you have any other financial competing interests?
- Do you have any non-financial competing interests in relation to this paper?

If you can answer no to all of the above, write 'I declare that I have no competing interests' below. If your reply is yes to any, please give details below.

I declare that I have no competing interests.

I agree to the open peer review policy of the journal. I understand that my name will be included on my report to the authors and, if the manuscript is accepted for publication, my named report including any attachments I upload will be posted on the website along with the authors' responses. I agree for my report to be made available under an Open Access Creative Commons CC-BY license (http://creativecommons.org/licenses/by/4.0/). I understand that any comments which I do not wish to be included in my named report can be included as confidential comments to the editors, which will not be published.

I agree to the open peer review policy of the journal

To further support our reviewers, we have joined with Publons, where you can gain additional credit to further highlight your hard work (see: https://publons.com/journal/530/gigascience). On publication of this paper, your review will be automatically added to Publons, you can then choose whether or not to claim your Publons credit. I understand this statement. Yes
