# Supplementary material for: Draft genome assembly of the invasive cane toad, Rhinella marina
Source: Gigascience. 2018 Aug 7;7(9):giy095. doi: 10.1093/gigascience/giy095 (PMC6145236; doi:10.1093/gigascience/giy095)
Supplement: Reviewer_4_Report_(Original_Submission) -- John Malone — 4/24/2018 Reviewed [file giy095_reviewer_4_report_(original_submission).pdf]

Reviewer Report

Title: **Draft genome assembly of the invasive cane toad, Rhinella marina**

Version: **Original Submission** **Date:** 4/24/2018

Reviewer name: **John Malone**

Reviewer Comments to Author:

Edwards et al. report a draft assembly of the marine toad genome; only the sixth species of amphibian to be assembled and the first from the family Bufonidae. Amphibian genomes remain under-represented relative to other vertebrates and this contribution provides a welcome addition to fill in an important gap at the base of tetrapods. The toad is an invasive species and the genome assembly should prove useful to understand the genetics of invasive species. The authors take a hybrid assembly approach and mix a single sized 350 bp fragment Illumina library with larger fragment PacBio libraries. They extracted DNA from liver from an adult female. Liver is known to endoreduplicate, which can create rearrangements and problems for de novo assembly projects. However, BUSCO analysis indicates that many of the single genes have been identified in the assembly and their results are comparable to X. tropicalis, arguably the most well assembled and annotated amphibian genome available. Despite their high quality BUSCO results, the draft marine toad genome is highly fragmented. Hopefully, the authors will build upon this draft and create a reference that can have more widespread applicability for comparative genomic analysis. As the authors note, the bar is quite low for amphibians because of high repeat content which can create problems for assembly. To date, there are only two amphibian genomes assembled to chromosome scale and both are species of Xenopus. They used ABySS to assemble the genome but given that this genome note format is highly technical, it might be useful to report comparisons with other assemblers that they no doubt tried and/or provide more explanation for using ABySS relative to other assemblers.Regarding their metrics in Table 2. I was confused by the %N reporting for their assembly and long read libraries. The authors report 0.0% of the assembly is in gaps, which is surprising given how repetitive amphibian genomes are, how poorly assembled the toad genome is (though comparably poor to other amphibians which have Ns) and nearly all vertebrate genome assemblies (including the human genome) have some bases unresolved and/or in gaps marked by a series of Ns. The proportion in gaps is an important metric of assembly quality. If the genome really does not have any Ns, it might be useful to highlight this unique attribute somewhere in the text and provide some explanation for how they were able to eliminate gaps. Their k-mer genome size estimation analysis shows the effect of kmer size and quality trimming but remains far from the estimated genome size based on flow cytometry and other experiments. The authors follow this up with a nice qPCR experiment and provide explanation for how far they are off. Given that the genome assembly size deviates substantially from the reported size, I would worry about using this assembly to analyze repeat content (as the authors state in the manuscript). As an additional

confirmatory experiment to help build confidence in their results, I wonder if a synteny analysis with Xenopus tropicalis would be useful. Such a comparison might help reveal more about overall synteny and/or continuity and further strengthen their assembly results. Line 193-199: Here there is discussion about first estimate of genome size using either k-mer or qPCR analysis. This is not the first genome size estimate based on kmer distributions. Perhaps the authors want to state that this is the first amphibian genome estimated in this way? Maybe downplaying this sentence, or more clearly defining what they want to say here would be useful. There are a number of sentences in the text that oversell the results abit and these should be corrected (for example: line 54-55---consider eliminating the line about iconic status and major gaps in understanding cane toad genetics…..this is the case for nearly all organisms; line 248---the fragmented draft assembly, early stage protein-coding annotation results, and estimates that deviate from expectation is contributing to additional fragmented amphibian assemblies; a milestone should go further than what is reported in the manuscript). The authors use MAKER2 for their gene annotation pipeline combined with their reference transcriptome. Given their abundant RNA-Seq, I was surprised that they did not use BRAKER1, which typically provides superior annotations compared to MAKER2. This might explain why it appears they have highly over-predicted the number of genes in the toad genome, though it could also stem from poor assembly. MAKER is widely used but their abundant RNA-Seq data is perfect for using BRAKER1 and they may obtain superior annotations using this tool. In some locations of the text, genus and species are italicized, in other locations they are not. Fix according to journal format requirements.

Level of Interest

Please indicate how interesting you found the manuscript: Choose an item.

Quality of Written English

Please indicate the quality of language in the manuscript: Choose an item.

Declaration of Competing Interests

Please complete a declaration of competing interests, considering the following questions:

- Have you in the past five years received reimbursements, fees, funding, or salary from an organisation that may in any way gain or lose financially from the publication of this manuscript, either now or in the future?
- Do you hold any stocks or shares in an organisation that may in any way gain or lose financially from the publication of this manuscript, either now or in the future?
- Do you hold or are you currently applying for any patents relating to the content of the manuscript?

- Have you received reimbursements, fees, funding, or salary from an organization that holds or has applied for patents relating to the content of the manuscript?
- Do you have any other financial competing interests?
- Do you have any non-financial competing interests in relation to this paper?

If you can answer no to all of the above, write 'I declare that I have no competing interests' below. If your reply is yes to any, please give details below.

I declare that I have no competing interests

I agree to the open peer review policy of the journal. I understand that my name will be included on my report to the authors and, if the manuscript is accepted for publication, my named report including any attachments I upload will be posted on the website along with the authors' responses. I agree for my report to be made available under an Open Access Creative Commons CC-BY license (http://creativecommons.org/licenses/by/4.0/). I understand that any comments which I do not wish to be included in my named report can be included as confidential comments to the editors, which will not be published.

I agree to the open peer review policy of the journal

To further support our reviewers, we have joined with Publons, where you can gain additional credit to further highlight your hard work (see: https://publons.com/journal/530/gigascience). On publication of this paper, your review will be automatically added to Publons, you can then choose whether or not to claim your Publons credit. I understand this statement. Yes
